# Dissecting the Effects of Cage Structure in the Catalytic Activation of Imide Chlorenium-Ion Donors

**DOI:** 10.1021/jacs.5c01249

**Published:** 2025-03-21

**Authors:** Hang Zhou, Tomasz K. Piskorz, Keyu Liu, Yining Lu, Fernanda Duarte, Paul J. Lusby

**Affiliations:** † EaStCHEM School of Chemistry, University of Edinburgh, Joseph Black Building, David Brewster Road, Edinburgh, Scotland EH9 3FJ, U.K.; ‡ Chemistry Research Laboratory, University of Oxford, Oxford OX1 3TA, U.K.

## Abstract

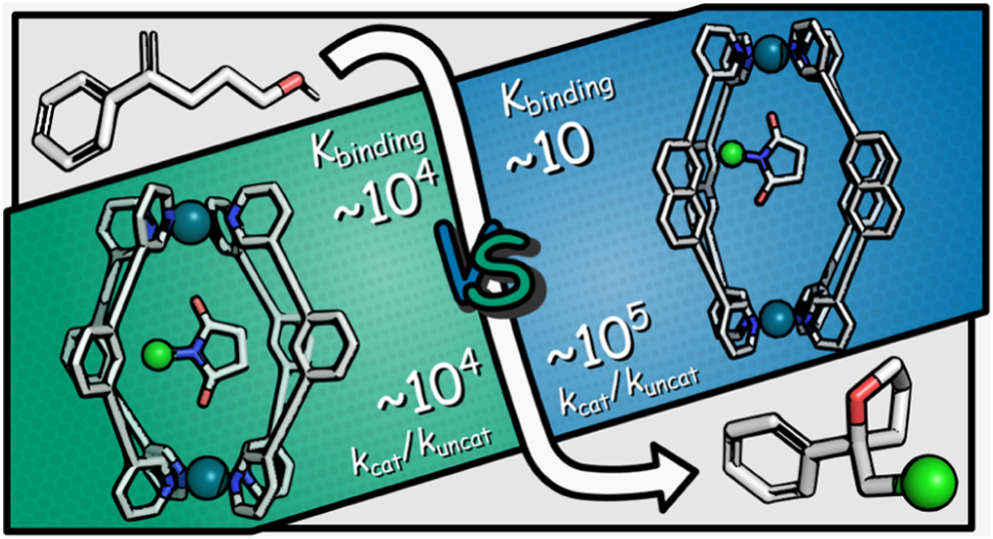

Imide-based chlorinating reagents are mild and easy to use yet can lack the reactivity of charged chlorenium-ion donors. Here, we present a simple strategy for increasing the reactivity of these neutral chlorinating species by encapsulation inside a cationic coordination cage. Using this approach, we demonstrate that two different-sized Pd_2_L_4_ cages can catalyze chlorolactonization and chlorocycloetherification reactions of acid and alcohol functionalized α and β-substituted styrene substrates with either 1,3-dichloro-5,5-dimethylhydantoin (DCDMH) or *N*-chlorosuccinimide (NCS) as the chlorenium sources. A kinetic study shows that the cages are proficient catalysts with a significant acceleration up to 10^5^. However, an unexpected dichotomy is revealed wherein the smaller cage, which is best preorganized to bind and nominally provide maximum activation of the imide reagent, shows an order of magnitude less acceleration than the larger cage that has apparently mismatched host–guest chemistry. When the scope of reactions is further extended to the chlorination of simple, unfunctionalized α-methylstyrene, the same pattern of cage reactivity is observed, suggesting that differences are not explained by coencapsulation. Computational studies indicate that the trend in reactivity is caused by the transition state being less fixed in the larger cage, allowing it to find optimal binding and thereby generate stronger interactions. This investigation highlights the importance of understanding the underlying mechanisms of cage reactivity to design new noncovalent catalysts for a greater range of transformations.

## 1 Introduction

Despite the many examples of ever more structurally complex self-assembled metallo-organic cages,^1−19^ their use as catalysts remains relatively limited to a few privileged structures.^20−26^ In principle, cages should be excellent catalysts; their three-dimensional structure provides a platform to engineer collections of noncovalent interactions, akin to the way that an enzyme active site positions multiple amino acids to selectively bind specific substrates and facilitate their conversion into products. The modular synthesis of cages should make it relatively easy to tailor the structure so that it is optimal for different transformations. It is perhaps surprising then that the number of studies that study how changes to the cage structure affect catalytic performance remain rare.^27−29^ This is perhaps one reason that cage catalysis as a whole remains relatively underdeveloped.

One of the reoccurring features of coordination cage-mediated reactivity is the way these systems exploit the proximity of the charged metal vertices to the substrate binding pocket,^30,31^ complementing other examples of supramolecular catalysts that use either electrostatics,^32−40^ or rely on the preorganization of substrate(s).^41−45^ The use of charge was first clearly demonstrated by the groups of Raymond and Bergman, who showed that the binding of weakly basic substrates inside an anionic cage shifts the propensity to become protonated by several p*K*_a_ units,^46^ thereby allowing reactions that are normally acid-catalyzed to proceed at high pH.^47−49^ Latterly, several groups have shown that cationic cages can achieve catalysis in much the same way by stabilizing pathways that involve anionic species,^50−52^ leading to acidification of substrates thereby facilitating reaction under neutral as opposed to basic conditions.^53−55^ Additionally, pioneering work by Ward and co-workers has shown how the charged periphery of the cage can attract Coulombically complementary reactants, thus eliciting rate enhancement through high effective concentration.^23,56,57^

An approach that has rarely been used in cage catalysis is to enhance the reactivity of an electrophile through stabilization of the leaving group.^58^ Most powerful electrophiles possess an electronically stable anionic leaving group, which is commonly the conjugate anion of a strong Brønsted acid. Alternatively, cationic electrophiles are reactive, as the leaving group is a stable, neutral molecule. We envisaged that we could complement these strategies using noncovalent binding of a less reactive neutral electrophile within a cationic supramolecular cage. This *in situ* activation of the electrophile provides the obvious advantage that it avoids the handling of inherently reactive species. While this approach has the potential to be applicable to many different reaction types, here we choose to demonstrate this concept using the electrophilic chlorination of olefins. Interestingly, our results with two different active cages highlight that matching the host–guest chemistry to the electrophile leaving group is only part of the role an effective catalyst plays. Instead, we show that flexible transition state (TS) binding is key, as this maximizes interactions with the leaving group and stabilizes the reduction in nucleophile electron density that occurs during the course of the transformation.

## 2 Results and Discussion

### 2.1 Establishing Cage-Activated Chlorenium-Ion Donor Catalysis

**Scheme 1:**
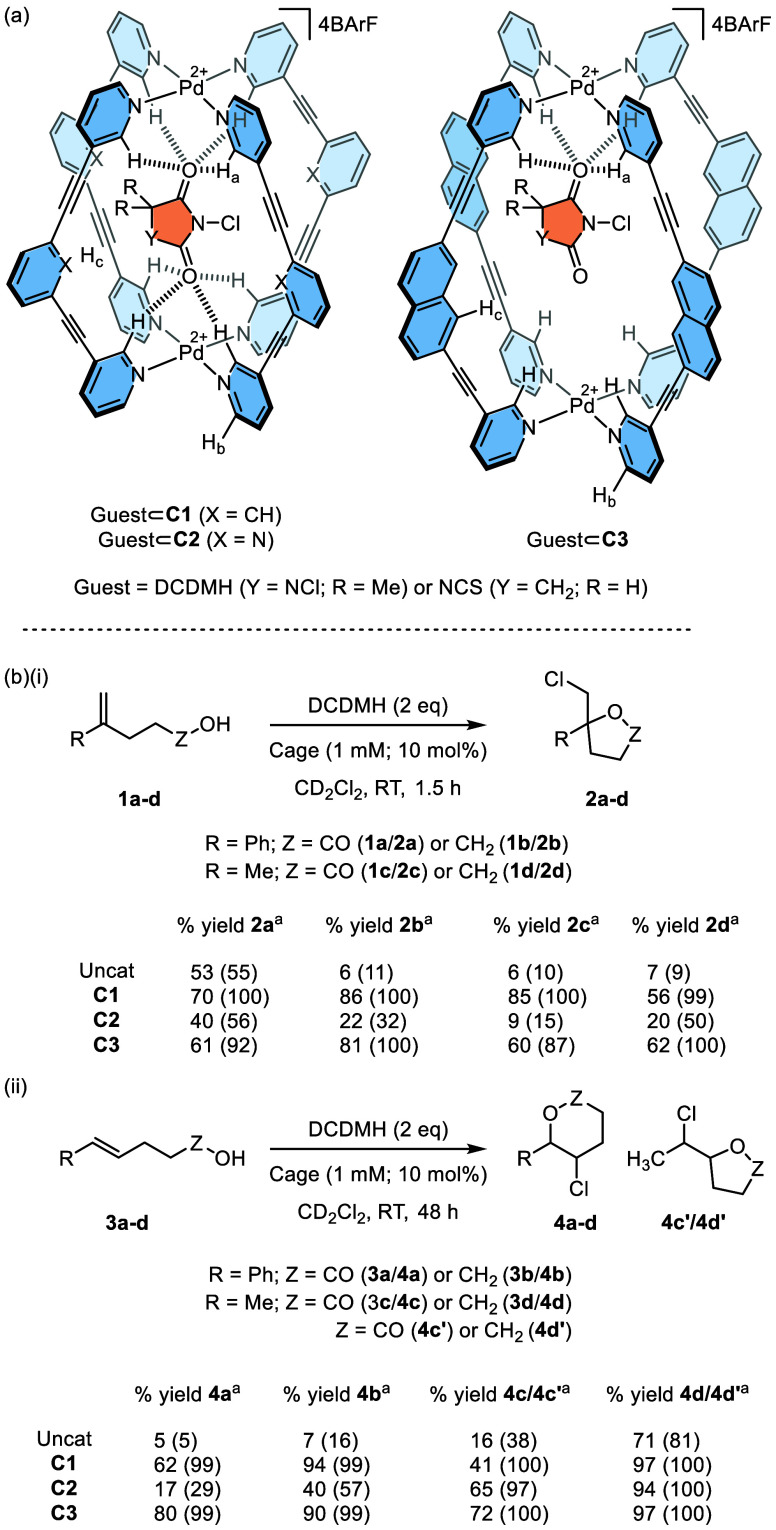
(a) Cage-Activated Chloro-Imide Host-Guest Complexes and (b) Chlorenium-Induced Cyclization Catalysis All yields were determined by ^1^H NMR integration. Conversion (% starting material consumption) is given in parentheses.

Cages **C1** and **C2** are predisposed to bind certain dicarbonyl compounds due to the spatial arrangement of the H-bond donor pockets that are positioned at either end of the cage (Scheme 1a).^59^ This attribute has previously been exploited to enhance the Diels–Alder reactivity of quinones, leading to significant acceleration and efficient turnover.^60^ We therefore reasoned that **C1** and **C2** would bind and activate neutral, mild chlorinating agents such as N-chlorosuccinimide (NCS) and 1,3-dichloro-5,5-dimethylhydantoin (DCDMH; Scheme 1a). Inspired by the work of Borhan,^61^ we initially chose to investigate this concept using the chlorolactonization of substrate **1a** (Scheme 1b(i)). We were encouraged to see that treating **1a** with 2 equiv of DCDMH in the presence of 10 mol % **C1** resulted in a modestly improved yield of **2a** after 1.5 h at room temperature in CD_2_Cl_2_. The better yield appeared limited to **C1**, with **C2** giving approximately the same amount of product as that of the uncatalyzed reaction. The conversion of alcohol substrate **1b** to chlorinated cyclic ether **2b** gave a much clearer increase in yield from 6% in the uncatalyzed reaction to 86% in the presence of **C1**. The methyl analogues **1c**/**1d** also showed a noticeable improvement in the yield, particularly when **C1** was added to the reaction. The less reactive β-substituted styrene substrates **3a** and **3b** produced the corresponding chlorinated cyclized products **4a** and **4b** in 62 and 94% yields,^62^ respectively, in the presence of **C1**, whereas the equivalent background reactions gave <10% product. In all cases, the **C2**-mediated reactions gave noticeably lower yields. The methyl analogues of the β-substituted styrenes, **3c** and **3d**, also showed clear improvements in yield with notably faster consumption of the substrate in the presence of cage (Figure S19), generating a mixture of 5- and 6-membered ring products.

We have previously found that the activity of **C1** and **C2** is highly transformation dependent; **C1** is a catalyst for Michael addition yet does not promote Diels–Alder reactions, and *vice versa*.^53,59^ For Michael addition, this is a consequence of their anion-binding properties; **C1** strongly encapsulates charged guests, whereas the noncoordinating pyridyl lone pairs of **C2** significantly neutralize the electrostatic potential of the cavity, meaning anion binding is much less favored. It was therefore expected that **C1** would be the better catalyst for electrophilic chlorination due to stabilization of the imidate leaving group. What we were surprised to find was that when we expanded the investigation to the larger cage **C3**, this gave yields comparable to those of **C1** (Scheme 1b). This was unforeseen as the larger naphthyl spacer of **C3** increases the Pd–Pd distance by over 2 Å relative to **C1**,^63^ meaning that **C3** should be mismatched to bind DCDMH through both sets of carbonyl groups (Scheme 1a) therefore imparting less activation.

The substrate scope also reveals that the yields of chlorinated cyclic ethers are uniformly higher than those of the equivalent lactones. However, it should be noted that all **C1** and **C3** catalyzed reactions show complete consumption of the starting materials. This difference in mass balance appears to be caused by the formation of small quantities of byproducts, which we tentatively ascribe to vinylic chloride acyclic compounds (see Supporting Information).

### 2.2 Experimental Quantification of Cage-Catalyzed Chlorocycloetherification

**Figure 1:**
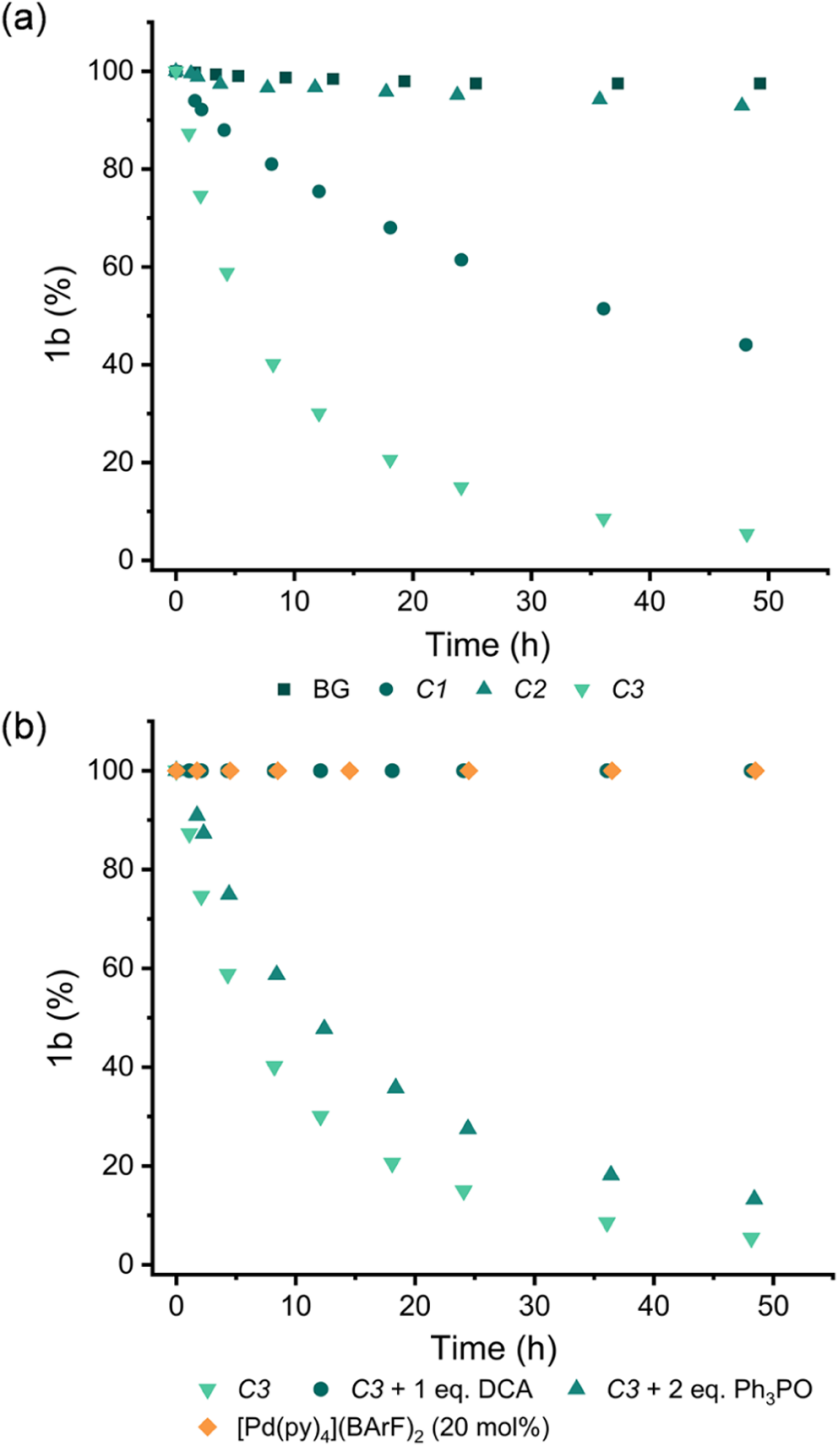
Kinetic data for the NCS-mediated reaction of **1b** → **2b**, showing (a) a comparison of background vs cage-catalyzed reactions; (b) control reactions for the **C3** catalyzed process. The reaction conditions are the same as those shown in Scheme 1 with NCS substituted for DCDMH. Triphenylphosphine oxide binds to the outside of the cage (*K*_a_ = 390 M^–1^), while DCA (9,10-dicyanoanthracene) is a strong internal guest (*K*_a_ = 3.3 × 10^4^ M^–1^).^63^

Seeking to gain a better understanding of the cage reactivity, we decided to focus on the conversion of **1b** → **2b**. Attempts to monitor the reaction over time revealed that acceleration was too fast to easily follow; ^1^H NMR spectra acquired immediately after reactant mixing showed that the **C1** and **C3** reactions were essentially finished. To overcome this problem, we substituted DCDMH for the less reactive NCS, leading to reactions that could be conveniently monitored over hours. This data clearly showed a reactivity pattern of **C3** > **C1** ≫ **C2** ≈ uncatalyzed (Figure 1a).

Various control experiments have been carried out to show that the activity stems from the encapsulation of NCS (Figures 1b and S20). Adding strong binding guests to the **C1** and **C3** reactions—pentecenedione and 9,10-dicycanoanthracene for the smaller and larger cages,^59,63^ respectively—halts catalysis. In contrast, when triphenylphosphine oxide was added, which selectively binds to the outer H_b_ protons (Figures S32 and S33),^64^ only a marginal reduction in activity was observed. When the cages were replaced by [Pd(Py)_4_](BArF)_2_ (Py = pyridine) to provide a similar H-bond motif but without a cavity,^53^ again no catalysis was observed (Figure 1b).

**Figure 2:**
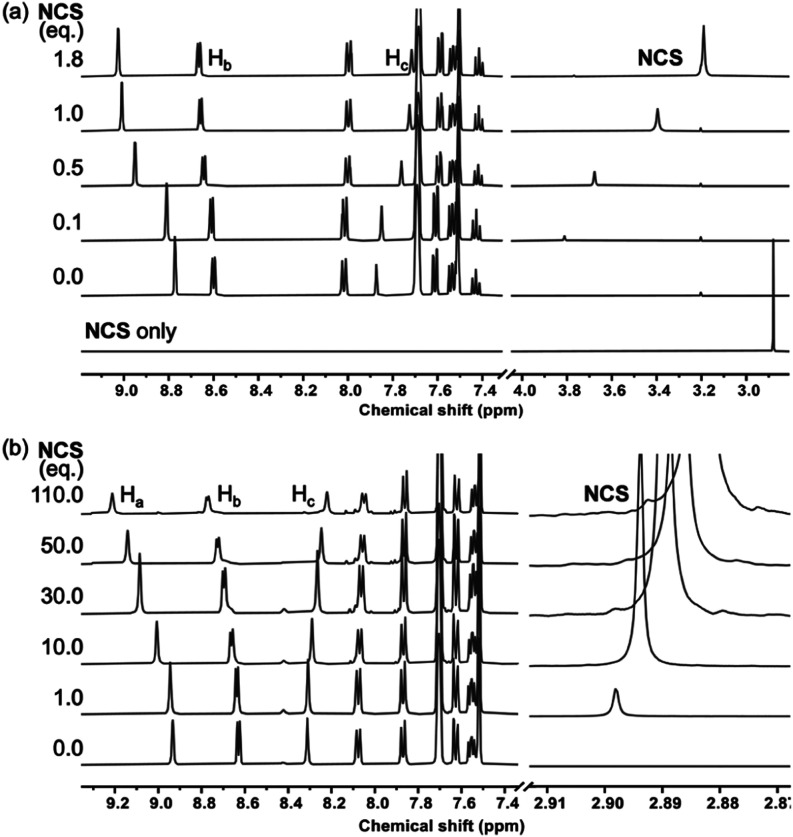
Partial ^1^H NMR (400 MHz, CD_2_Cl_2_, 300 K) spectra for the titrations of NCS into (a) **C1** and (b) **C3**. The identities of protons H_a_, H_b_, and H_c_ are shown in Scheme 1.

To further probe the differences between **C1** and **C3**, we performed host–guest titration and saturation kinetics experiments. Gradual addition of NCS to either cage produces a single set of ^1^H NMR signals that shift as a function of equivalents (Figure 2a,b), indicating exchange that is fast on the NMR time scale. The chemical shift changes are also consistent with encapsulation; the inward-facing H-bond donor protons (H_a_) move significantly downfield (Δδ > 0.3 ppm), and the “equatorial” H_c_ protons become shielded. While the maximal shifts in the signals of the two cages are similar, the number of equivalents needed to reach cage saturation is significantly different; 1 mM solutions of **C1** and **C3** require 2 and 100 equiv of NCS, respectively. Fitting the titration data to a 1:1 binding model (Figures S29 and S31) reveals that, as expected, **C1** binds NCS much more strongly (*K*_a_ = 3.3 × 10^4^ M^–1^) compared to **C3** (*K*_a_ = 32 M^–1^). The chemical shift changes in the NCS are also markedly different. At low equivalents with respect to the cage, where the NCS signal is averaged toward the fully bound species, there is a significant difference in the amplitude of the chemical shift; the signals of NCS inside **C1** and **C3** are shifted by 1 ppm and <0.1 ppm, respectively. While this is not a direct measure of the electrophilicity of the chlorine atom, it nonetheless suggests that NCS is significantly more electron deficient inside **C1** compared to **C3**. The ^1^H NMR chemical shifts of the substrate **1b** shows no discernible changes in the presence of **C1** or **C3** cage, either in the absence or presence of NCS, indicating that any interaction is at most only transient.

**Figure 3:**
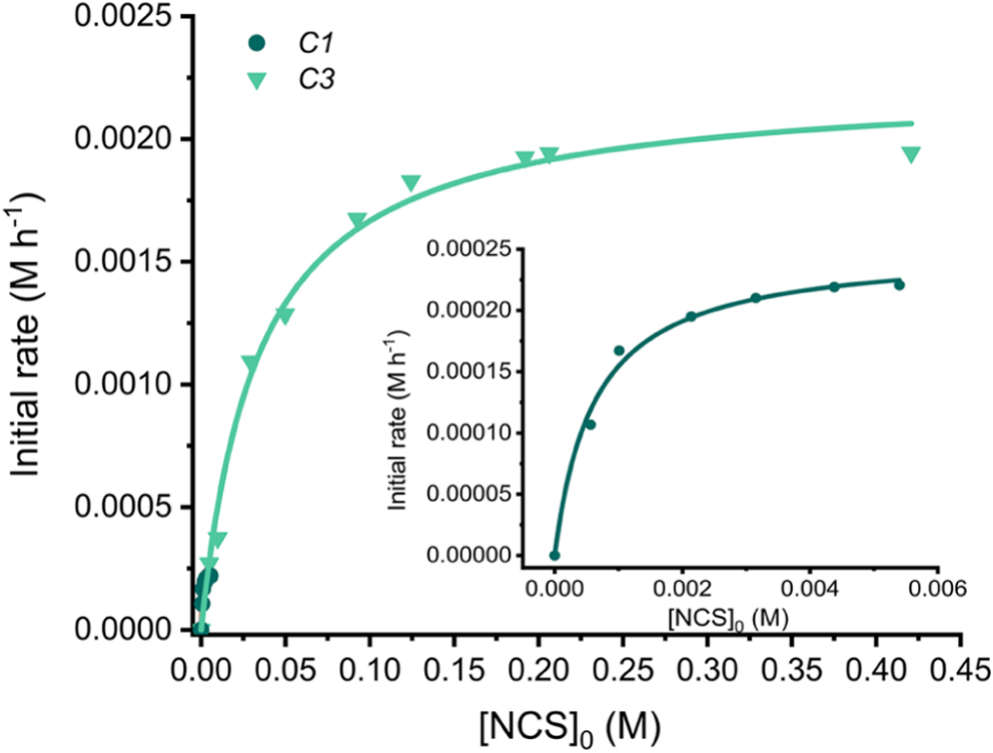
Initial rate as a function of variable [NCS]_0_ for the **C1** and **C3** catalyzed conversions of **1b** to **2b**. The solid lines are fits to the Michaelis–Menten equation. Reaction conditions are the same as those in Scheme 1b.

As expected, the large difference in association constants for **C1** and **C3** leads to a pronounced disparity in saturation kinetics; plots of initial rate versus variable [NCS]_0_ at fixed [cage] and [**1b**] (Figure 3) show that ν_max_ is attained at significantly higher [NCS]_0_ for **C3** compared to **C1**. This data also provides a very good fit to the Michaelis-Menten eq (Figure 3). As the out rate of the NCS is much quicker than *k*_cat_ (based on the fast NMR exchange), then *K*_m_ serves as a proxy for 1/*K*_a_ (i.e., the dissociation constant). This is indeed the case for both **C1** and **C3** (Table 1), which again highlights the fact that the catalytic properties of the cage are intrinsically linked to NCS encapsulation. Significantly, the *k*_cat_/*k*_uncat_ value for **C3** is an order of magnitude higher than **C1** (Table 1).

**Table 1:** Michaelis-Menten Parameters for the **C1** and **C3** Catalyzed Conversion of **1b** to **2b** with NCS^a^

	*K*_m_ (M)	1/*K*_a_ (M^–1^)^a^	*k*_cat_ (M^–1^ s^–1^)	*k*_cat_/*k*_uncat_
**C1**	6.3 × 10^–4^	3.0 × 10^–5^	7.4 × 10^–3^	3.7 × 10^4^
**C3**	3.4 × 10^–2^	3.1 × 10^–2^	6.0 × 10^–2^	3.2 × 10^5^

a Values obtained from ^1^H NMR titration.

### 2.3 Computational Modeling of Cage-Catalyzed Chlorocycloetherification

**Figure 4:**
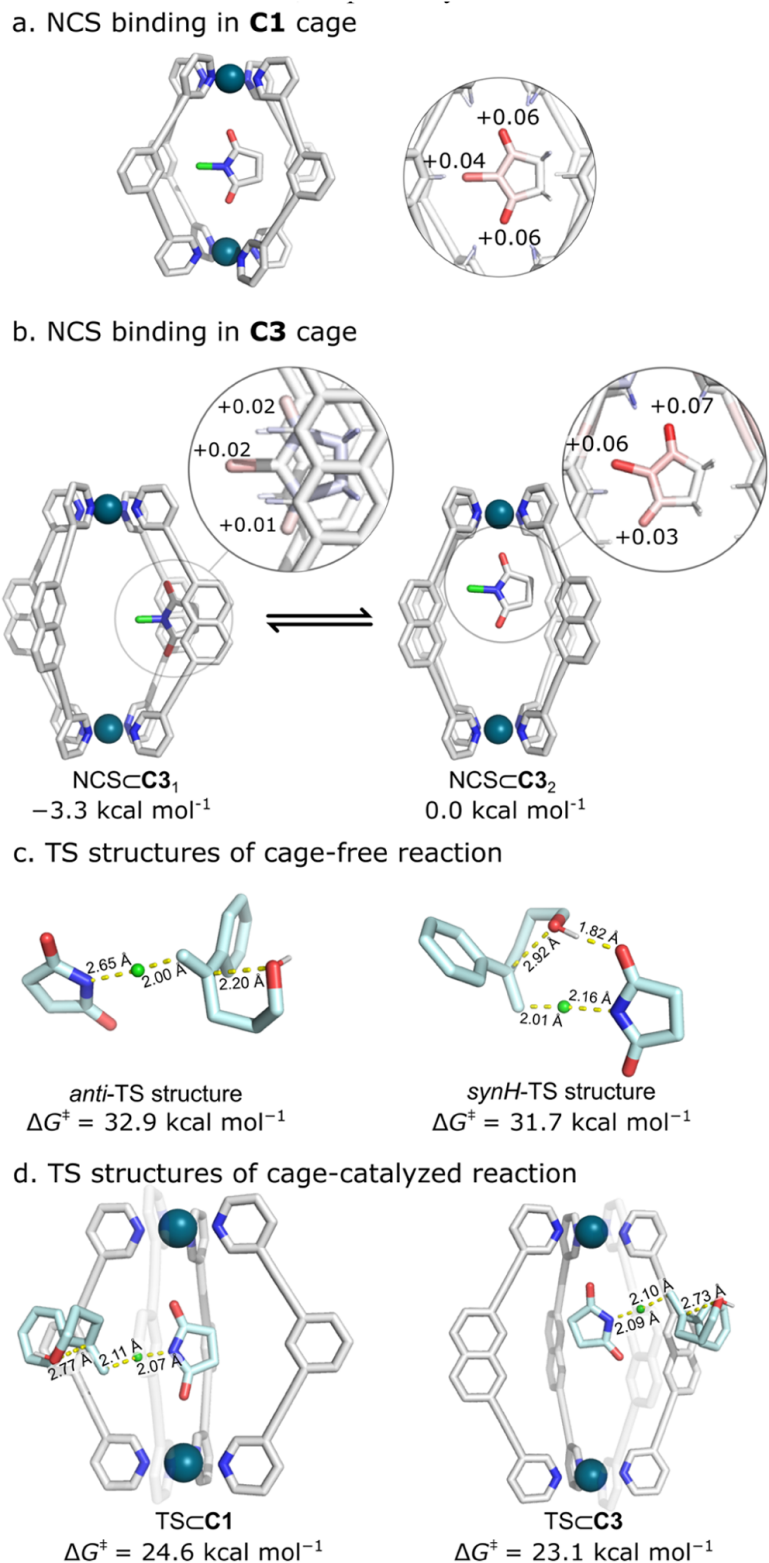
Computational analysis of the chlorocycloetherification reaction. (a,b) DFT-optimized structures obtained from MD trajectory for (a) NCS ⊂ **C1** and (b) NCS ⊂ **C3**. The magnified view shows changes in selected Hirshfield charges of bound vs unbound NCS. TS structures for the (c) cage-free and (d) **C1** and **C3** catalyzed reactions. Calculations were performed at the CPCM(DCM)-M06–2X/def2-TZVP//CPCM(DCM)-PBEh-3c level of theory.

To better understand the molecular origins of catalysis, we turned to computational modeling, in particular molecular dynamics (MD) and density theory functional (DFT) calculations. In the absence of an X-ray crystal structure, we initially modeled the binding of NCS to cages **C1** and **C3** using conventional MD in explicit dichloromethane (DCM) with four BArF^–^ counterions and 17 water molecules to most accurately represent experimental conditions. We then extracted representative frames from these simulations and optimized them using DFT (SI, Section 6). This analysis revealed that the distance between the two Pd ions in **C1** is indeed well suited for binding NCS through interactions between the two NCS oxygen atoms and the C–H bond donor pockets at each end of the cage (Figure 4a). As also expected, the Pd–Pd distance for **C3** is too large to allow both carbonyl groups of NCS to simultaneously interact with the two C–H hydrogen-bond donor pockets. Instead, the predominant complex observed by MD, NCS ⊂ **C3**_1_, has NCS positioned symmetrically between two adjacent naphthyl groups (Figure 4b), with both oxygens too far away from the H_a_ atoms to form hydrogen bonds. Such a binding mode is at odds with the NMR results, which show significant deshielding of the H_a_ signal. DFT calculations of a host–guest complex, NCS ⊂ **C3**_2_, in which one oxygen atom of NCS interacts with a single H-bond donor pocket of the cage, shows that this is 3.3 kcal mol^–1^ less stable than NCS ⊂ **C3**_1_ (Figure 4b). However, given the strong experimental evidence, we selected NCS ⊂ **C3**_2_ as the reference for further calculations involving **C3**. Surprisingly, despite the apparent “double” and “single” activation that the smaller and larger cages provide, both cages polarize NCS similarly, inducing total net charges of +0.17 and +0.19 on NCS when bound to **C1** and **C3**, respectively.

DFT calculations were further used to model the mechanism of the uncatalyzed reaction and the reaction within the cages (Figure 4c/d). Building on earlier studies of chlorolactonization,^65^ we identified two transition states (TSs), referred to as *synH-* and *anti-*TS. Both TSs in solution show comparable high energies at room temperature, with Δ*G*^‡^ = 31.7 and 32.9 kcal mol^–1^ for the *syn-* and *anti-*TS, respectively (Figure 4c). Both cages decreased the activation energy, with the *anti-*TSs showing a lower activation energy (Δ*G*^‡^ = 24.6 and 23.1 kcal mol^–1^ for **C1** and **C3**, respectively) than the *synH*-TSs (Δ*G*^‡^ = 26.1 and 24.8 kcal mol^–1^ for **C1** and **C3**) (Figure 4d). While the computed activation barriers in solution and in the cages are consistently higher by about 4–5 kcal mol^–1^ than the experimental values (derived by converting the observed rates to energies using the Eyring equation), the observed trends in rate enhancement follow those from experiments. For **C1** and **C3**, the reduction in activation energy between the uncatalyzed reaction and the cage (ΔΔ*G*^‡^ = Δ*G*_cage_^‡^ – Δ*G*_uncat_^‡^) is −7.1 and −8.6 kcal mol^–1^, respectively, which is within 1 kcal mol^–1^ of the experimental results (ΔΔ*G*^‡^ = −6.3 and −7.6 kcal mol^–1^, respectively). Examination of the electronic contribution to the reaction energy revealed that binding of NCS within the C–H bond pockets of the cages, NCS ⊂ **C1** and NCS ⊂ **C3**_**2**_, activate NCS similarly by lowering the lowest unoccupied molecular orbital (LUMO) energy by 0.4 eV (Table S9). A similar effect was observed for the Diels–Alder reaction of benzoquinone in **C2**.^66^

### 2.4 Dissecting the Effects of Different Cage Structures

**Scheme 2:**
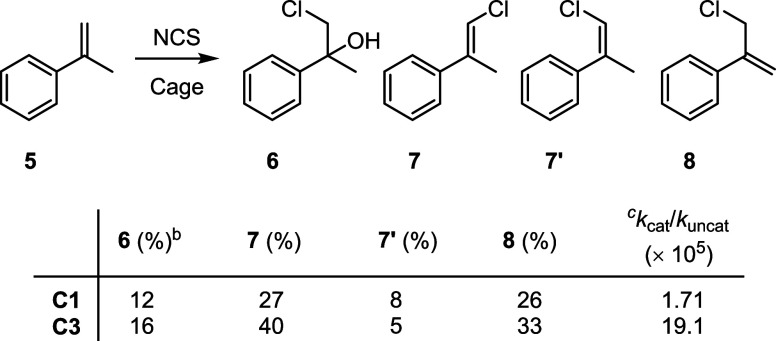
Cage-Catalyzed Chlorination of α-Methylstyrene Reaction conditions: 5 (10 mM), NCS (200 mM), cage (1 mM), CD_2_Cl_2_, RT, 48 h. Product yields determined by ^1^H NMR integration. Product 6 arises from residual water present in the reaction. Based on the consumption of substrate **5**.

The data obtained from the kinetic, host–guest, and modeling analysis lead to the obvious question: despite the similar polarization of NCS, why is **C3** a more active catalyst than **C1**? A hypothesis that we initially considered was that the larger cavity of **C3** could facilitate transient coencapsulation of **1b** (i.e., not readily detectable by ^1^H NMR) through interactions of the substrate alcohol group with the single “free” hydrogen-bond pocket of the cage in the NCS ⊂ **C3**_2_ structure. We have previously observed a similar phenomenon for Michael addition catalysis, wherein acceleration is a consequence of binding both the nucleophile and the electrophile.^54^ To explore this possibility, we decided to study the chlorination of α-methylstyrene, **5**, which lacks any obvious polar functional group that can hydrogen bond with the cage (Scheme 2). Both **C1** and **C3** catalyze the chlorination of this substrate, generating products **6**-**8**. Under NCS saturation conditions, the magnitude of the acceleration with both cages is 10-fold higher than that observed for **1b** → **2b**, with again **C3** providing greater acceleration (*k*_cat_/*k*_uncat_) than **C1** by one order of magnitude. This indicates that **C3** is a generally better chlorination catalyst (i.e., not specific to the reaction of **1b** → **2b**) and thus is not a consequence of the formation of a ternary Michaelis complex such as **1b**·NCS⊂**C3**.

The key rate-determining step in the reaction of **5** is the heterolytic transfer of the chlorenium ion from the cage-bound NCS to the substrate. In the absence of a cage, this transfer has a computed activation barrier of 33.1 kcal mol^–1^, while the experimentally derived value is 27.1 kcal mol^–1^. In **C1** and **C3**, the computed energy barriers decrease to 25.3 and 25.1 kcal mol^–1^, respectively, with experimental values of 19.9 and 18.5 kcal mol^–1^. Similarly to the chlorocycloetherification reaction (Figure 4), there are consistently higher energy barriers of about 5–6 kcal mol^–1^. However, the computationally derived reduction in the energy barrier of **C1** and **C3** by 7.8 and 8.0 kcal mol^–1^, respectively, is in close agreement with the values obtained experimentally (7.2 and 8.6 kcal mol^–1^ for **C1** and **C3**, respectively).

**Figure 5:**
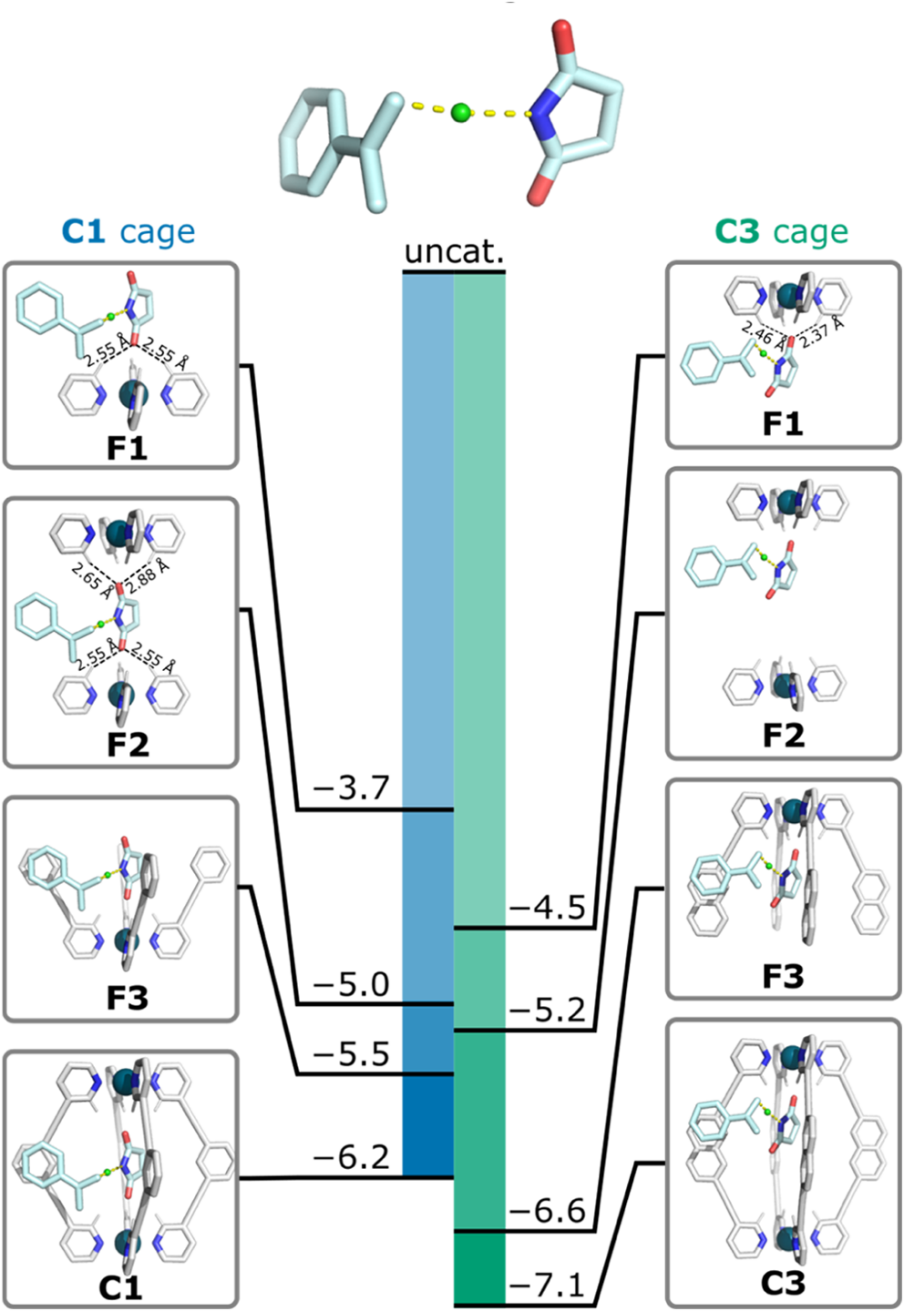
Contributions of fragments **F1**–**F3** of cages **C1** and **C3** to the lowered electronic activation barrier (ΔΔ*E*^‡^). The energy contributions were calculated for geometries obtained by following the internal reaction coordinate (IRC) for **C1** and **C3** cage-catalyzed reactions at the def2-TZVP//CPCM(DCM)-PBEh-3c level of theory and subsequent partitioning of the cage into the desired fragment (hydrogen atoms were added to the unsaturated carbon atoms). The single point energies were calculated at CPCM(DCM)-M06–2X/def2-TZVP.

To assess how different parts of the cage contribute to the observed catalytic activity and thus also better understand the structure–activity relationship, we partitioned the cage-catalyzed TS and reactant complex (RC) structures into various fragments (Figure 5). The electronic activation barrier for each of these fragments was computed (Δ*E*^‡^), which in turn was used to give the reduction in the electronic energy of the TS relative to the uncatalyzed reaction (ΔΔ*E*^‡^ = Δ*E*_cage_^‡^ – Δ*E*_uncat_^‡^). The fragments used in this analysis corresponded to a single Pd(Py)_4_^2+^ unit (**F1**); two nonconnected Pd(Py)_4_^2+^ units (**F2**); a single Pd(L)_4_^2+^ unit, where L is a ligand containing the middle aromatic ring connected to the pyridyl group *via* an alkyne spacer (**F3**). From this modeling, the following observations are made:

(a) Comparison of **F1** for the two cages shows that the “single-site” interaction between one Pd(Py)_4_^2+^ unit and the TS is stronger in **C3** compared to **C1**, with ΔΔ*E*^‡^ = −4.5 and −3.7 kcal mol^–1^, respectively. This is in line with the shorter C–H···O distances in **C3** (2.41 ± 0.07 Å) vs. **C1** (2.56 ± 0.03 Å). Quantification of this interaction *via* natural bond orbital (NBO) analysis indicates these H-bond interactions contribute 4.2 kcal mol^–1^ stabilization in **C3** and only 2.3 kcal mol^–1^ in **C1** (Table S13).
(b) The **F2** fragments, consisting of two noninterconnected Pd(Py)_4_^2+^ units, are overall very similar for **C1** and **C3** (ΔΔ*E*^‡^ = – 5.0 and – 5.2 kcal mol^–1^, respectively). However, comparing **F1** and **F2** for both cages shows that the consequences of adding the second Pd(Py)_4_^2+^ group are quite different. In the case of **C3**, the larger Pd–Pd distance only allows the TS to interact with the cage through one oxygen atom, leading to only a small decrease in ΔΔ*E*^‡^ from −4.5 and −5.2 kcal mol^–1^. However, even with **C1**, where both oxygen atoms of the TS interact, the decrease from −3.7 to −5.0 kcal mol^–1^ shows that the two Pd(Py)_4_^2+^ groups provide a noticeably unequal contribution to catalysis. This is even clearer through NBO analysis, which shows that the H-bond interactions of the “first” Pd(Py)_4_^2+^ group provide 2.3 kcal mol^–1^ stabilization energy and the second one only 0.9 kcal mol^–1^. This is also in line with the different C–H···O distances for both sites, 2.56 ± 0.03 Å vs 2.75 ± 0.07Å (Figure 5). A similar NBO analysis for **C3** shows that the interaction energies of **F1** and **F2** remain constant at 4.2 kcal mol^–1^.
(c) The **F3** fragments effectively represent “half” of a cage, consisting of the most strongly interacting Pd(Py)_4_^2+^ unit and four ligand “arms” that include the central aromatic rings. Comparing the **F3** and **F1** fragments indicates that the inclusion of these “arms” makes a significant contribution to TS stabilization, providing an extra 1.8 kcal mol^–1^ for **C1** (total – 5.5 kcal mol^–1^) and 2.1 kcal mol^–1^ for **C3** (total **–**6.6 kcal mol^–1^). It is also notable that greater secondary stabilization for both cages comes not through interactions with an additional Pd(Py)_4_^2+^ unit but rather through the interactions of the TS and the aromatic surfaces of the ligand.

This fragment-based analysis sheds significant light on both the origins of catalysis and points toward why **C3** is a better catalyst than **C1**. Clearly, the major contribution to catalysis in both cages is the stabilization of the TS through hydrogen-bond interactions with one of the Pd(Py)_4_^2+^ groups. However, the interactions with the ligand framework also point to a mechanism in which activity is leveraged from the synergistic stabilization of charge transfer that occurs as the reaction progresses toward the TS. In other words, the Pd(Py)_4_^2+^ groups stabilize the buildup of electron density on the imidate leaving group while the ligands facilitate the formation of the chloronium (or carbocation) intermediate. The importance of the structure of the cage as a whole is also supported by experiment; if activity was the result of interactions with only one Pd(Py)_4_^2+^ group then, in theory, catalysis could also occur through binding to the outer surface of the cage (i.e., through H-bonding to the H_b_ protons). However, the addition of an external inhibitor only marginally reduces activity, whereas the internal guest shuts down catalysis (Figure 1). Additionally, if catalysis was occurring externally, then it would be likely that **C1** and **C3** would display similar acceleration. The lack of activity from [Pd(Py)_4_](BArF)_2_ (Figure 1b) provides further compelling evidence that catalysis is not derived from the interaction with the coordination sphere of a single metal ion.

The most noticeable difference between **C1** and **C3** appears to stem from how the “secondary” Pd ion affects interactions with the TS. In **C3**, binding through only one of the oxygen atoms provides conformational flexibility that allows the strength of this interaction to be maximized. In contrast, the TS in **C1** is more fixed within the cage by the simultaneous binding of both oxygen atoms with the two Pd(Py)_4_^2+^ groups. This appears to reduce the maximal stabilization by a single site. Another possible consequence of this fixed versus flexible binding of the TS is that secondary interactions with the ligand framework may be reduced in **C1** compared to **C3**. Of course, the larger π-surface of the naphthyl spacer in **C3** may also enhance this secondary effect.

## 3 Conclusions

In conclusion, we have shown that Pd_2_L_4_ cages can act as highly effective noncovalent catalytic activators for mild neutral chlorinating agents such as DCDMH and NCS, facilitating the transformation of styrene derivatives into cyclic and acyclic chlorinated products. The acceleration exhibited by these is comparable to many of the best cage catalysts so far described.^23,47,54^ More importantly, however, this work contributes to the understanding of how cage catalysis operates and how structural modifications can impact activity. We continue to seek to expand these interconnected strands in the pursuit of growing the area of bioinspired supramolecular catalysis to create ever more active catalysts for a range of different transformations using only the power of noncovalent interactions.

## Supporting Information Available

This contains all experimental and methodology details including reaction monitoring, kinetic data and fitting, NMR titrations, and computational modeling. The Supporting Information is available free of charge at https://pubs.acs.org/doi/10.1021/jacs.5c01249.

This contains all experimental and methodology details including reaction monitoring, kinetic data and fitting, NMR titrations, and computational modeling (PDF)
density theory functional (DFT) (ZIP)
molecular dynamics (MD) (ZIP)

## Abbreviations

NCS: *N*-chlorosuccinamide
DCDMH: 1,3-dichloro-5,5-dimethylhydantoin

## References

[ref1] Chakrabarty R.; Mukherjee P. S.; Stang P. J. Supramolecular coordination: self-assembly of finite two-and three-dimensional ensembles. Chem. Rev. 2011, 111, 6810–6918. 10.1021/cr200077m. 21863792 PMC3212633

[ref2] Cook T. R.; Stang P. J. Recent Developments in the Preparation and Chemistry of Metallacycles and Metallacages via Coordination. Chem. Rev. 2015, 115, 7001–7045. 10.1021/cr5005666. 25813093

[ref3] Domoto Y.; Abe M.; Genov G. R.; Yu Z.; Fujita M. Interconversion of Highly Entangled Polyhedra into Concave Polyhedra by Nitrate-Induced Ternary Coordination. Angew. Chem., Int. Ed. 2023, 62, e202303714 10.1002/anie.202303714. 37139584

[ref4] Davies J. A.; Ronson T. K.; Nitschke J. R. Triamine and Tetramine Edge-Length Matching Drives Heteroleptic Triangular and Tetragonal Prism Assembly. J. Am. Chem. Soc. 2024, 146, 5215–5223. 10.1021/jacs.3c11320. 38349121 PMC10910536

[ref5] Wu K.; Benchimol E.; Baksi A.; Clever G. H. Non-statistical assembly of multicomponent [Pd_2_ABCD] cages. Nat. Chem. 2024, 16, 584–591. 10.1038/s41557-023-01415-7. 38243023

[ref6] Preston D.; Evans J. D. A Lantern-Shaped Pd(II) Cage Constructed from Four Different Low-Symmetry Ligands with Positional and Orientational Control: An Ancillary Pairings Approach. Angew. Chem., Int. Ed. 2023, 62, e202314378 10.1002/anie.202314378. 37816684

[ref7] Howlader P.; Zangrando E.; Mukherjee P. S. Self-Assembly of Enantiopure Pd_12_ Tetrahedral Homochiral Nanocages with Tetrazole Linkers and Chiral Recognition. J. Am. Chem. Soc. 2020, 142, 9070–9078. 10.1021/jacs.0c03551. 32315163

[ref8] Molinska P.; Tarzia A.; Male L.; Jelfs K. E.; Lewis J. E. M. Diastereoselective Self-Assembly of Low-Symmetry PdL_2_ Nanocages through Coordination-Sphere Engineering. Angew. Chem., Int. Ed. 2023, 62, e202315451 10.1002/anie.202315451. PMC10952360 37888946

[ref9] Lisboa L. S.; Preston D.; McAdam C. J.; Wright L. J.; Hartinger C. G.; Crowley J. D. Heterotrimetallic Double Cavity Cages: Syntheses and Selective Guest Binding. Angew. Chem., Int. Ed. 2022, 61, e202201700 10.1002/anie.202201700. PMC9310627 35194905

[ref10] Li R. -J.; de Montmollin J.; Fadaei-Tirani F.; Scopelliti R.; Severin K. Construction of Pd-based coordination cages with three geometrically distinct ligands. Dalton Trans. 2023, 52, 6451–6456. 10.1039/D3DT00248A. 37092605

[ref11] Barber B. E.; Jamieson E. M. G.; White L. E. M.; McTernan C. T. Metal-peptidic cages—Helical oligoprolines generate highly anisotropic nanospaces with emergent isomer control. Chem 2024, 10, 2792–2806. 10.1016/j.chempr.2024.05.002.

[ref12] Black M. R.; Bhattacharyya S.; Argent S. P.; Pilgrim B. S. Structural Transformations of Metal–Organic Cages through Tetrazine-Alkene Reactivity. J. Am. Chem. Soc. 2024, 146, 28233–28241. 10.1021/jacs.4c08591. 39236092 PMC11487605

[ref13] Abe T.; Sanada N.; Takeuchi K.; Okazawa A.; Hiraoka S. Assembly of Six Types of Heteroleptic Pd_2_L_4_ Cages under Kinetic Control. J. Am. Chem. Soc. 2023, 145, 28061–28074. 10.1021/jacs.3c09359. 38096127 PMC10755705

[ref14] Ube H.; Endo K.; Sato H.; Shionoya M. Synthesis of Hetero-multinuclear Metal Complexes by Site-Selective Redox Switching and Transmetalation on a Homo-multinuclear Complex. J. Am. Chem. Soc. 2019, 141, 10384–10389. 10.1021/jacs.9b04123. 31189315

[ref15] Yazaki K.; Akita M.; Prusty S.; Chand D. K.; Kikuchi T.; Sato H.; Yoshizawa M. Polyaromatic molecular peanuts. Nat. Commun. 2017, 8, 15914 10.1038/ncomms15914. 28656977 PMC5493752

[ref16] Parbin M.; Sivalingam V.; Chand D. K. Highly Anisotropic Pd_2_L_2_^ab^L_2_^cc^ and Pd_2_L_2_^ab^L_2_^cd^ Type Cages by Heteromeric Completive Self-Sorting. Angew. Chem., Int. Ed. 2024, 63, e202410219 10.1002/anie.202410219. 38949846

[ref17] Hugenbusch D.; Lehr M.; von Glasenapp J.-S.; McConnell A. J.; Herges R. Light-Controlled Destruction and Assembly: Switching between Two Differently Composed Cage-Type Complexes. Angew. Chem., Int. Ed. 2023, 62, e202212571 10.1002/anie.202212571. PMC10099457 36215411

[ref18] Bell D. J.; Zhang T.; Geue N.; Rogers C. J.; Barran P. E.; Bowen A. M.; Natrajan L. S.; Riddell I. A. Hexanuclear Ln_6_L_6_ Complex Formation by Using an Unsymmetric Ligand. Chem. - Eur. J. 2023, 29, e202302497 10.1002/chem.202302497. 37733973 PMC10946940

[ref19] Montà-González G.; Bastante-Rodríguez D.; García-Fernández A.; Lusby P. J.; Martínez-Máñez R.; Martí-Centelles V. Comparing organic and metallo-organic hydrazone molecular cages as potential carriers for doxorubicin delivery. Chem. Sci. 2024, 15, 10010–10017. 10.1039/D4SC02294G. 38966373 PMC11220577

[ref20] Fiedler D.; Bergman R. G.; Raymond K. N. Supramolecular Catalysis of a Unimolecular Transformation: Aza-Cope Rearrangement within a Self-Assembled Host. Angew. Chem., Int. Ed. 2004, 43, 6748–6751. 10.1002/anie.200461776. 15558640

[ref21] Yoshizawa M.; Tamura M.; Fujita M. Diels-Alder in Aqueous Molecular Hosts: Unusual Regioselectivity and Efficient Catalysis. Science 2006, 312, 251–254. 10.1126/science.1124985. 16614218

[ref22] Samanta D.; Mukherjee S.; Patil Y. P.; Mukherjee P. S. Self-Assembled Pd_6_ Open Cage with Triimidazole Walls and the Use of Its Confined Nanospace for Catalytic Knoevenagel- and Diels–Alder Reactions in Aqueous Medium. Chem. - Eur. J. 2012, 18, 12322–12329. 10.1002/chem.201201679. 22899180

[ref23] Cullen W.; Misuraca M. C.; Hunter C. A.; Williams N. H.; Ward M. D. Highly efficient catalysis of the Kemp elimination in the cavity of a cubic coordination cage. Nat. Chem. 2016, 8, 231–236. 10.1038/nchem.2452. 26892554

[ref24] Wang Q.-Q.; Gonell S.; Leenders S. H. A. M.; Dürr M.; Ivanović-Burmazović I.; Reek J. N. H. Self-assembled nanospheres with multiple endohedral binding sites pre-organize catalysts and substrates for highly efficient reactions. Nat. Chem. 2016, 8, 225–230. 10.1038/nchem.2425. 26892553

[ref25] Holloway L. R.; Bogie P. M.; Lyon Y.; Ngai C.; Miller T. F.; Julian R. R.; Hooley R. J. Tandem Reactivity of a Self-Assembled Cage Catalyst with Endohedral Acid Groups. J. Am. Chem. Soc. 2018, 140, 8078–8081. 10.1021/jacs.8b03984. 29913069

[ref26] Guo J.; Fan Y. Z.; Lu Y. L.; Zheng S. P.; Su C. Y. Visible-Light Photocatalysis of Asymmetric [2 + 2] Cycloaddition in Cage-Confined Nanospace Merging Chirality with Triplet-State Photosensitization. Angew. Chem., Int. Ed. 2020, 59, 8661–8669. 10.1002/anie.201916722. 32011801

[ref27] Hart-Cooper W. M.; Zhao C.; Triano R. M.; Yaghoubi P.; Ozores H. L.; Burford K. N.; Toste F. D.; Bergman R. G.; Raymond K. N. The effect of host structure on the selectivity and mechanism of supramolecular catalysis of Prins cyclizations. Chem. Sci. 2015, 6, 1383–1393. 10.1039/C4SC02735C. 29560226 PMC5811099

[ref28] Hong C. M.; Morimoto M.; Kapustin E. A.; Alzakhem N.; Bergman R. G.; Raymond K. N.; Toste F. D. Deconvoluting the Role of Charge in a Supramolecular Catalyst. J. Am. Chem. Soc. 2018, 140, 6591–6595. 10.1021/jacs.8b01701. 29767972

[ref29] Bierschenk S. M.; Pan J. Y.; Settineri N. S.; Warzok U.; Bergman R. G.; Raymond K. N.; Toste F. D. Impact of Host Flexibility on Selectivity in a Supramolecular Host-Catalyzed Enantioselective aza-Darzens Reaction. J. Am. Chem. Soc. 2022, 144, 11425–11433. 10.1021/jacs.2c04182. 35700232

[ref30] Morimoto M.; Bierschenk S. M.; Xia K. T.; Bergman R. G.; Raymond K. N.; Toste F. D. Advances in supramolecular host-mediated reactivity. Nat. Catal. 2020, 3, 969–984. 10.1038/s41929-020-00528-3.

[ref31] Piskorz T. K.; Martí-Centelles V.; Spicer R. L.; Duarte F.; Lusby P. J. Picking the lock of coordination cage catalysis. Chem. Sci. 2023, 14, 11300–11331. 10.1039/D3SC02586A. 37886081 PMC10599471

[ref32] Curran D. P. Acceleration of a dipolar Claisen rearrangement by Hydrogen bonding to a soluble diarylurea. Tetrahedron Lett. 1995, 36, 6647–6650. 10.1016/00404-0399(50)1394w-.

[ref33] Wittkopp A.; Schreiner P. R. Metal-Free, Noncovalent Catalysis of Diels–Alder Reactions by Neutral Hydrogen Bond Donors in Organic Solvents and in Water. Chem. - Eur. J. 2003, 9, 407–414. 10.1002/chem.200390042. 12532289

[ref34] Uyeda C.; Jacobsen E. N. Enantioselective Claisen Rearrangements with a Hydrogen-Bond Donor Catalyst. J. Am. Chem. Soc. 2008, 130, 9228–9229. 10.1021/ja803370x. 18576616 PMC2547484

[ref35] Zhao Y.; Domoto Y.; Orentas E.; Beuchat C.; Emery D.; Mareda J.; Sakai N.; Matile S. Catalysis with Anion−π Interactions. Angew. Chem., Int. Ed. 2013, 52, 9940–9943. 10.1002/anie.201305356. 23946201

[ref36] Jungbauer S. H.; Walter S. M.; Schindler S.; Rout L.; Kniep F.; Huber S. M. Activation of a carbonyl compound by halogen bonding. Chem. Commun. 2014, 50, 6281–6284. 10.1039/c4cc03124e. 24796408

[ref37] La Manna P.; Talotta C.; Floresta G.; De Rosa M.; Soriente A.; Rescifina A.; Gaeta C.; Neri P. Mild Friedel–Crafts Reactions inside a Hexameric Resorcinarene Capsule: C–Cl Bond Activation through Hydrogen Bonding to Bridging Water Molecules. Angew. Chem., Int. Ed. 2018, 57, 5423–5428. 10.1002/anie.201801642. 29533510

[ref38] Wang K.; Cai X.; Yao W.; Tang D.; Kataria R.; Ashbaugh H. S.; Byers L. D.; Gibb B. C. Electrostatic Control of Macrocyclization Reactions within Nanospaces. J. Am. Chem. Soc. 2019, 141, 6740–6747. 10.1021/jacs.9b02287. 30929421

[ref39] Li T. R.; Huck F.; Piccini G.; Tiefenbacher K. Mimicry of the proton wire mechanism of enzymes inside a supramolecular capsule enables β-selective O-glycosylations. Nat. Chem. 2022, 14, 985–994. 10.1038/s41557-022-00981-6. 35798949

[ref40] Andrews K. G.; Piskorz T. K.; Horton P. N.; Coles S. J. Enzyme-like Acyl Transfer Catalysis in a Bifunctional Organic Cage. J. Am. Chem. Soc. 2024, 146 (26), 17887–17897. 10.1021/jacs.4c03560. 38914009 PMC11228979

[ref41] Rideout D. C.; Breslow R. Hydrophobic acceleration of Diels-Alder reactions. J. Am. Chem. Soc. 1980, 102, 7816–7817. 10.1021/ja00546a048.

[ref42] Mackay L. G.; Wylie R. S.; Sanders J. K. M. Catalytic Acyl Transfer by a Cyclic Porphyrin Trimer: Efficient Turnover without Product Inhibition. J. Am. Chem. Soc. 1994, 116, 3141–3142. 10.1021/ja00086a061.

[ref43] Kang J.; Rebek J. Acceleration of a Diels–Alder reaction by a self-assembled molecular capsule. Nature 1997, 385, 50–52. 10.1038/385050a0. 8985245

[ref44] Mosca S.; Yu Y.; Gavette J. V.; Zhang K.-D.; Rebek J. Jr. A Deep Cavitand Templates Lactam Formation in Water. J. Am. Chem. Soc. 2015, 137, 14582–14585. 10.1021/jacs.5b10028. 26540097

[ref45] Tehrani F. N.; Assaf K. I.; Hein R.; Jensen C. M. E.; Nugent T. C.; Nau W. M. Supramolecular Catalysis of a Catalysis-Resistant Diels–Alder Reaction: Almost Theoretical Acceleration of Cyclopentadiene Dimerization inside Cucurbit[7]uril. ACS Catal. 2022, 12, 2261–2269. 10.1021/acscatal.1c05659.

[ref46] Pluth M. D.; Bergman R. G.; Raymond K. N. Acid catalysis in basic solution: a supramolecular host promotes orthoformate hydrolysis. Science 2007, 316, 85–88. 10.1126/science.1138748. 17412953

[ref47] Hastings C. J.; Pluth M. D.; Bergman R. G.; Raymond K. N. Enzymelike Catalysis of the Nazarov Cyclization by Supramolecular Encapsulation. J. Am. Chem. Soc. 2010, 132, 6938–6940. 10.1021/ja102633e. 20443566

[ref48] Hart-Cooper W. M.; Clary K. N.; Toste F. D.; Bergman R. G.; Raymond K. N. Selective Monoterpene-like Cyclization Reactions Achieved by Water Exclusion from Reactive Intermediates in a Supramolecular Catalyst. J. Am. Chem. Soc. 2012, 134, 17873–17876. 10.1021/ja308254k. 23066637

[ref49] Paul A.; Shipman M. A.; Onabule D. Y.; Sproules S.; Symes M. D. Selective aldehyde reductions in neutral water catalysed by encapsulation in a supramolecular cage. Chem. Sci. 2021, 12, 5082–5090. 10.1039/D1SC00896J. 34163748 PMC8179549

[ref50] Murase T.; Nishijima Y.; Fujita M. Cage-catalyzed Knoevenagel condensation under neutral conditions in water. J. Am. Chem. Soc. 2012, 134, 162–164. 10.1021/ja210068f. 22145970

[ref51] Samanta D.; Mukherjee P. S. Multicomponent self-sorting of a Pd_7_ molecular boat and its use in catalytic Knoevenagel condensation. Chem. Commun. 2013, 49, 4307–4309. 10.1039/c2cc37377g. 23295651

[ref52] Bolliger J. L.; Belenguer A. M.; Nitschke J. R. Enantiopure Water-Soluble [Fe_4_L_6_] Cages: Host–Guest Chemistry and Catalytic Activity. Angew. Chem., Int. Ed. 2013, 52, 7958–7962. 10.1002/anie.201302136. 23788518

[ref53] Wang J.; Young T. A.; Duarte F.; Lusby P. J. Synergistic Noncovalent Catalysis Facilitates Base-Free Michael Addition. J. Am. Chem. Soc. 2020, 142, 17743–17750. 10.1021/jacs.0c08639. 32927950

[ref54] Boaler P. J.; Piskorz T. K.; Bickerton L. E.; Wang J.; Duarte F.; Lloyd-Jones G. C.; Lusby P. J. Origins of High-Activity Cage-Catalyzed Michael Addition. J. Am. Chem. Soc. 2024, 146, 19317–19326. 10.1021/jacs.4c05160. 38976816 PMC11258793

[ref55] DiNardi R. G.; Rasheed S.; Capomolla S. S.; Chak M. H.; Middleton I. A.; Macreadie L. K.; Violi J. P.; Donald W. A.; Lusby P. J.; Beves J. E. Photoswitchable Catalysis by a Self-Assembled Molecular Cage. J. Am. Chem. Soc. 2024, 146, 21196–21202. 10.1021/jacs.4c04846. 39051845 PMC11311219

[ref56] Mozaceanu C.; Taylor C. G. P.; Piper J. R.; Argent S. P.; Ward M. D. Catalysis of an Aldol Condensation Using a Coordination Cage. Chemistry 2020, 2, 22–32. 10.3390/chemistry2010004.

[ref57] Taylor C. G. P.; Metherell A. J.; Argent S. P.; Ashour F. M.; Williams N. H.; Ward M. D. Coordination-Cage-Catalysed Hydrolysis of Organophosphates: Cavity- or Surface-Based?. Chem. - Eur. J. 2020, 26, 3065–3073. 10.1002/chem.201904708. 31774202 PMC7079011

[ref58] Marcos V.; Stephens A. J.; Jaramillo-Garcia J.; Nussbaumer A. L.; Woltering S. L.; Valero A.; Lemonnier J.-F.; Vitorica-Yrezabal I. J.; Leigh D. A. Allosteric initiation and regulation of catalysis with a molecular knot. Science 2016, 352, 1555–1559. 10.1126/science.aaf3673. 27339983

[ref59] August D. P.; Nichol G. S.; Lusby P. J. Maximizing Coordination Capsule–Guest Polar Interactions in Apolar Solvents Reveals Significant Binding. Angew. Chem., Int. Ed. 2016, 55, 15022–15026. 10.1002/anie.201608229. 27809382

[ref60] Martí-Centelles V.; Lawrence A. L.; Lusby P. J. High Activity and Efficient Turnover by a Simple, Self-Assembled “Artificial Diels–Alderase”. J. Am. Chem. Soc. 2018, 140, 2862–2868. 10.1021/jacs.7b12146. 29406705

[ref61] Whitehead D. C.; Yousefi R.; Jaganathan A.; Borhan B. An Organocatalytic Asymmetric Chlorolactonization. J. Am. Chem. Soc. 2010, 132, 3298–3300. 10.1021/ja100502f. 20170118 PMC2883568

[ref62] Denmark S. E.; Ryabchuk P.; Burk M. T.; Gilbert B. B. Toward Catalytic, Enantioselective Chlorolactonization of 1,2-Disubstituted Styrenyl Carboxylic Acids. J. Org. Chem. 2016, 81, 10411–10423. 10.1021/acs.joc.6b01455. 27555101 PMC5100718

[ref63] O’Connor H. M.; Tipping W. J.; Vallejo J.; Nichol G. S.; Faulds K.; Graham D.; Brechin E. K.; Lusby P. J. Utilizing Raman Spectroscopy as a Tool for Solid- and Solution-Phase Analysis of Metalloorganic Cage Host–Guest Complexes. Inorg. Chem. 2023, 62, 1827–1832. 10.1021/acs.inorgchem.2c00873. 35512336 PMC9906719

[ref64] Martí-Centelles V.; Spicer R. L.; Lusby P. J. Non-covalent allosteric regulation of capsule catalysis. Chem. Sci. 2020, 11, 3236–3240. 10.1039/D0SC00341G. 34122830 PMC8157338

[ref65] Yousefi R.; Sarkar A.; Ashtekar K. D.; Whitehead D. C.; Kakeshpour T.; Holmes D.; Reed P.; Jackson J. E.; Borhan B. Mechanistic Insights into the Origin of Stereoselectivity in an Asymmetric Chlorolactonization Catalyzed by (DHQD)2PHAL. J. Am. Chem. Soc. 2020, 142, 7179–7189. 10.1021/jacs.0c01830. 32202109

[ref66] Young T. A.; Martí-Centelles V.; Wang J.; Lusby P. J.; Duarte F. Rationalizing the Activity of an “Artificial Diels-Alderase”: Establishing Efficient and Accurate Protocols for Calculating Supramolecular Catalysis. J. Am. Chem. Soc. 2020, 142, 1300–1310. 10.1021/jacs.9b10302. 31852191

