# Supplementary material for: Dissecting the Effects of Cage Structure in the Catalytic Activation of Imide Chlorenium-Ion Donors
Source: J Am Chem Soc. 2025 Mar 21;147(13):11456–64. doi: 10.1021/jacs.5c01249 (PMC11969530; doi:10.1021/jacs.5c01249)
Supplement: Supplementary file 1 — ja5c01249_si_001.pdf [file ja5c01249_si_001.pdf]

# Supporting Information

## Dissecting the Effects of Cage Structure in the Catalytic Activation of Imide Chlorenium Ion Donors

Hang Zhou,[a] Tomasz K. Piskorz,[b] Keyu Liu,[a] Yining Lu,[b] Fernanda Duarte[b]* and Paul J. Lusby[a]*

[a]EaStCHEM School of Chemistry, University of Edinburgh, Joseph Black Building, David Brewster Road, Edinburgh, Scotland, EH9 3FJ, U.K.

[b]Chemistry Research Laboratory, University of Oxford, Oxford OX1 3TA, U.K.

# Contents


1. General information ........ 1
1.1 Materials and methods ........ 1
1.2 Cage Synthesis ........ 1
1.3 Substrate Synthesis ........ 1
2. Chlorocyclization reactions monitoring ........ 1
2.1 General procedure for monitoring chlorocyclization reactions using $^{1}$H NMR Spectroscopy 1
2.2 Kinetic profiles and $^{1}$H NMR spectra of chlorocyclization ........ 2
2.3.1 Chlorolactonization between **1a** and DCDMH ........ 2
2.3.2 Chlorocycloetherification between **1b** and DCDMH ........ 3
2.3.3 Chlorocycloetherification between **1b** and NCS ........ 3
2.3.4 Chlorolactonization between **1c** and DCDMH ........ 5
2.3.5 Chlorocycloetherification between **1d** and DCDMH ........ 6
2.3.6 Kinetic profiles of chlorocyclization between **1** and DCDHM ........ 7
2.3.7 Chlorolactonization between **3a** and DCDMH ........ 7
2.3.8 Chlorocycloetherification between **3b** and DCDMH ........ 8
2.3.9 Chlorolactonization between **3c** and DCDMH ........ 8
2.3.10 Chlorocycloetherification between **3d** and DCDMH ........ 8
2.3.11 Kinetic profiles of chlorocyclization between **3** and DCDHM ........ 9
2.3 Control reactions for the chlorocycloetherification of **1b** with NCS ........ 10
2.4.1 Table for control reactions ........ 10
2.4.2 Kinetic profiles and $^{1}$H NMR spectra of control reactions with **C1** ........ 10
2.4.3 Kinetic profiles and $^{1}$H NMR spectra of control reactions with **C3** ........ 12
3. Host-guest chemistry via NMR titration ........ 14
3.1 General procedure ........ 14
3.2 Table of the association constant ........ 14
3.3 Titration of NCS into **C1** ........ 15
3.4 Titration of NCS into **C3** ........ 16
3.5 Titration of Triphenylphosphine oxide into **C3** ........ 17
4. Michaelis-Menten analysis ........ 18
4.1 General procedure ........ 18
4.1.1 Michaelis-Menten kinetics ........ 18
4.1.2 Cage catalyzed reaction ........ 18
4.1.3 Uncatalyzed reaction ........ 18
4.2 Kinetic profiles and $^{1}$H NMR spectra of **1b** using variable [NCS] ........ 19
4.2.1 Kinetic profiles and $^{1}$H NMR spectra for the uncatalyzed reaction ........ 19

4.2.2 Kinetic profiles and $^{1}$H NMR spectra with **C1** using variable [NCS] .......... 20
4.2.3 Kinetic profiles and $^{1}$H NMR spectra with **C3** using variable [NCS] .......... 24
4.3 Table of Michaelis-Menten constants .......... 29
5. Chlorination between **5** and NCS .......... 30
5.1 General procedure .......... 30
5.2 NMR scale reaction yields .......... 30
5.3 Kinetic profiles and $^{1}$H NMR spectra of **5** and NCS .......... 31
5.4 Table of the kinetic constants for chlorination of 5. .......... 33
6. Computational methods .......... 34
6.1 Method .......... 34
6.2 Chlorocycloetherification .......... 35
6.2.1 Cage Binding .......... 35
6.2.2 Uncatalyzed reaction .......... 38
6.2.3 Catalyzed reaction .......... 40
6.3 α-methylstyrene chlorination .......... 42
6.3.1 Uncatalyzed reaction .......... 42
6.3.2 Catalyzed reaction .......... 44
6.3.3 Fragment energy decomposition analysis .......... 46
7. Reference .......... 49

# 1. General information

## 1.1 Materials and methods

Unless otherwise stated, all reagents and solvents were purchased from Alfa Aesar, VWR, Fluorochem or Sigma Aldrich and used without further purification. Column chromatography was carried out using Geduran Si60 (40-63 μm) as the stationary phase and TLC was performed on precoated Kieselgel 60 plates (0.20 mm thick, 60F254. Merck, Germany) and observed under UV light at 254 nm or 365 nm. All reactions were carried out under air and at room temperature, unless otherwise stated.

All $^{1}H$ and $^{13}C$ NMR spectra were recorded on either a 500 MHz Bruker AV III equipped with a DCH ATMA cryo-probe (Ava500), a 400MHZ Bruker AV III equipped with BBFO+ ATMA probe (Ava400), a 500 MHz Bruker AV IIIHD equipped with a Prodigy cryo-probe (Pro500) or a 600 MHz Bruker AV I equipped with a BBO probe (Ava600) at a constant temperature of 300 K. Chemical shifts are reported in parts per million. Coupling constants (J) are reported in hertz (Hz). Standard abbreviations indicating multiplicity were used as follows: m = multiplet, q = quartet, t = triplet, d = doublet, s = singlet. All analysis was performed with MestReNova, Version 14.0.0. All assignments were confirmed using a combination of COSY, NOESY, HMBC and HSQC NMR spectra. succinimide

## 1.2 Cage Synthesis

Cage **C1**, **C2** and **C3**, and $[Pd(py)_4](BArF)_2$ were synthesized according to literature procedures.[S1,S2,S3]

## 1.3 Substrate Synthesis

**1a-d** and **3a-c** were synthesized according to literature procedures.[S4,S5,S6,S7,S8,S9,S10]

# 2. Chlorocyclization reactions monitoring

## 2.1 General procedure for monitoring chlorocyclization reactions using $^{1}H$ NMR Spectroscopy

Prior to NMR scale reactions, NCS and DCDMH were recrystallized from $CH_3CN$ and their stock solutions were filtered through $K_2CO_3$. $^{1}H$ NMR spectra were recorded either at the end of the reaction, or periodically to obtain yields and kinetic data, using integration of the substrate / product against the standard. Product peaks were assigned using a combination of NMR techniques and by comparison to literature data.[S11,S12,S13,S14,S15,S16]

Uncatalyzed reactions: To an NMR tube was introduced a solution containing the alkene substrate (24 μL of a 200 mM $CD_2Cl_2$ stock solution), the chloro-reagent (48 μL of a 200 mM $CD_2Cl_2$ stock solution), the internal standard tetrakis(trimethylsilyl)silane (30 μL of a 20 mM $CD_2Cl_2$ stock solution), and $CD_2Cl_2$ (378 μL).

Catalyzed reactions with the cage: To an NMR tube was introduced the cage compound (0.00048 mmol weighed in the tube), the alkene substrate (24 μL of a 200 mM $CD_2Cl_2$ stock solution), the chloro-reagent (48 μL of a 200 mM $CD_2Cl_2$ stock solution), the internal standard tetrakis(trimethylsilyl)silane (30 μL of a 20 mM $CD_2Cl_2$ stock solution), and $CD_2Cl_2$ (378 μL).

For catalyzed reactions with the cage and internal inhibitor: To an NMR tube was introduced the cage compound (0.00048 mmol weighing in the tube), 9,10-dicyanoanthracene (DCA) or 6,13-pentancenequinone (PQ) (0.00048 mmol weighing in the tube) the alkene substrate (24 µL of a 200 mM $CD_2Cl_2$ stock solution), the chloro-reagent (48 µL of a 200 mM $CD_2Cl_2$ stock solution), the internal standard tetrakis(trimethylsilyl)silane (30 µL of a 20 mM $CD_2Cl_2$ stock solution), and $CD_2Cl_2$ (378 µL).

For catalyzed reactions with the cage and triphenylphosphine oxide external inhibitor: To an NMR tube was introduced the cage compound (0.00048 mmol weighing in the tube), triphenylphosphine oxide (48 µL of a 20 mM $CD_2Cl_2$ stock solution) the alkene substrate (24 µL of a 200 mM $CD_2Cl_2$ stock solution), the chloro-reagent (48 µL of a 200 mM $CD_2Cl_2$ stock solution), the internal standard tetrakis(trimethylsilyl)silane (30 µL of a 20 mM $CD_2Cl_2$ stock solution), and $CD_2Cl_2$ (330 µL).

For reactions with $[Pd(py)_4](BArF)_2$: To an NMR tube was introduced a solution containing $[Pd(py)_4](BArF)_2$ (48 µL of a 20 mM $CD_2Cl_2$ stock solution), the alkene substrate (24 µL of a 200 mM $CD_2Cl_2$ stock solution), the chloro-reagent (48 µL of a 200 mM $CD_2Cl_2$ stock solution), the internal standard tetrakis(trimethylsilyl)silane (30 µL of a 20 mM $CD_2Cl_2$ stock solution), and $CD_2Cl_2$ (330 µL).

## 2.2 Kinetic profiles and $^1$H NMR spectra of chlorocyclization

### 2.3.1 Chlorolactonization between **1a** and DCDMH

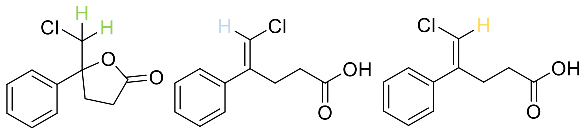


A B C

*Figure S1. Structures of products in reactions of **1a** and DCDMH. A is the major product and B, C are side products.*

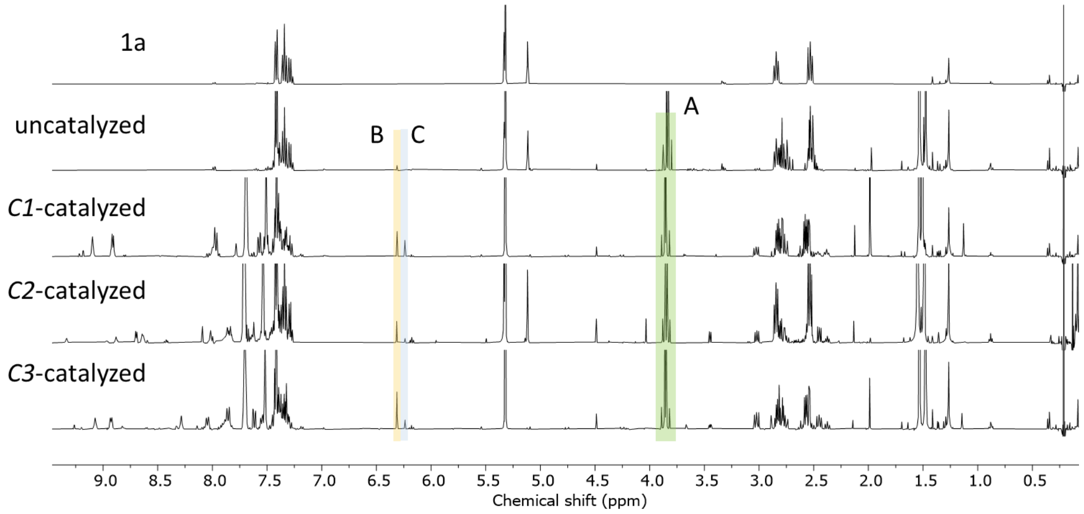


*Figure S2. $^1$H NMR spectra (400 MHz, $CD_2Cl_2$) for the reaction of **1a** (10 mM) and DCDMH (20 mM) with cages (1 mM) for 1.5 h.*

## 2.3.2 Chlorocycloetherification between **1b** and DCDMH

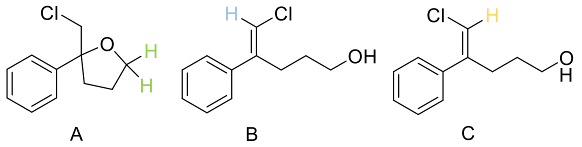


*Figure S3. Structures of products in reactions of **1b** and DCDMH. A is the major product and B, C are side products.*

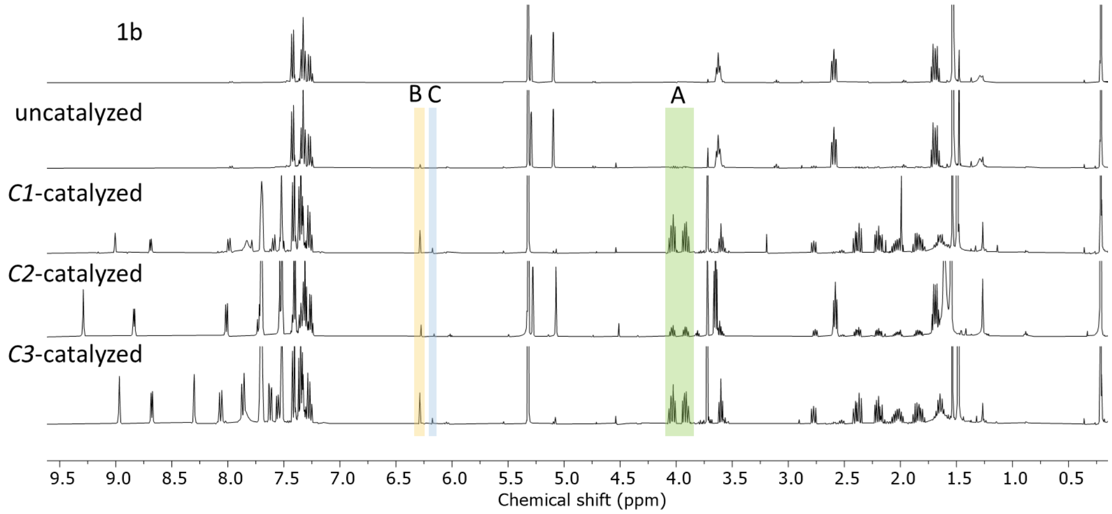


*Figure S4. $^{1}$H NMR spectra (400 MHz, $CD_2Cl_2$) for the reaction of **1b** (10 mM) and DCDMH (20 mM) with cages (1 mM) for 1.5 h.*

## 2.3.3 Chlorocycloetherification between **1b** and NCS

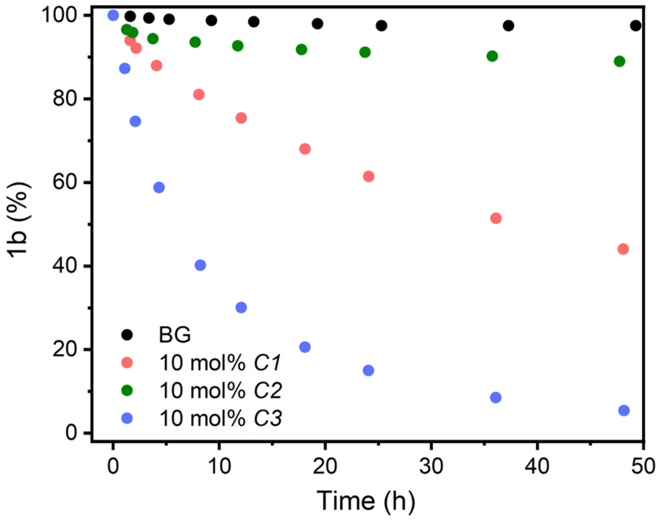


*Figure S5. Kinetic profiles of chlorocycloetherification between 10 mM **1b** and 20 mM NCS. Colour code: background (uncatalyzed) reaction, black; **C1**, red; **C2**, green; **C3**, blue. Cage concentrations are 1 mM.*

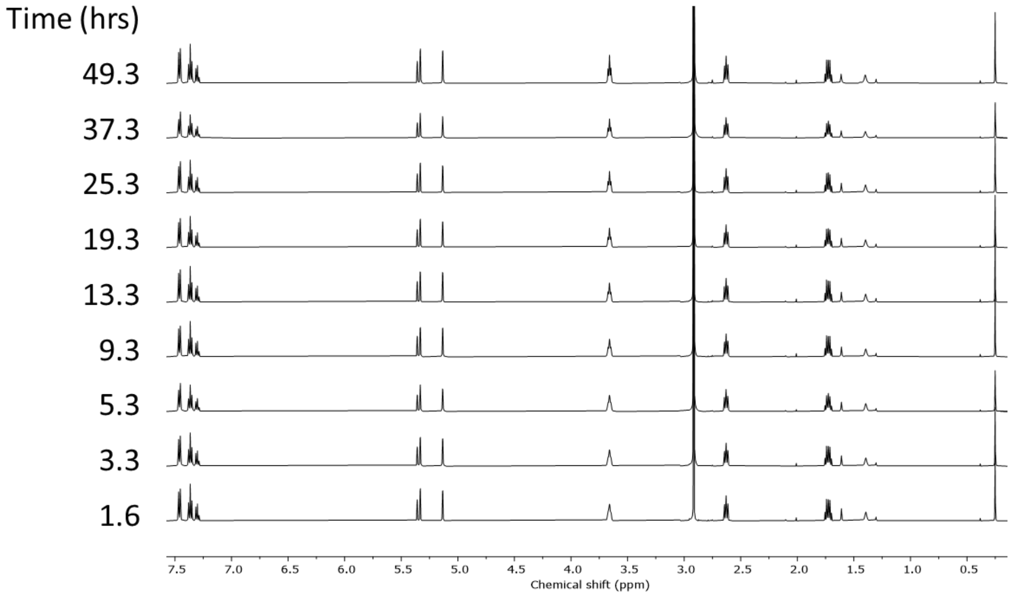


*Figure S6.* *$^{1}$H NMR spectra (600 MHz, $CD_2Cl_2$) for the reaction of* ***1b*** *(10 mM) and NCS (20 mM). We estimate that the yield of* ***2b*** *is < 1%.*

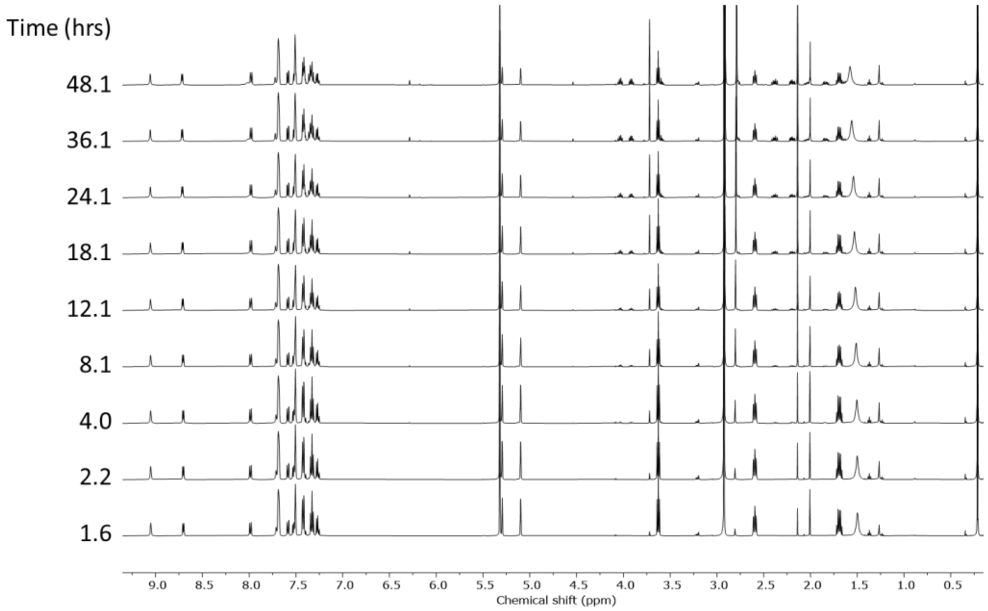


*Figure S7.* *$^{1}$H NMR spectra (600 MHz, $CD_2Cl_2$) for the reaction of* ***1b*** *(10 mM) and NCS (20 mM) with* ***C1*** *(1 mM). NMR integration at 48 h shows product* ***2b*** *is formed in 41% yield (56% conversion).*

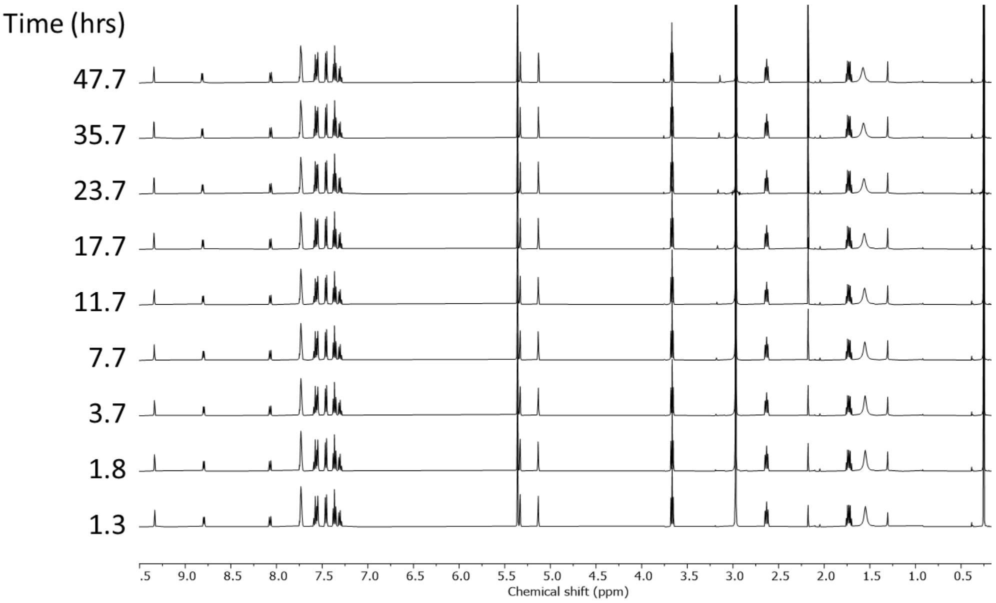


*Figure S8. $^{1}$H NMR spectra (600 MHz, $CD_2Cl_2$) for the reaction of **1b** (10 mM) and NCS (20 mM) with **C2** (1 mM). NMR integration at 48 h shows product **2b** is formed in 3% yield (11% conversion).*

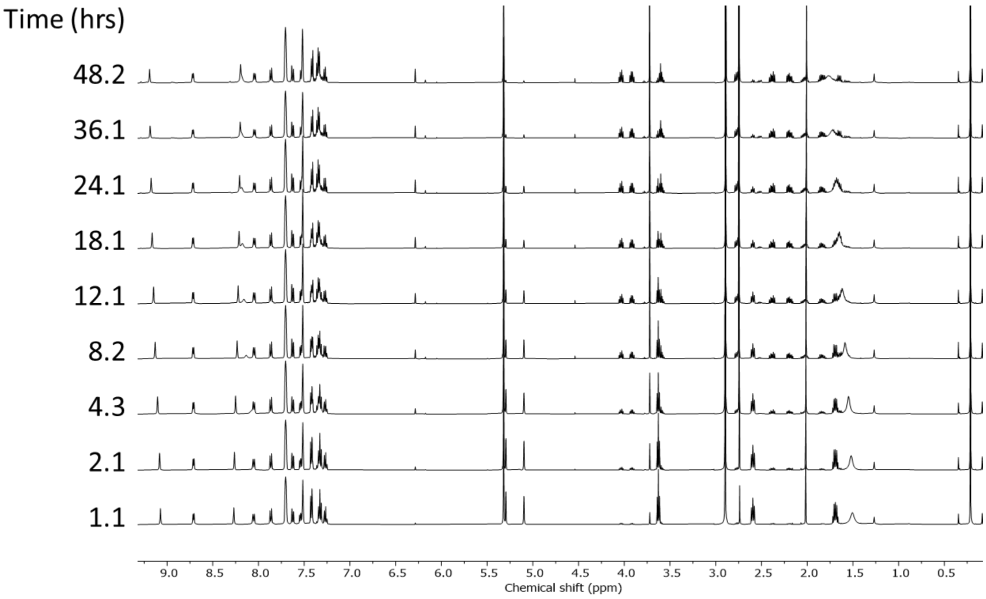


*Figure S9. $^{1}$H NMR spectra (600 MHz, $CD_2Cl_2$) for the reaction of **1b** (10 mM) and NCS (20 mM) with **C3** (1 mM). NMR integration at 48 h shows product **2b** is formed in 63% yield (95% conversion).*

## 2.3.4 Chlorolactonization between **1c** and DCDMH

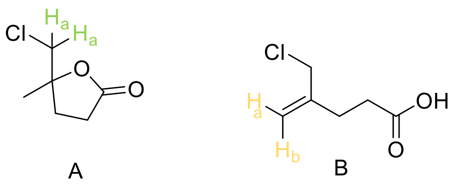


*Figure S10. Structures of products in reactions of **1c** and DCDMH. A is the major product and B is the side product.*

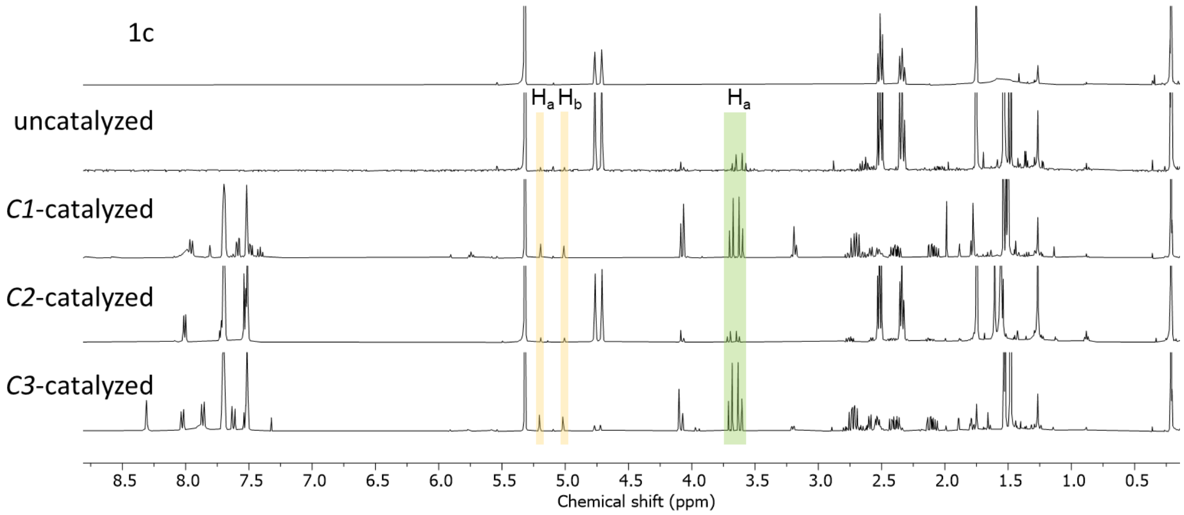


*Figure S11.* *$^{1}$H NMR spectra (400 MHz, $CD_2Cl_2$) for the reaction of **1c** (10 mM) and DCDMH (20 mM) with cages (1 mM) for 1.5 h.*

## 2.3.5 Chlorocycloetherification between **1d** and DCDMH

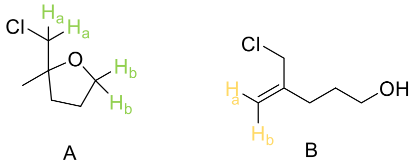


*Figure S12. Structures of products in reactions of **1d** and DCDMH. A is the major product and B is the side product.*

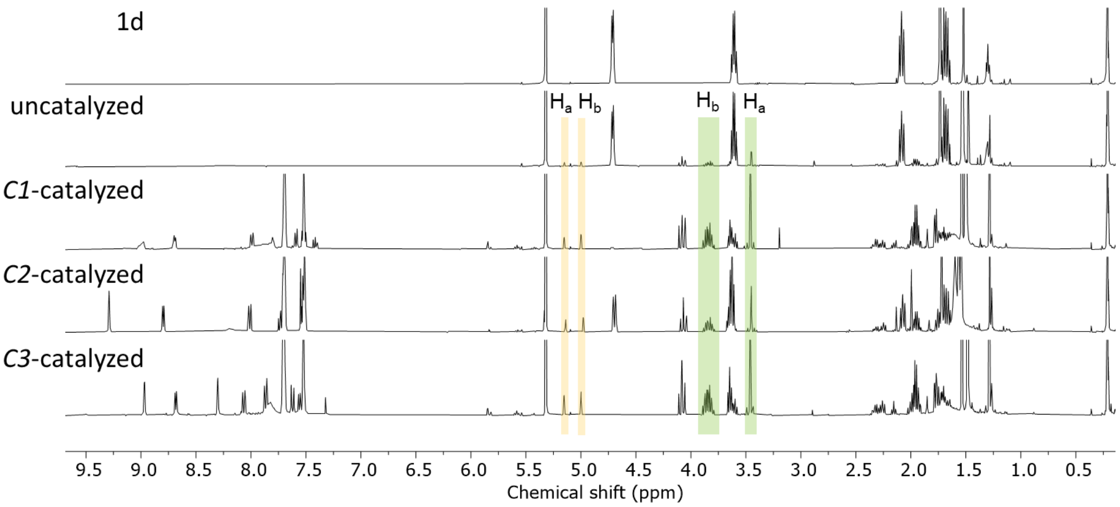


*Figure S13.* *$^{1}$H NMR spectra (400 MHz, $CD_2Cl_2$) for the reaction of **1d** (10 mM) and DCDMH (20 mM) with cages (1 mM) for 1.5 h.*

## 2.3.6 Kinetic profiles of chlorocyclization between **1** and DCDHM

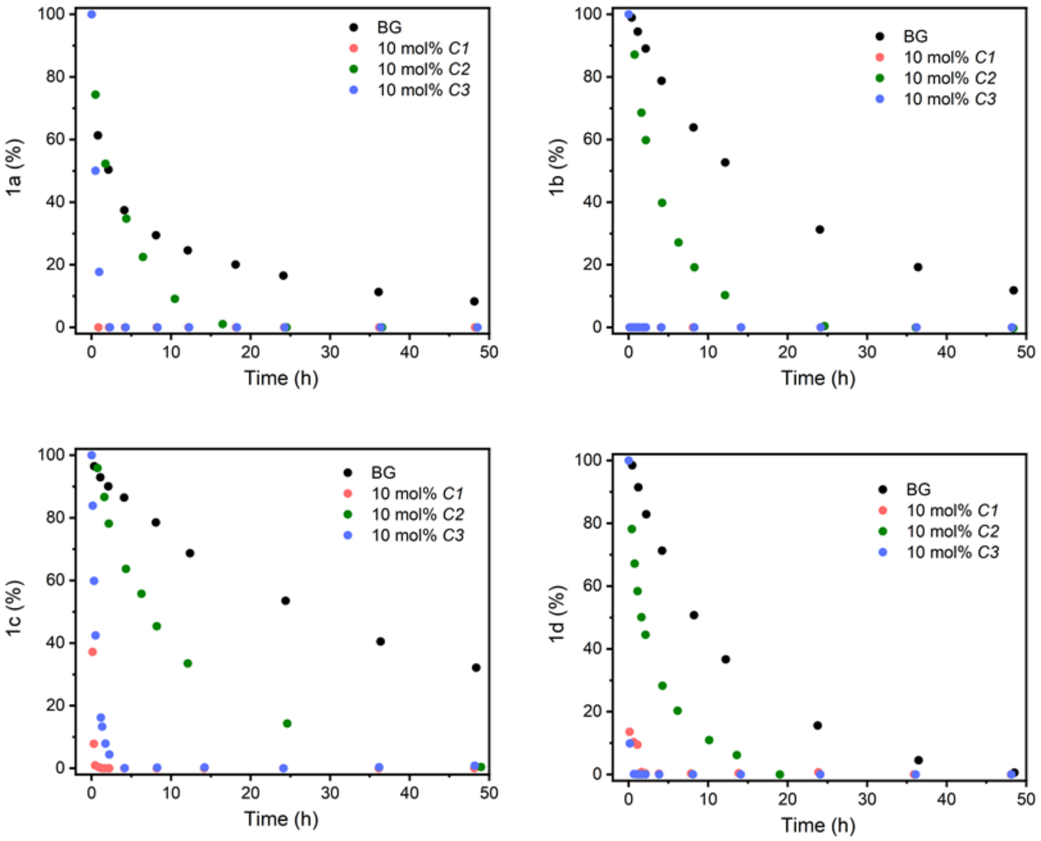


*Figure S14. Kinetic profiles of chlorocyclization between 10 mM **1** and 20 mM DCDMH. Colour code: background (uncatalyzed) reaction, black; **C1**, red; **C2**, green; **C3**, blue. Cage concentrations are 1 mM.*

## 2.3.7 Chlorolactonization between **3a** and DCDMH

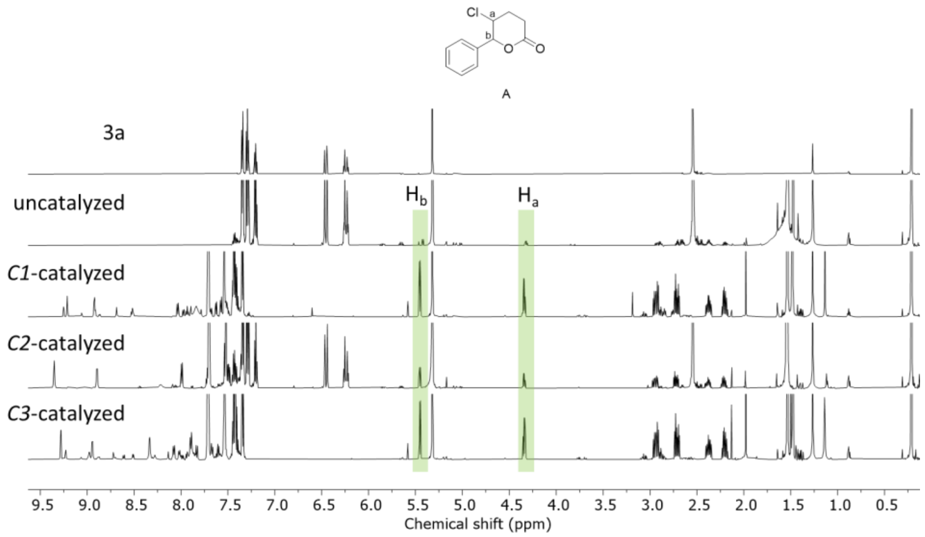


*Figure S15. $^{1}$H NMR spectra (400 MHz, $CD_2Cl_2$) for the reaction of **3a** (10 mM) and DCDMH (20 mM) with cages (1 mM) after 48 h.*

## 2.3.8 Chlorocycloetherification between **3b** and DCDMH

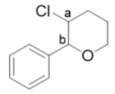

A

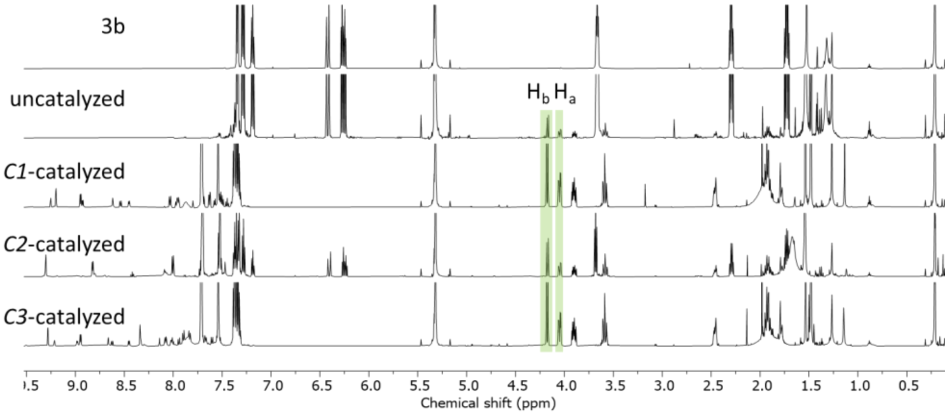


*Figure S16. $^{1}$H NMR spectra (400 MHz, $CD_2Cl_2$) for the reaction of **3b** (10 mM) and DCDMH (20 mM) with cages (1 mM) for 48 h. days.*

## 2.3.9 Chlorolactonization between **3c** and DCDMH

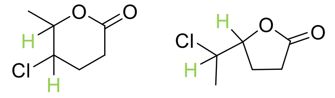

A B

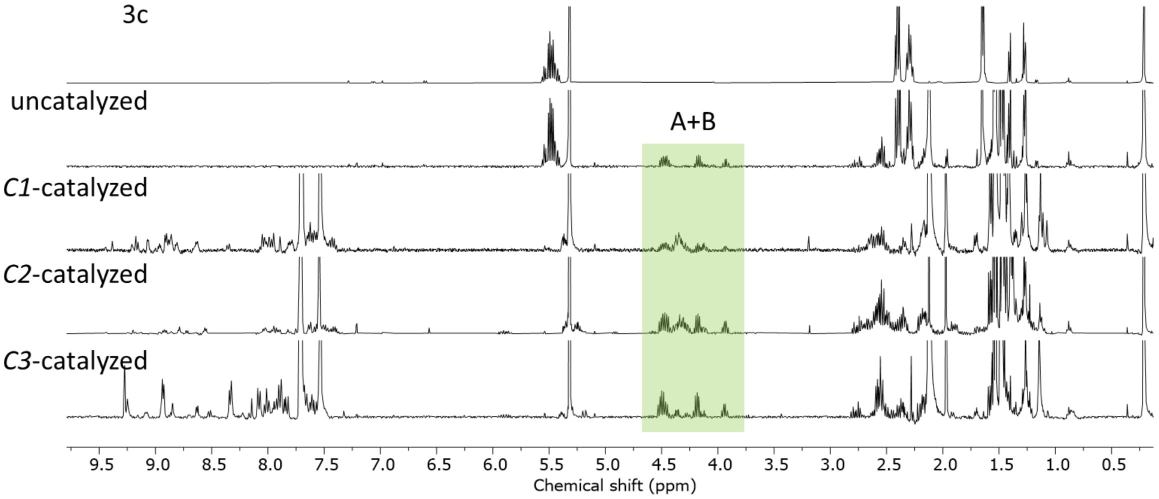


*Figure S17. $^{1}$H NMR spectra (400 MHz, $CD_2Cl_2$) for the reaction of **3c** (10 mM) and DCDMH (20 mM) with cages (1 mM) after 48 h.*

## 2.3.10 Chlorocycloetherification between **3d** and DCDMH

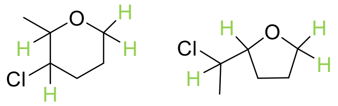

A B

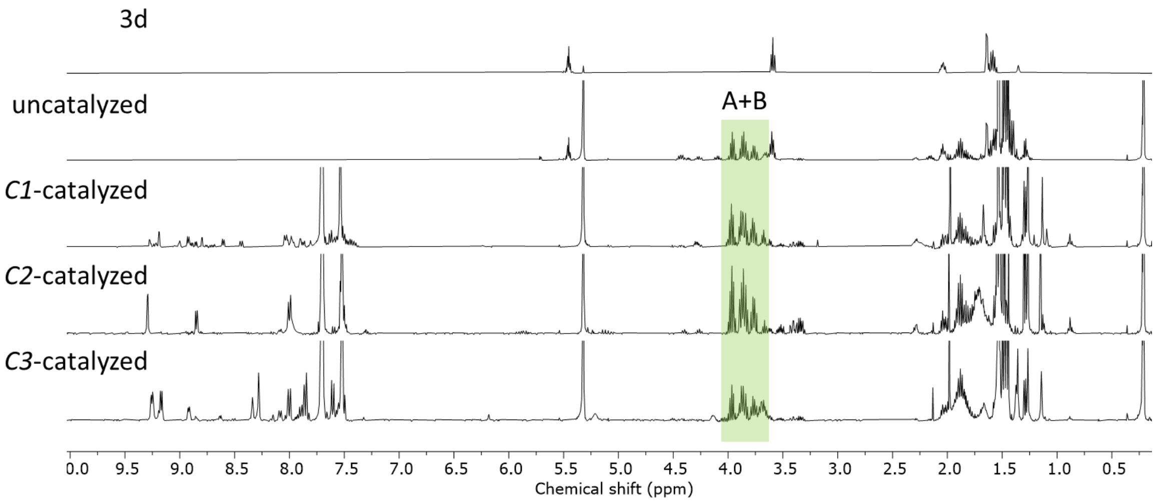


*Figure S18.* [1]*H NMR spectra (400 MHz,* $CD_2Cl_2$*) for the reaction of* ***3d*** *(10 mM) and DCDMH (20 mM) with cages (1 mM) after 48 h.*

## 2.3.11 Kinetic profiles of chlorocyclization between **3** and DCDHM

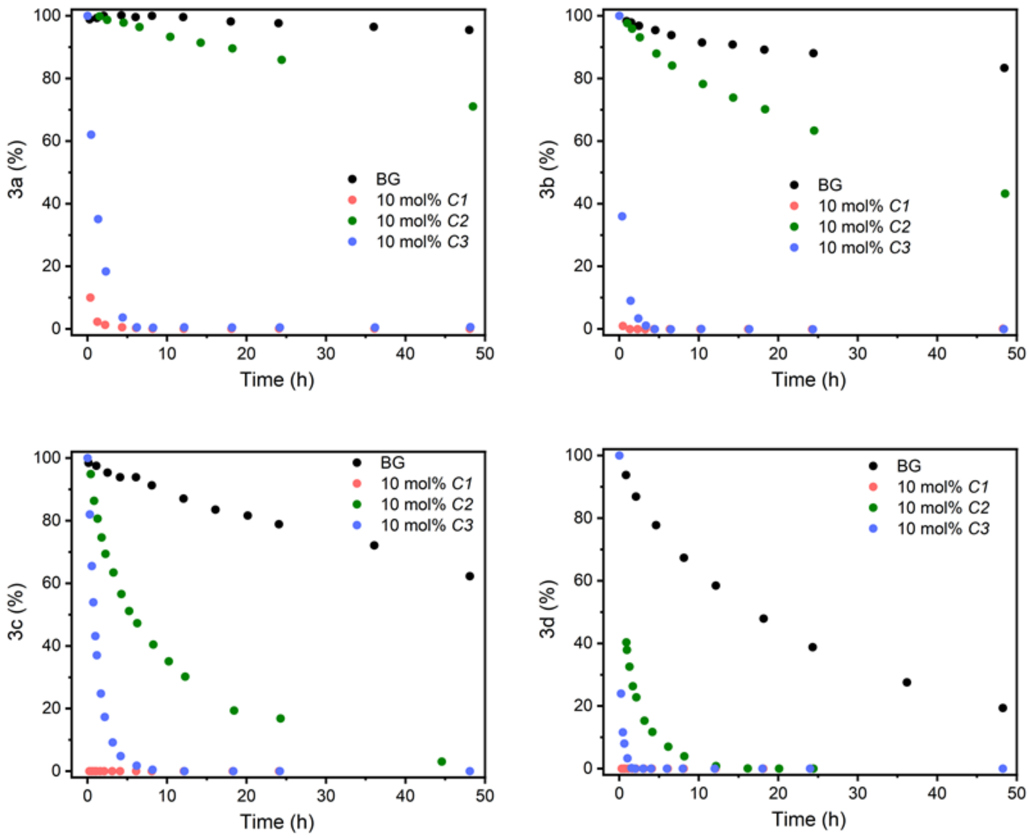


*Figure S19. Kinetic profiles of chlorocyclization between 10 mM* ***3*** *and 20 mM DCDMH. Colour code: background (uncatalyzed) reaction, black;* ***C1****, red;* ***C2****, green;* ***C3****, blue. Cage concentrations are 1 mM.*

## 2.3 Control reactions for the chlorocycloetherification of **1b** with NCS

### 2.4.1 Table for control reactions

*Table S1. Control reaction between **1b** (10 mM) and NCS (20 mM) with **C1** and **C3** (1 mM).*

| Entry | Catalysts | Additive | Yield (conv.) after 48 h |
|---|---|---|---|
| **1** | **C1** | 6,13-pentancenequinone (PQ) | 0% (0%) |
| **2** | **C1** | Triphenylphosphine oxide | 48% (34%) |
| **3** | **C3** | 9,10-dicyanoanthracene (DCA) | 0% (0%) |
| **4** | **C3** | Triphenylphosphine oxide | 64% (92%) |
| **5** | $Pd(py)_4(BArF)_2$ | - | 0% (0%) |

### 2.4.2 Kinetic profiles and $^{1}$H NMR spectra of control reactions with **C1**

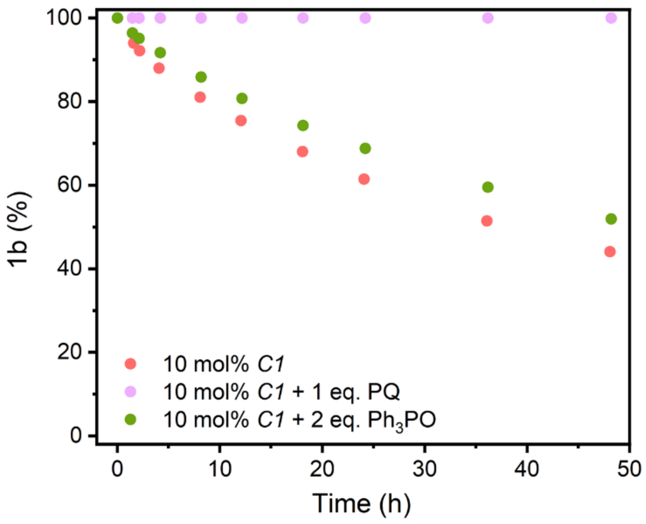


*Figure S20. Kinetic profiles of control reactions between 10 mM **1b** and 20 mM NCS with: 1 mM **C1** (red circle); 1 mM **C1** and 1 mM 6,13-pentancenequinone (PQ) (purple circle); 1 mM **C1** and 2 mM triphenylphosphine oxide (green circle).*

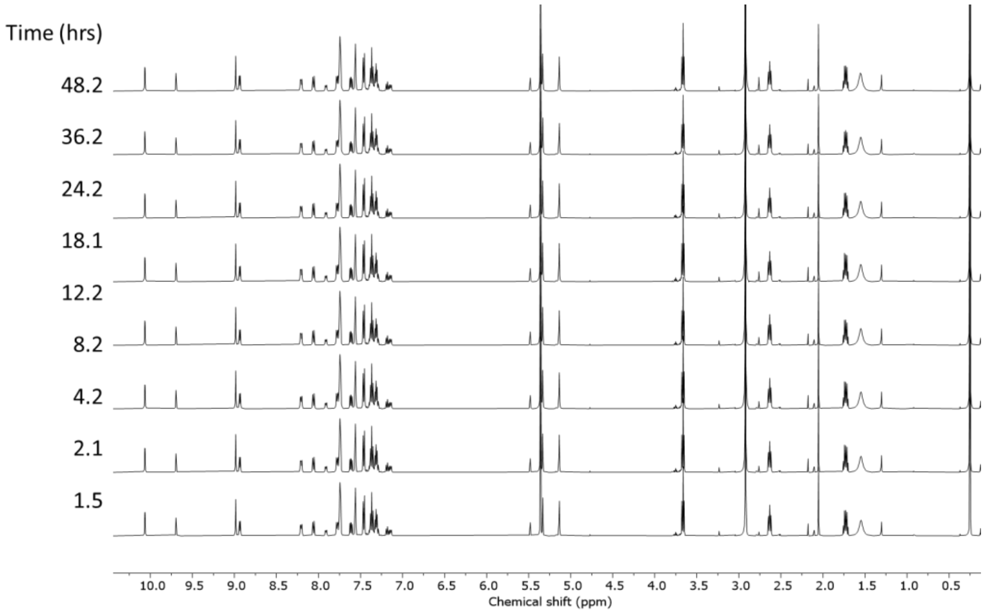


*Figure S21. $^{1}$H NMR spectra (600 MHz, $CD_2Cl_2$) for the reaction of **1b** (10 mM) and NCS (20 mM) with **C1** (1 mM) and 6,13-pentacenequinone (1 mM).*

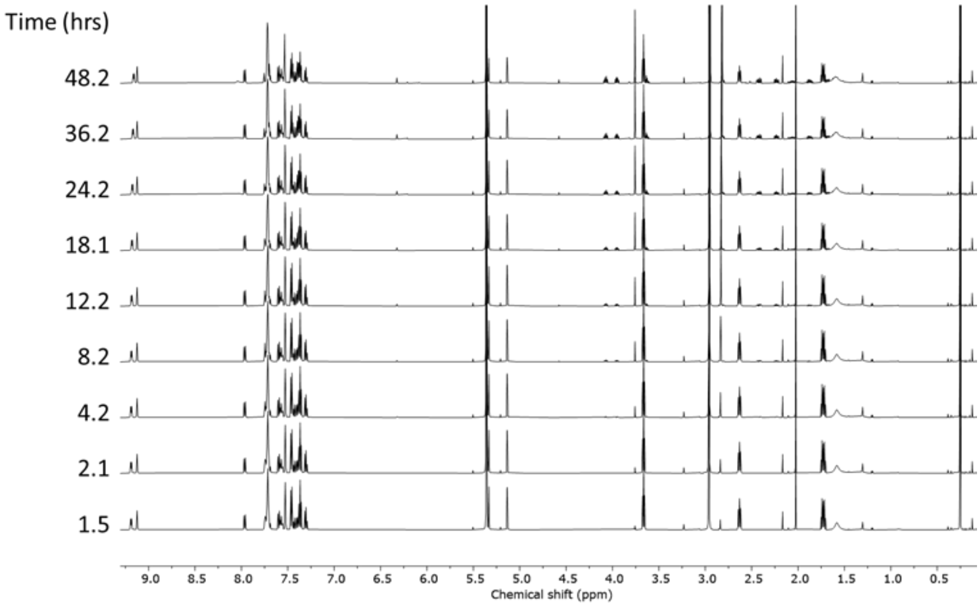


*Figure S22. $^{1}$H NMR spectra (600 MHz, $CD_2Cl_2$) for the reaction of **1b** (10 mM) and NCS (20 mM) with **C1** (1 mM) and Triphenylphosphine oxide (2 mM).*

## 2.4.3 Kinetic profiles and $^{1}$H NMR spectra of control reactions with **C3**

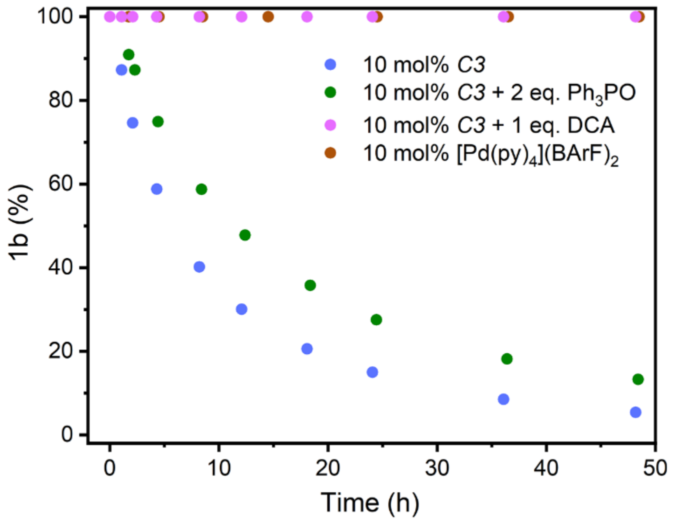


*Figure S23. Kinetic profiles of control reactions between 10 mM **1b** and 20 mM NCS with: 1 mM **C3** (blue circle); 1 mM **C3** and 1 mM 9,10-dicyanoanthracene (DCA) (purple circle); 1 mM **C3** and 2 mM triphenylphosphine oxide (green circle); 2 mM Pd(py)$_4$(BArF)$_2$ (brown circle).*

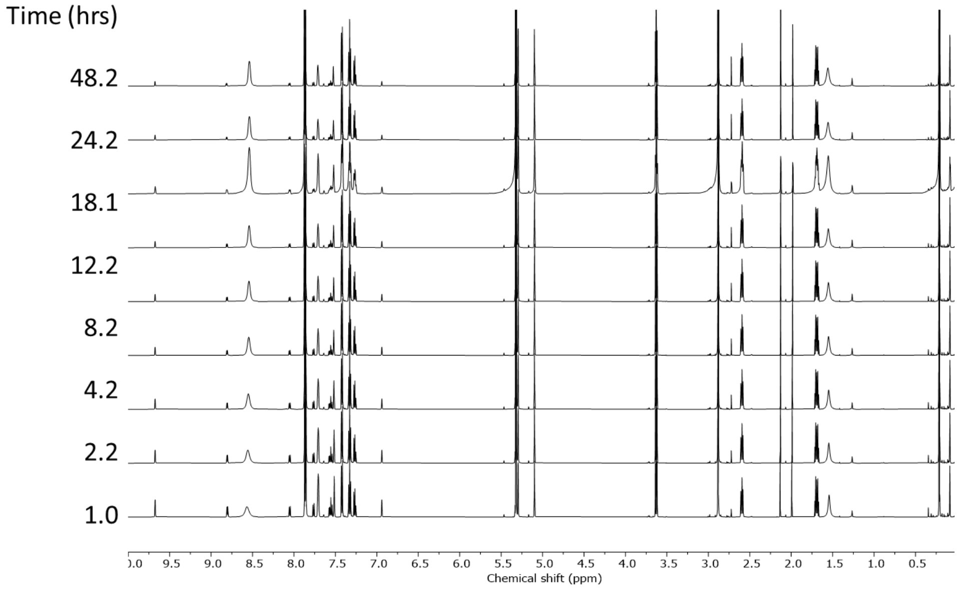


*Figure S24. $^{1}$H NMR spectra (600 MHz, $CD_2Cl_2$) for the reaction of **1b** (10 mM) and NCS (20 mM) with **C3** (1 mM) and 9,10-dicyanoanthracene (DCA) (1 mM).*

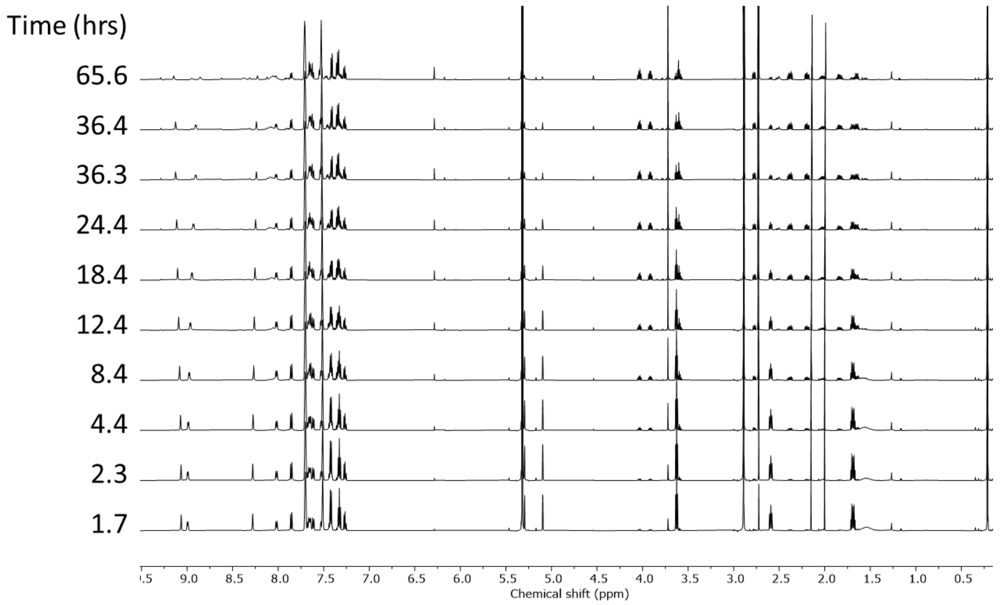


*Figure S25. $^{1}$H NMR spectra (600 MHz, $CD_2Cl_2$) for the reaction of **1b** (10 mM) and NCS (20 mM) with **C3** (1 mM) and triphenylphosphine oxide (2 mM).*

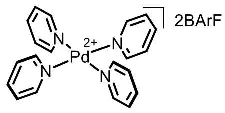


$[Pd(py)_2](BArF)_2$

*Figure S26. Structures of other $[Pd(py)_2](BArF)_2$.*

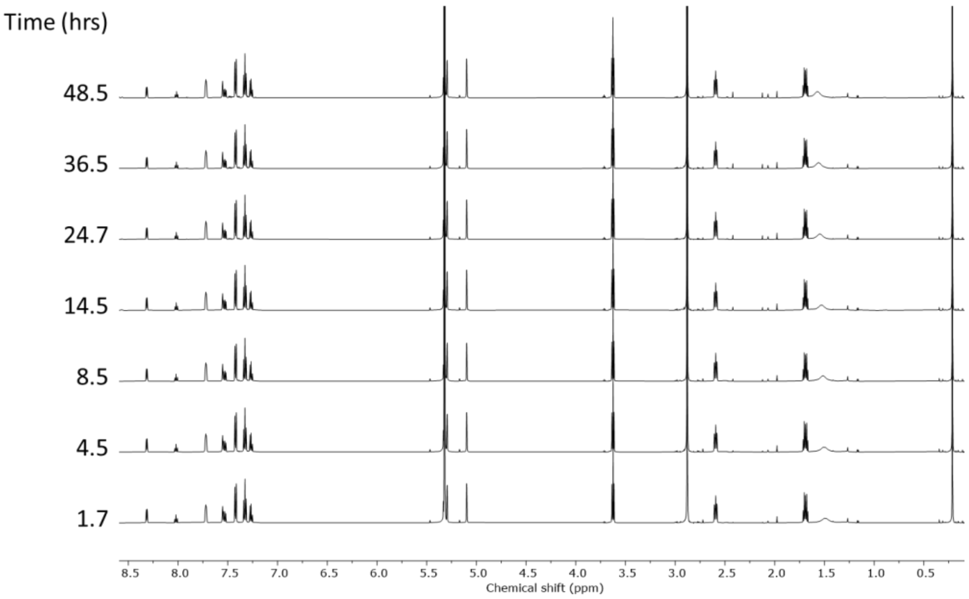


*Figure S27. $^{1}$H NMR spectra (600 MHz, $CD_2Cl_2$) for the reaction of **1b** (10 mM) and NCS (20 mM) with $[Pd(py)_4](BArF)_2$ (2 mM).*

# 3. Host-guest chemistry via NMR titration

## 3.1 General procedure

For each titration, a solution containing the guest (10 mM or 200 mM) and the internal standard of 20mM was titrated to a 500 µL solution containing the cage (2 mg) with the internal standard of 20mM. $^{1}$H NMR spectrum was recorded at each titration point. The peak positions of the internal pyridyl hydrogens were plotted against the concentration of guests. The experimental data was fitted to a 1:1 fast exchange binding model using the Levenberg-Marquardt Nonlinear Least-Squares Algorithm built in the Origin software.[S1]

$$\delta = \delta_0 + \frac{\Delta\delta_{Max}}{2}\left(\frac{C_{Guest}}{C_{Host}} + \frac{1}{C_{Host}\cdot k_{Ass}} + 1 - \sqrt{\left(\frac{C_{Guest}}{C_{Host}} + \frac{1}{C_{Host}\cdot k_{Ass}} + 1\right)^2 - \frac{4\cdot C_{Guest}}{C_{Host}}}\right)$$

*Equation S1*

$$C_{Guest} = \frac{G_0 \Delta v}{v_0 + \Delta v}$$

*Equation S2*

$$C_{Host} = \frac{H_0 v_0}{v_0 + \Delta v}$$

*Equation S3*

Substituting equation S2 and S3 into S1, equation S4 is obtained and used to fit the titration data.

$$\Delta\delta = \frac{\Delta\delta_{Max}}{2}\left(\frac{G_0\Delta v}{H_0 v_0} + \frac{v_0 + \Delta v}{H_0 v_0 k_{Ass}} + 1 - \sqrt{\left(\frac{G_0\Delta v}{H_0 v_0} + \frac{v_0 + \Delta v}{H_0 v_0 k_{Ass}} + 1\right)^2 - \frac{4G_0\Delta v}{H_0 v_0}}\right)$$

*Equation S4*

where $H_0$ is the initial concentration of the host (cage), $G_0$ is the concentration of the stock solution of the guest (NCS), $v_0$ is the initial volume before the titration, $\Delta v$ is the varying volume, $\Delta\delta$ is the change in the chemical shift.

## 3.2 Table of the association constant

*Table S2. Association constants of NCS and inhibitors in **C1** and **C2**.*

| Guest⊂C1 | $k_{Ass}$ ($M^{-1}$) | Guest⊂C3 | $k_{Ass}$ ($M^{-1}$) |
|---|---|---|---|
| NCS | 3.1 × 10$^4$ | NCS | 32 |
| 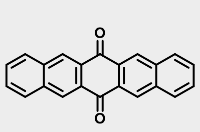 6,13-pentancenequinone[S17] | 8 × 10$^8$ | 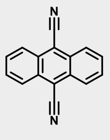 9,10-dicyanoanthracene[S3] | 3.7 × 10$^4$ |
| 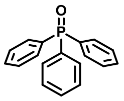 triphenylphosphine oxide[S17] | 2100 | 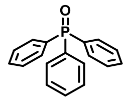 triphenylphosphine oxide | 394 |

## 3.3 Titration of NCS into **C1**

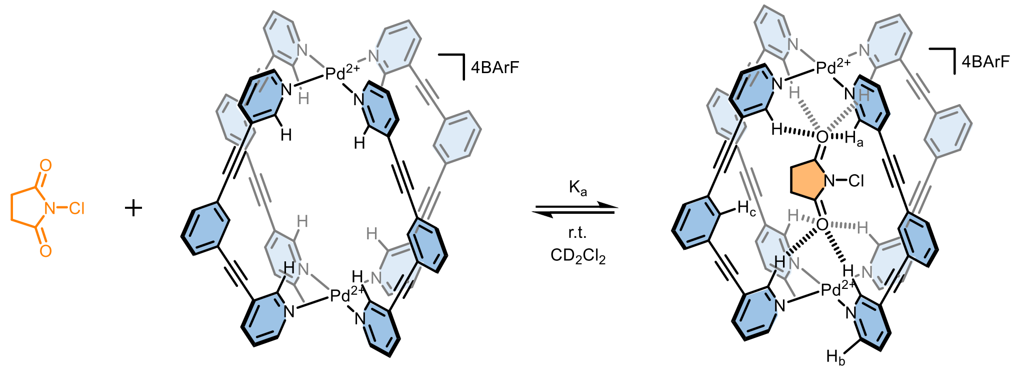


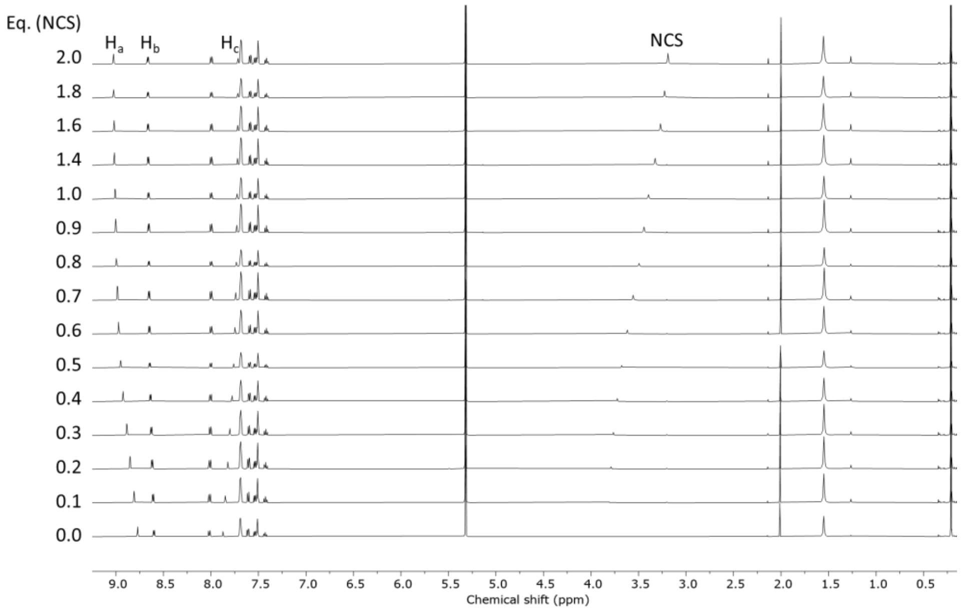


*Figure S28. $^{1}$H NMR (400 MHz, $CD_2Cl_2$) spectroscopic data for titration of NCS into **C1**.*

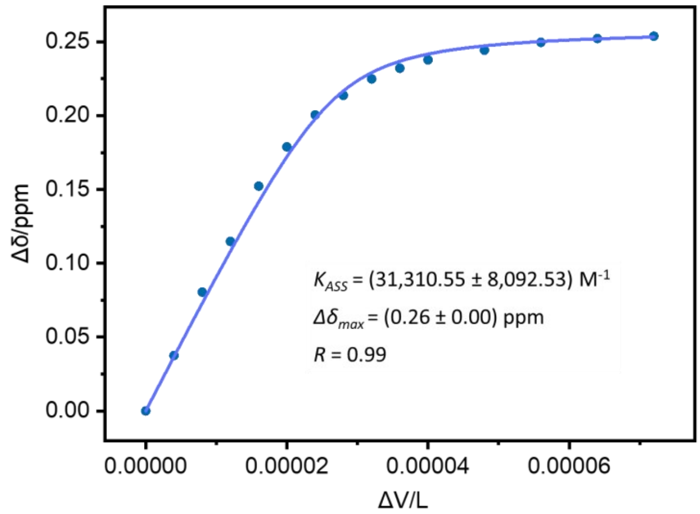


*Figure S29. Fitted data for change in internal peak position ($H_a$) with increasing NCS concentration.*

## 3.4 Titration of NCS into **C3**

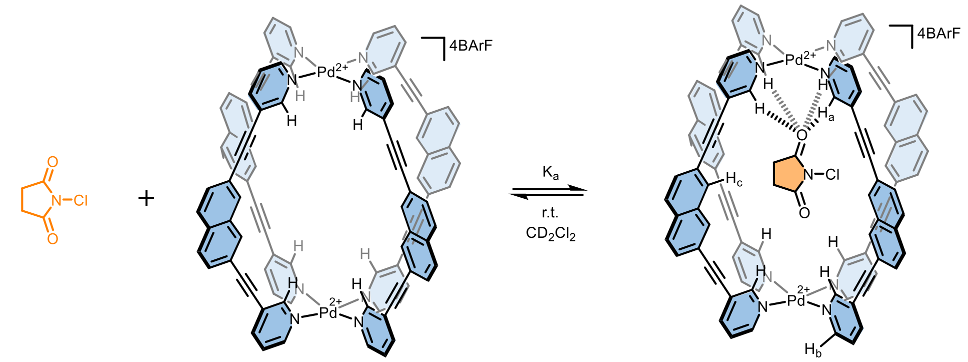


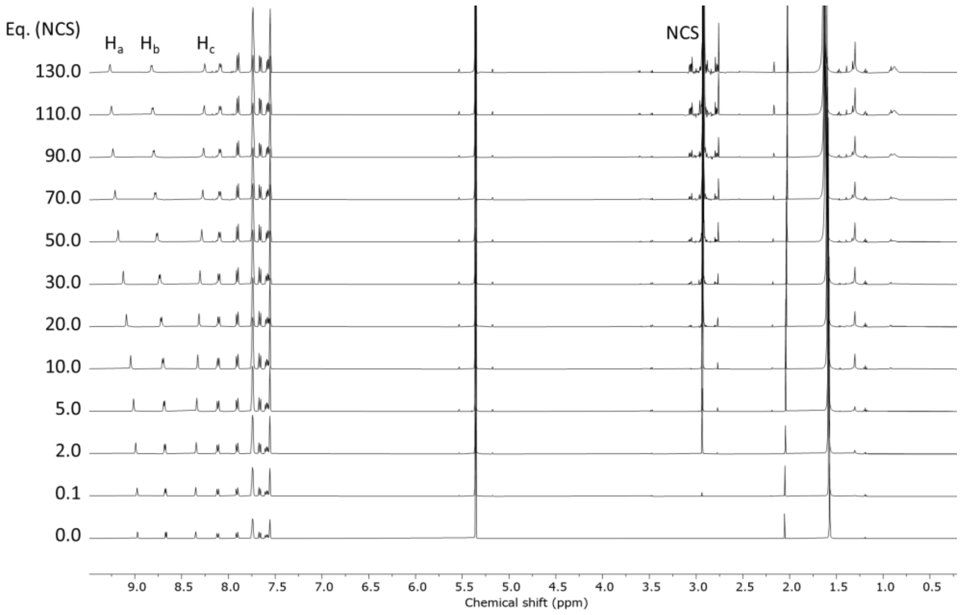


*Figure S30. $^1$H NMR (400 MHz, $CD_2Cl_2$) spectroscopic data for titration of NCS into **C3**.*

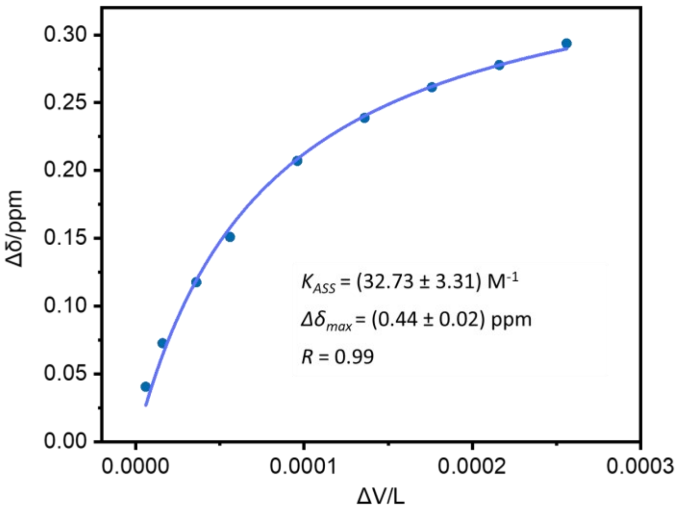


*Figure S31. Fitted data for change in internal peak position ($H_a$) with increasing NCS concentration.*

## 3.5 Titration of Triphenylphosphine oxide into **C3**

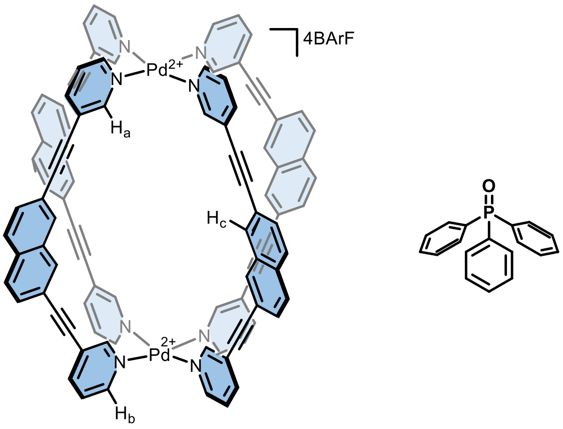


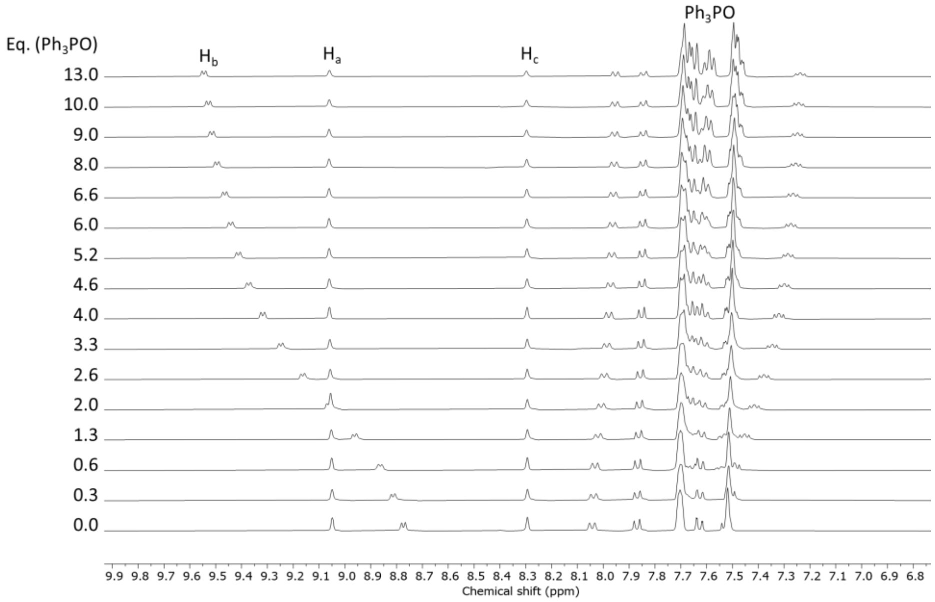


*Figure S32. $^{1}$H NMR (400 MHz, $CD_2Cl_2$) spectroscopic data for titration of triphenylphosphine oxide into **C3**.*

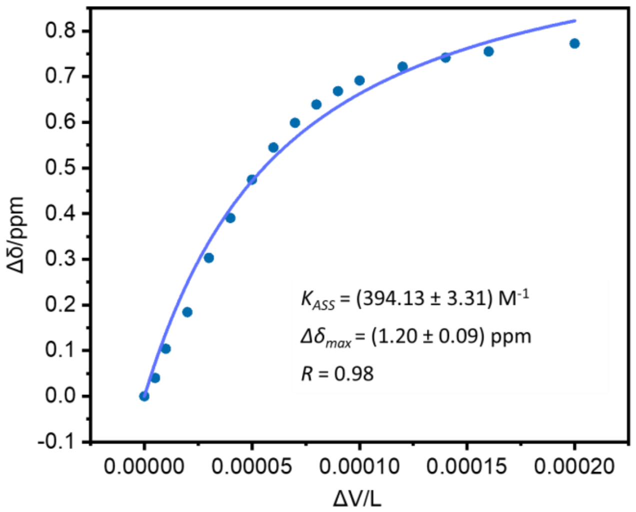


*Figure S33. Fitted data for change in external peak position ($H_b$) with increasing triphenylphosphine oxide concentration.*

# 4. Michaelis-Menten analysis

## 4.1 General procedure

### 4.1.1 Michaelis-Menten kinetics

The experimental data from variable concentrations of NCS was fitted to the Michaelis-Menten kinetics, which is an essential model for enzyme reactions.

$$E + S \underset{k_r}{\overset{k_f}{\rightleftharpoons}} ES \xrightarrow{k_{cat}} E + P$$

$$v = \frac{d[P]}{dt} = V_{max}\frac{[S]}{K_M + [S]} = k_{cat}[E]_0\frac{[S]}{K_M + [S]}$$

*Equation S5*

As only NCS can bind with cage, the subsequent analysis was conducted using the non-linearised form of the Michaelis-Menten equation (equation S6) by plotting initial rates vs initial concentrations of NCS.

$$v = \frac{d[P]}{dt} = V_{max}\frac{[NCS]}{K_M + [NCS]}$$

*Equation S6*

Under saturation conditions, $k_{cat}$ can then be derived from $V_{max}$, $[cage]_0$ and $[\mathbf{1b}]_0$ according to:

$$\text{Cage} + \text{NCS} \underset{k_r}{\overset{k_f}{\rightleftharpoons}} \text{Cage@NCS} \xrightarrow[\mathbf{1b}]{k_{cat}} \text{Cage} + \text{P}$$

$$V_{max} = k_{cat}[\mathbf{1b}][cage]$$

*Equation S7*

### 4.1.2 Cage catalyzed reaction

Considering the very different association constants for NCS with **C1** and **C3**, differentg saturation conditions were used. For **C1** reactions, the concentration of **1b** and cage were fixed as 10 mM and 1 mM, respectively, and variable concentrations of NCS from 0.5 – 5 mM were used. For **C3**, the concentration of **1b** and cage were also fixed as 10 mM and 1 mM, and variable concentrations of NCS from 5 – 400 mM were introduced in a series of reactions. The data was analyzed using linear fits over the early stage to obtain initial rates.

### 4.1.3 Uncatalyzed reaction

Due to the much slower uncatalyzed reaction, higher concentrations of the two reactants were used: 40 mM of **1b** and 400 mM of NCS. The kinetic constant ($k_{uncat}$) of the uncatalyzed reaction was determined by fitting the concentration of **2b** experimentally determined by $^{1}$H NMR spectroscopy at different reaction time to the integrated second order reaction law (Equation S8). This was carried out using the Levenberg-Marquardt Nonlinear Least-Squares Algorithm implemented in Origin software interface.[S18]

$$[\mathbf{2b}] = \frac{[\mathbf{1b}]_0 \cdot [NCS]_0 \cdot (1 - \exp(([NCS]_0 - [\mathbf{1b}]_0) \cdot k_{uncat} \cdot t))}{[\mathbf{1b}]_0 - [NCS]_0 \cdot \exp(([NCS]_0 - [\mathbf{1b}]_0) \cdot k_{uncat} \cdot t)}$$

*Equation S8*

## 4.2 Kinetic profiles and $^1$H NMR spectra of **1b** using variable [NCS]

### 4.2.1 Kinetic profiles and $^1$H NMR spectra for the uncatalyzed reaction

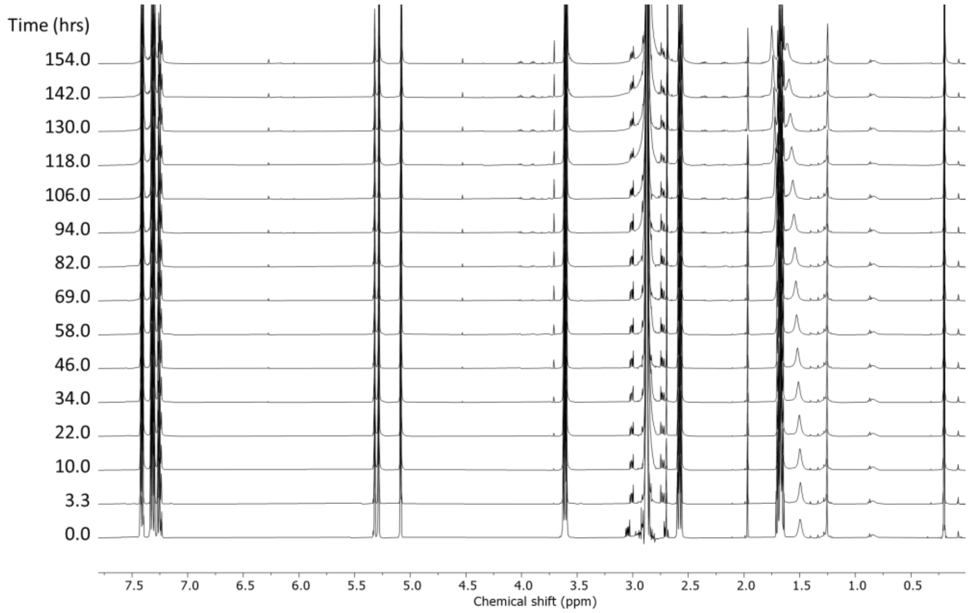


*Figure S34. $^1$H NMR spectra (600 MHz, $CD_2Cl_2$) for the reaction of **1b** (40 mM) and NCS (400 mM).*

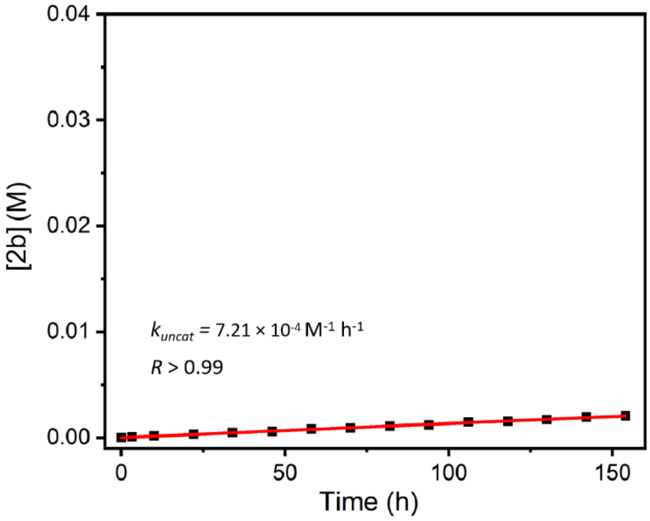


*Figure S35. Kinetic profile for the uncatalyzed reaction of **1b** (40 mM) and NCS (400 mM). The red line is the fit to the integrated second order reaction law (Equation S8).*

### 4.2.2 Kinetic profiles and $^1$H NMR spectra with **C1** using variable [NCS]

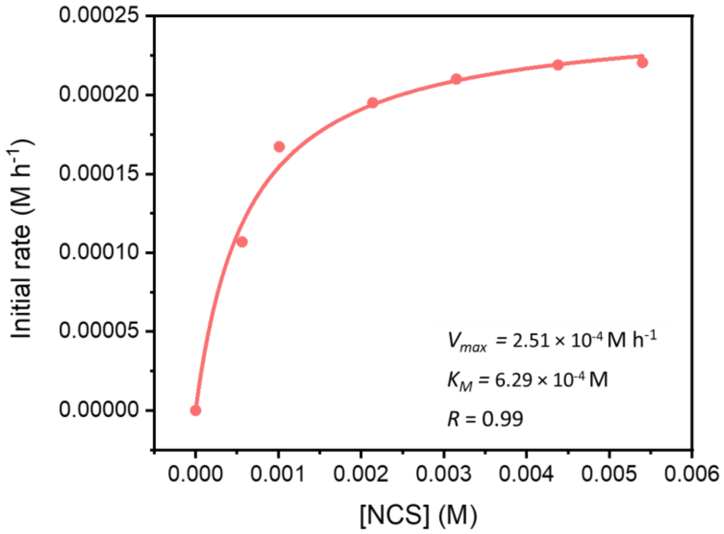


*Figure S36. Saturation kinetics for variable concentrations of NCS with **C1**. The solid line is the fit to the Michaelis-Menten equation (Equation S6).*

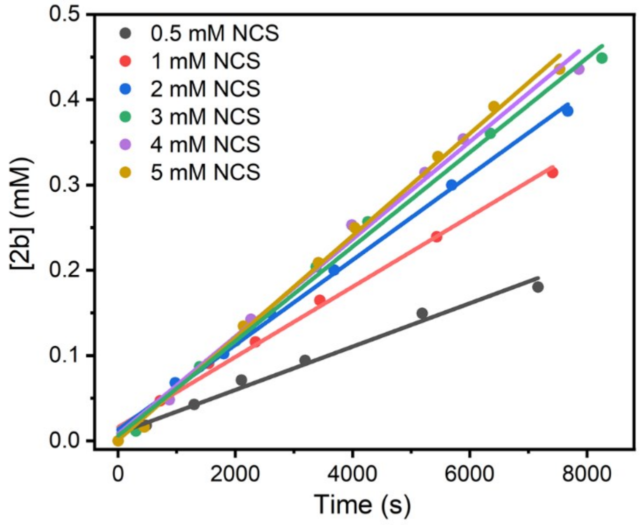


*Figure S37. Linear fitting for the initial rate of 10 mM **1b** and 0.5-5 mM NCS with 1 mM **C1**.*

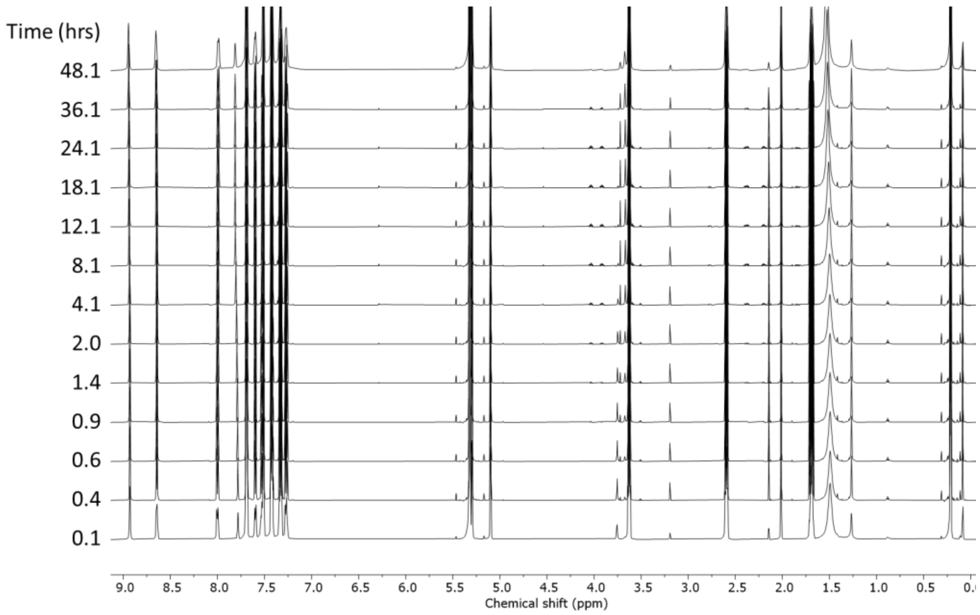


*Figure S38.* [1]*H NMR spectra (600 MHz, $CD_2Cl_2$) for the reaction of* ***1b*** *(10 mM) and NCS (0.5 mM) with* ***C1*** *(1 mM).*

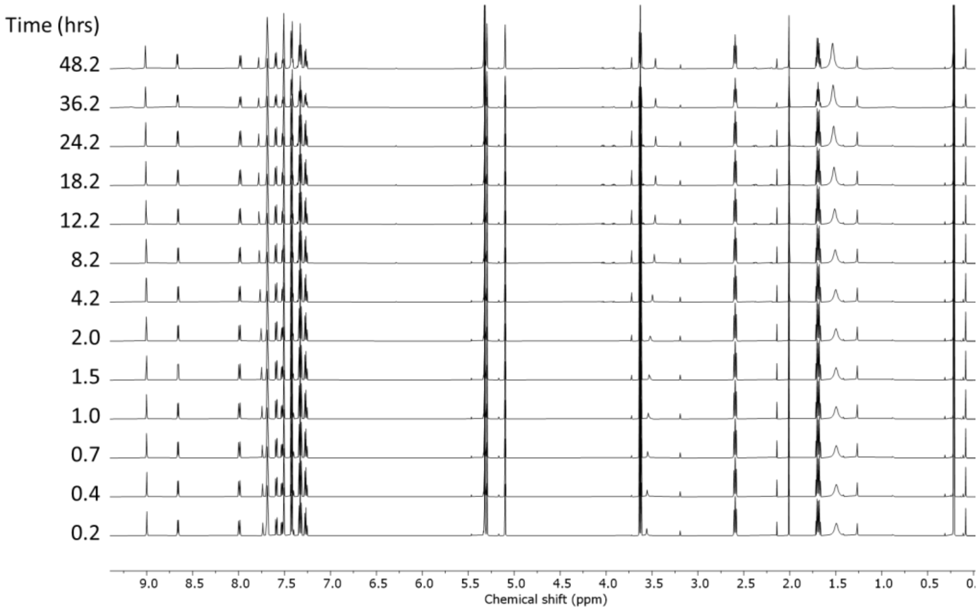


*Figure S39.* [1]*H NMR spectra (600 MHz, $CD_2Cl_2$) for the reaction of* ***1b*** *(10 mM) and NCS (1 mM) with* ***C1*** *(1 mM).*

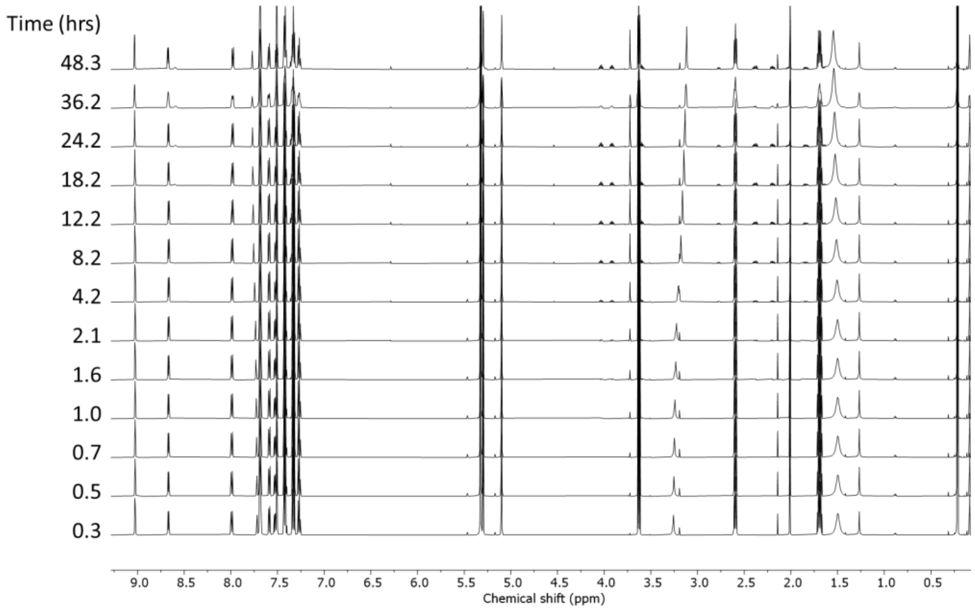


*Figure S40. $^{1}$H NMR spectra (600 MHz, $CD_2Cl_2$) for the reaction of **1b** (10 mM) and NCS (2 mM) with **C1** (1 mM).*

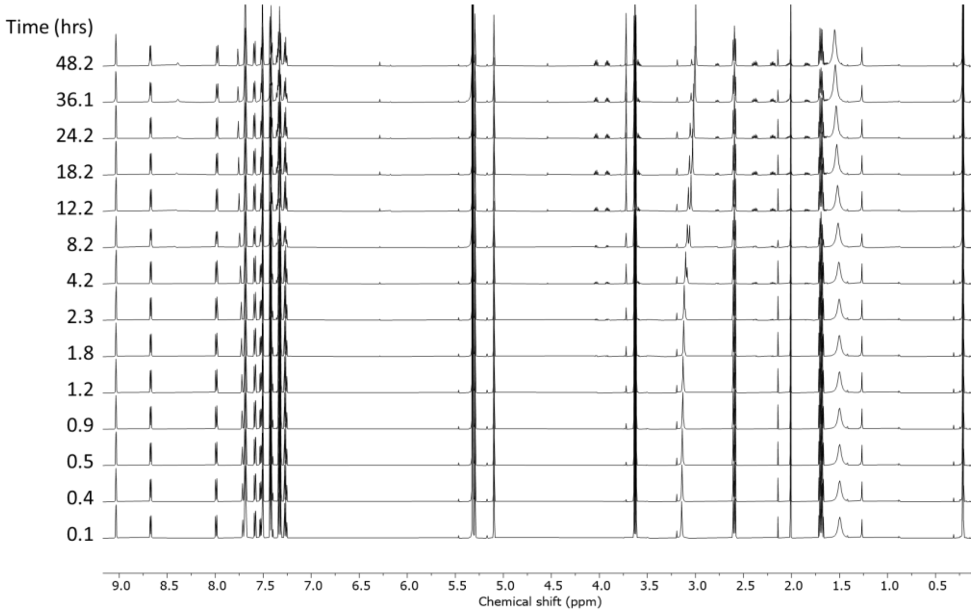


*Figure S41. $^{1}$H NMR spectra (600 MHz, $CD_2Cl_2$) for the reaction of **1b** (10 mM) and NCS (3 mM) with **C1** (1 mM).*

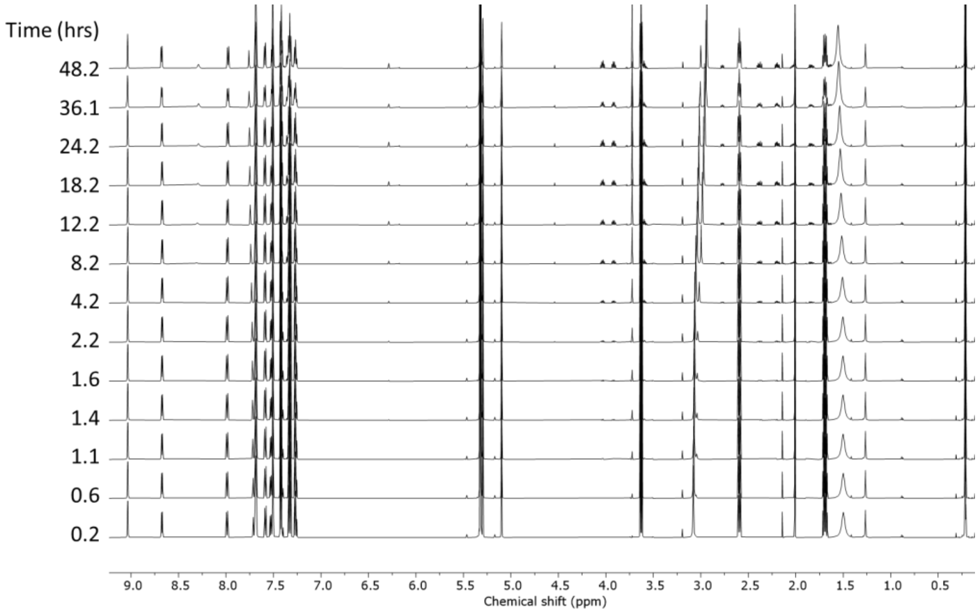


*Figure S42. $^{1}$H NMR spectra (600 MHz, $CD_2Cl_2$) for the reaction of **1b** (10 mM) and NCS (4 mM) with **C1** (1 mM).*

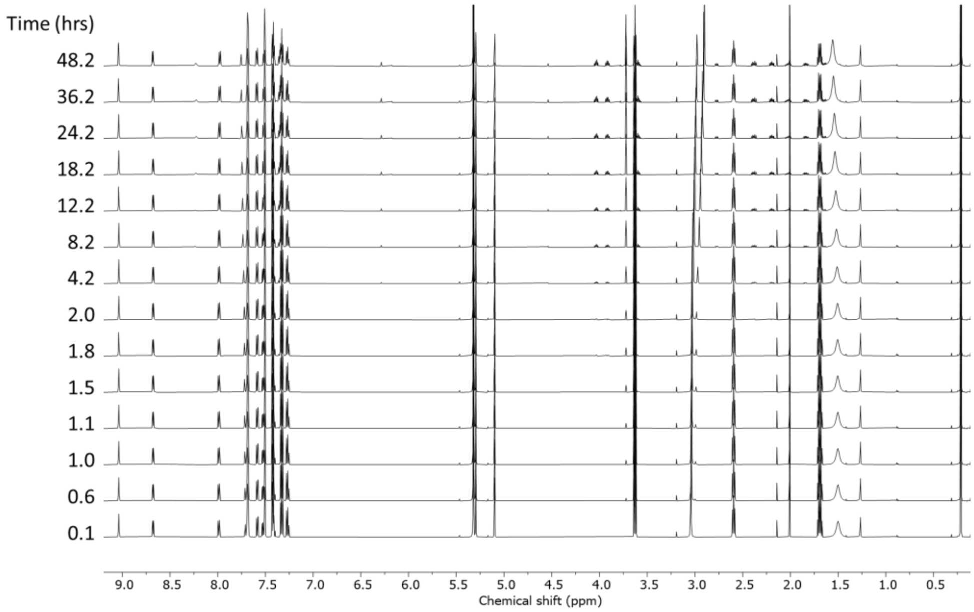


*Figure S43. $^{1}$H NMR spectra (600 MHz, $CD_2Cl_2$) for the reaction of **1b** (10 mM) and NCS (5 mM) with **C1** (1 mM).*

### 4.2.3 Kinetic profiles and $^1$H NMR spectra with **C3** using variable [NCS]

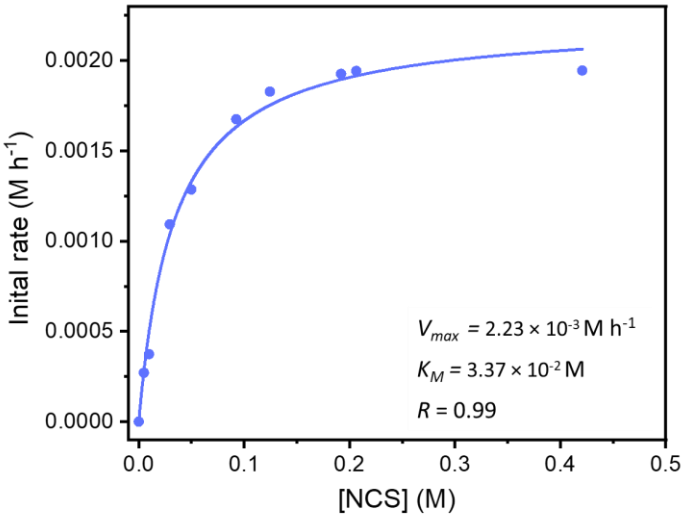


*Figure S44. Saturation kinetics for variable concentrations of NCS with **C3**. The solid line is the fit to the Michaelis-Menten equation (Equation S6).*

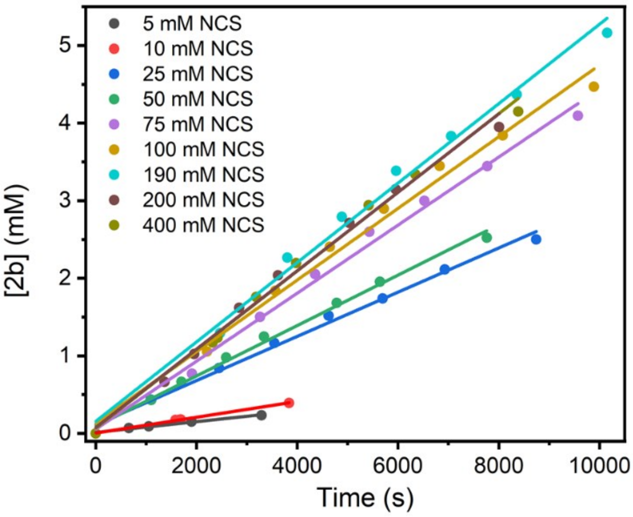


*Figure S45. Linear fittings for the initial rate of 10 mM **1b** and 5-400 mM NCS with 1 mM **C3**.*

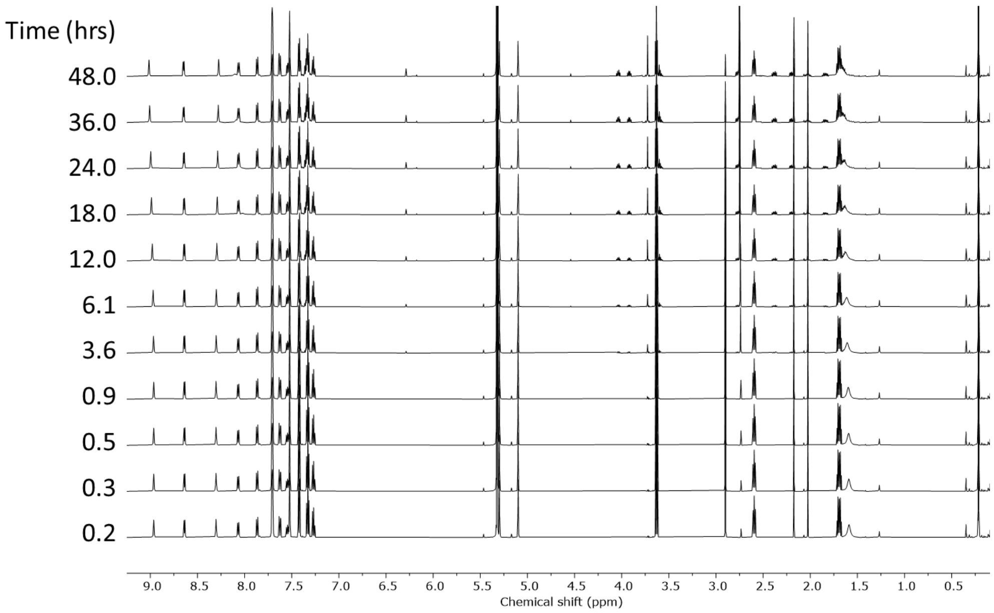


*Figure S46. $^{1}$H NMR spectra (600 MHz, $CD_2Cl_2$) for the reaction of **1b** (10 mM) and NCS (5 mM) with **C3** (1 mM).*

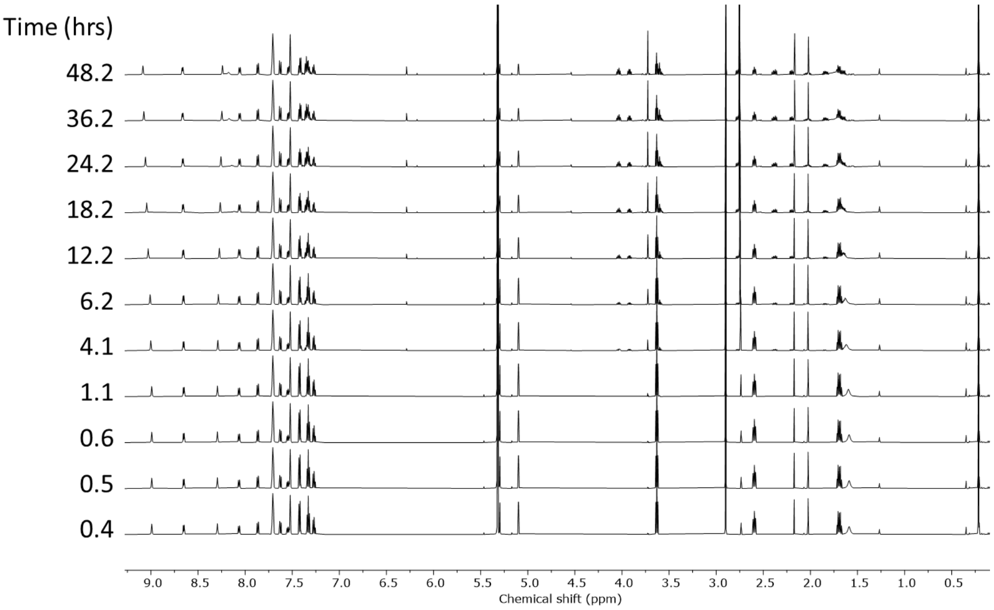


*Figure S47. $^{1}$H NMR spectra (600 MHz, $CD_2Cl_2$) for the reaction of **1b** (10 mM) and NCS (10 mM) with **C3** (1 mM).*

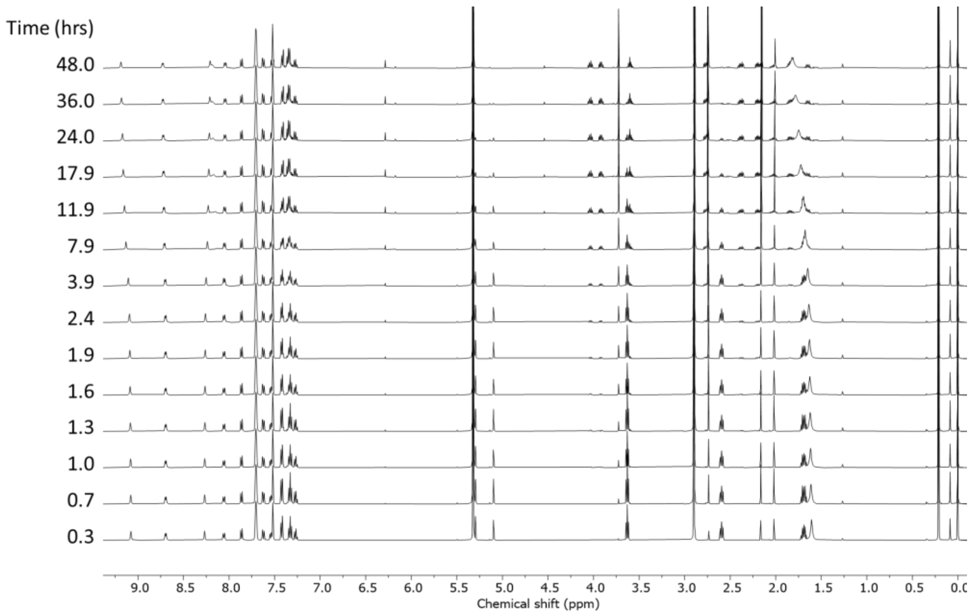


*Figure S48. $^{1}$H NMR spectra (600 MHz, $CD_2Cl_2$) for the reaction of **1b** (10 mM) and NCS (25 mM) with **C3** (1 mM).*

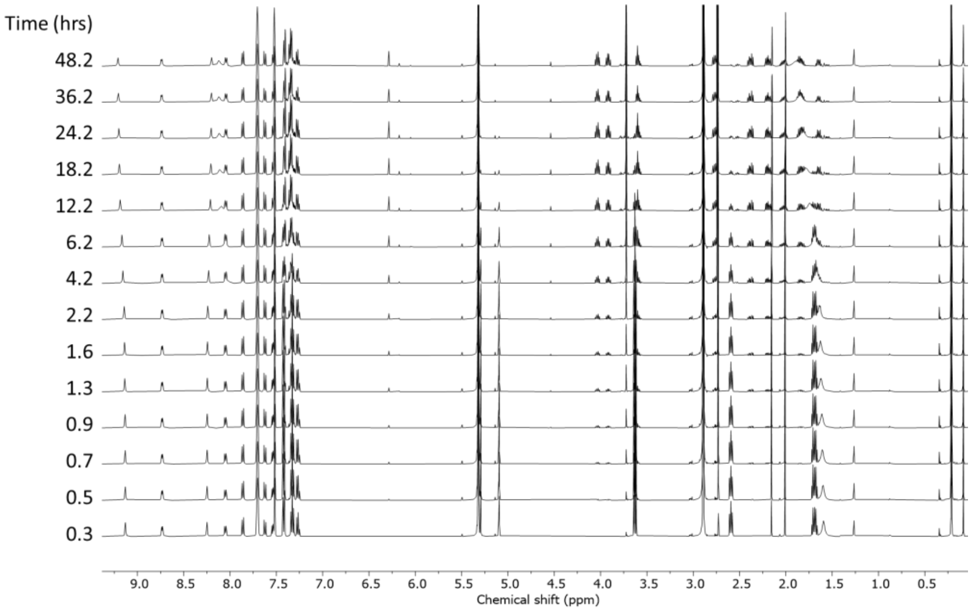


*Figure S49. $^{1}$H NMR spectra (600 MHz, $CD_2Cl_2$) for the reaction of **1b** (10 mM) and NCS (50 mM) with **C3** (1 mM).*

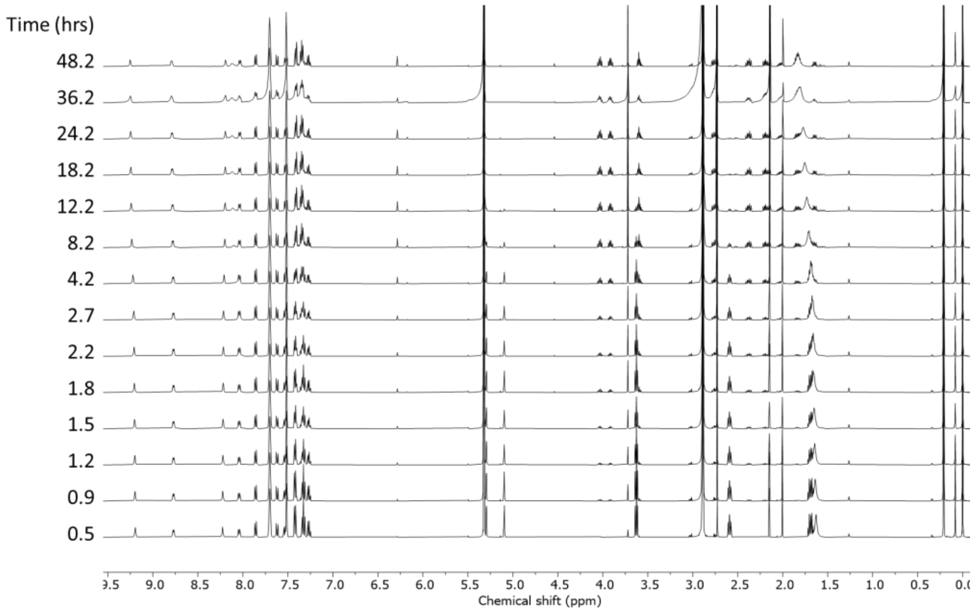


*Figure S50. $^{1}$H NMR spectra (600 MHz, $CD_2Cl_2$) for the reaction of* ***1b*** *(10 mM) and NCS (75 mM) with* ***C3*** *(1 mM).*

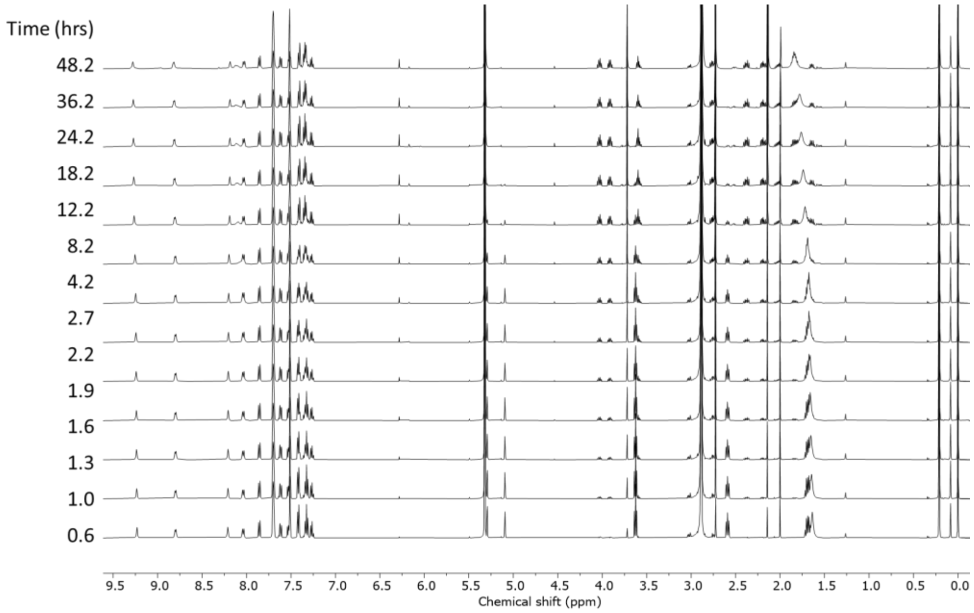


*Figure S51. $^{1}$H NMR spectra (600 MHz, $CD_2Cl_2$) for the reaction of* ***1b*** *(10 mM) and NCS (100 mM) with* ***C3*** *(1 mM).*

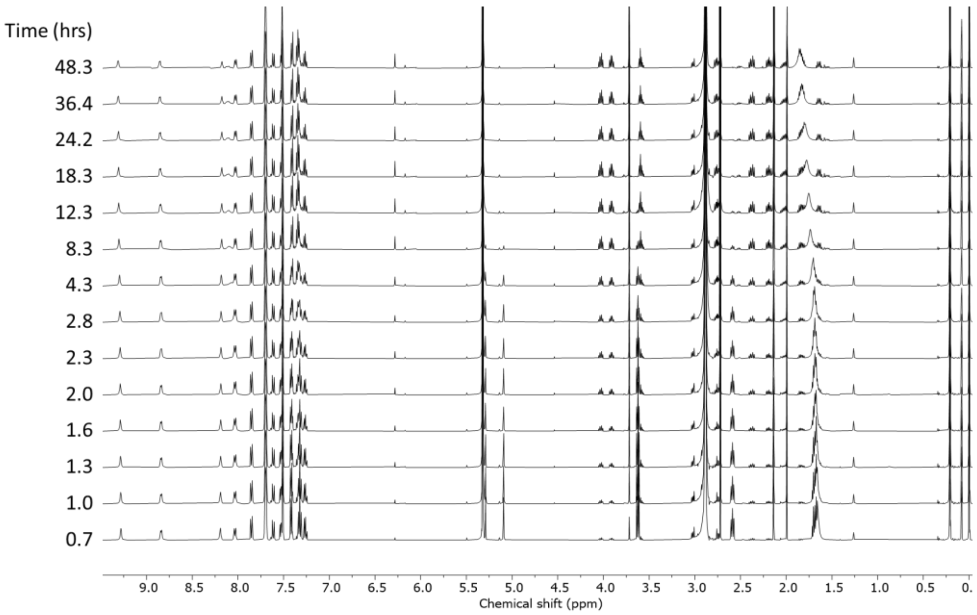


*Figure S52. $^{1}$H NMR spectra (600 MHz, $CD_2Cl_2$) for the reaction of **1b** (10 mM) and NCS (190 mM) with **C3** (1 mM).*

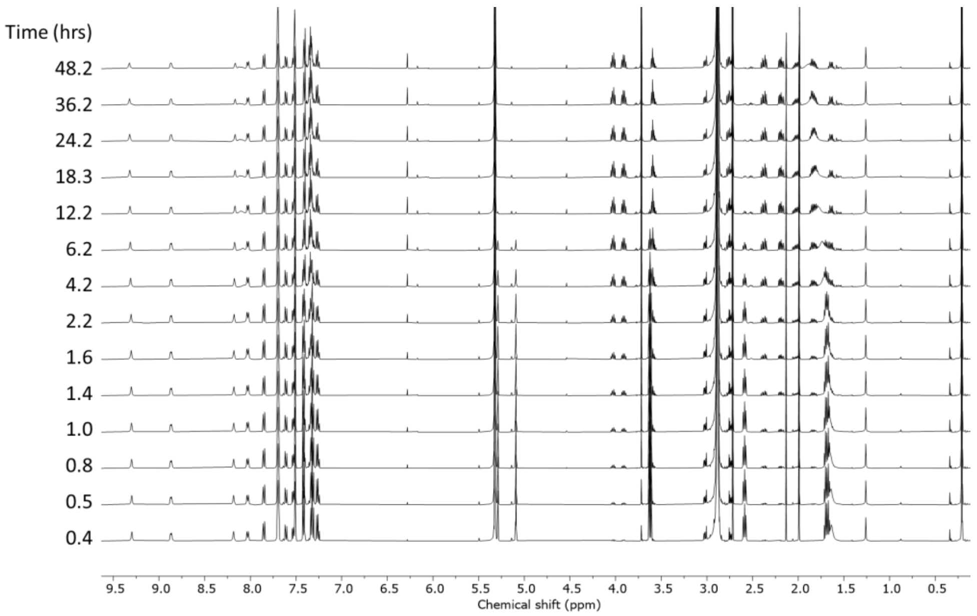


*Figure S53. $^{1}$H NMR spectra (600 MHz, $CD_2Cl_2$) for the reaction of **1b** (10 mM) and NCS (200 mM) with **C3** (1 mM).*

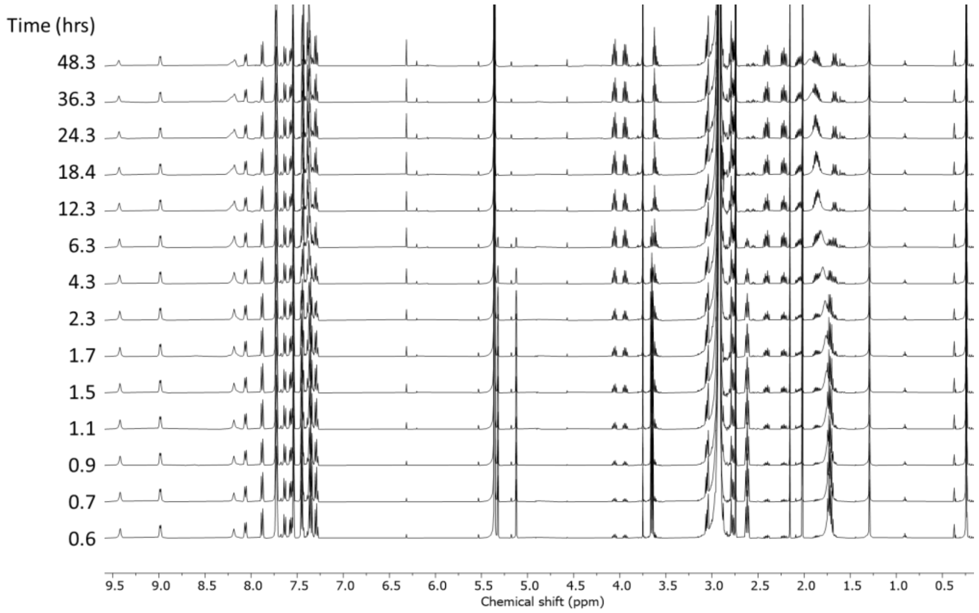


*Figure S54. $^1$H NMR spectra (600 MHz, $CD_2Cl_2$) for the reaction of **1b** (10 mM) and NCS (400 mM) with **C3** (1 mM).*

## 4.3 Table of Michaelis-Menten constants

*Table S3. Michaelis-Menten constants of the reaction of **1b** to **2b**.*

| Entry | Cage | $V_{max}$ (M h$^{-1}$) | $K_M$ (M) | $k_{cat}$ (M$^{-1}$ h$^{-1}$) | $k_{cat}$ (M$^{-1}$ s$^{-1}$) | $k_{cat}/K_M$ (M$^{-2}$ h$^{-1}$) | $k_{cat}/k_{uncat}$ |
|---|---|---|---|---|---|---|---|
| 1 | **C1** | $2.51 \times 10^{-4}$ | $6.29 \times 10^{-4}$ | 26.72 | $7.42 \times 10^{-3}$ | $4.25 \times 10^{4}$ | $3.71 \times 10^{4}$ |
| 2 | **C3** | $2.23 \times 10^{-3}$ | $3.37 \times 10^{-2}$ | 217.23 | $6.03 \times 10^{-2}$ | $6.44 \times 10^{3}$ | $3.17 \times 10^{5}$ |

# 5. Chlorination between **5** and NCS

## 5.1 General procedure

Due to the slow uncatalyzed reaction, higher concentrations of two reactants were used: 50 mM of **5** and 200 mM of NCS. The kinetic constant ($k_{uncat}$) of the uncatalyzed reaction was obtained by fitting the increasing concentration of succinimide experimentally determined by $^1$H NMR spectroscopy at different reaction time to the integrated second order reaction law.

$$[succinimide] = \frac{[NCS]_0 \cdot [\mathbf{5}]_0 \cdot (1 - \exp(([\mathbf{5}]_0 - [NCS]_0) \cdot k_{uncat} \cdot t))}{[NCS]_0 - [\mathbf{5}]_0 \cdot \exp(([\mathbf{5}]_0 - [NCS]_0) \cdot k_{uncat} \cdot t)}$$

*Equation S9*

For the catalyzed reaction, cage-saturation conditions were used: 10 mM substrate **5**, 200 mM NCS and 1 mM cages. Under these conditions, where [NCS@cage] remains approximately constant over the course of the reaction, the consumption of **5** was fit to a modified pseudo 1st order rate equation:

$$[\mathbf{5}] = [\mathbf{5}]_0 e^{-k_{obs} t}$$

*Equation S10*

where $k_{obs}$ is the observed rate constant ($h^{-1}$). The 2$^{nd}$ order rate constant of the rate determining step $k_{cat}$ could then be calculated based on the following assumptions:

$$\text{rate} = k_{cat}[\mathbf{5}][NCS \subset cage]$$
$$\text{rate} = k_{obs}[\mathbf{5}]$$
$$k_{obs} = k_{cat}[NCS \subset cage]$$
$$[NCS \subset cage] = [cage]$$

## 5.2 NMR scale reaction yields

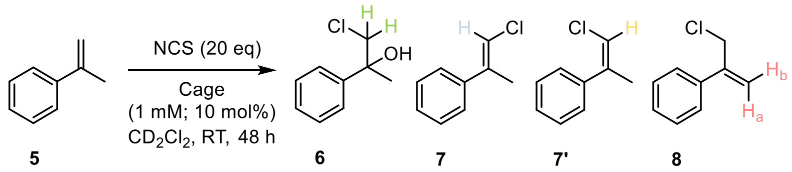


| | **6** (%)[a] | **7** (%) | **7'** (%) | **8** (%) |
|---|---|---|---|---|
| **C1** | 12 | 27 | 8 | 26 |
| **C3** | 16 | 40 | 5 | 33 |

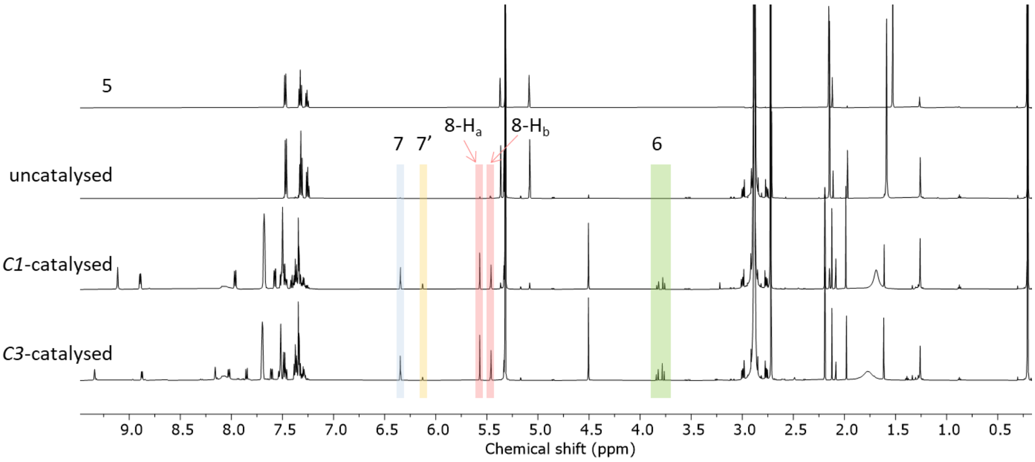

*Figure S55. Cage catalyzed chlorination of **5**, NMR scale reaction yields under the following conditions: 1 mM cage, 10 mM **5**, 200 mM NCS, $CD_2Cl_2$, r.t.. 48 h. Yield determined by $^1$H NMR spectroscopy integration against internal standard, tetrakis(trimethylsilyl)silane. Products were identified by a comparison to literature data.*[S19,S20,S14]

## 5.3 Kinetic profiles and $^1$H NMR spectra of **5** and NCS

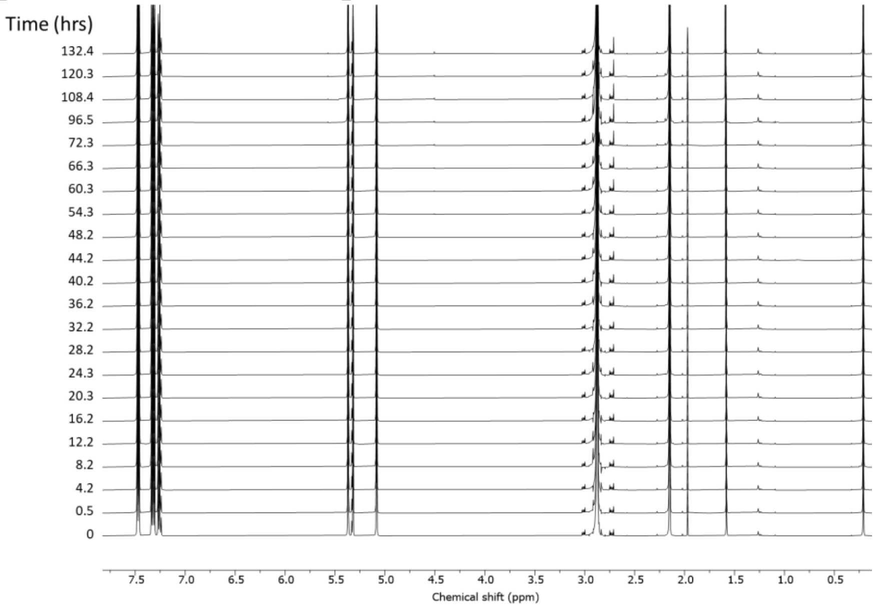


*Figure S56. $^1$H NMR spectra (600 MHz, $CD_2Cl_2$) for the reaction of **5** (50 mM) and NCS (200 mM).*

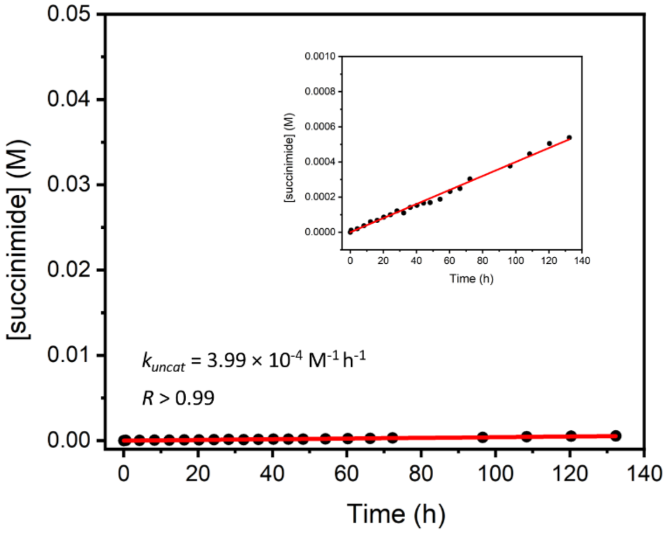


*Figure S57. Kinetic profile for **5** and NCS. The solid line is the fit to the integrated second-order rate equation.*

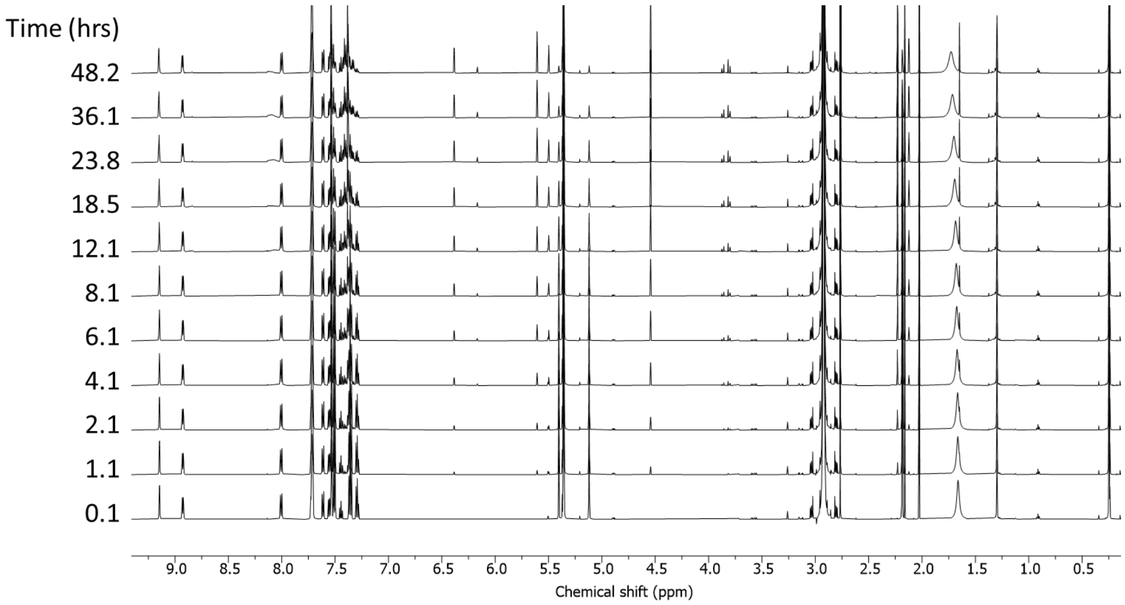


*Figure S58. $^{1}$H NMR spectra (600 MHz, $CD_2Cl_2$) for the reaction of **5** (10 mM) and NCS (200 mM) with **C1** (1 mM).*

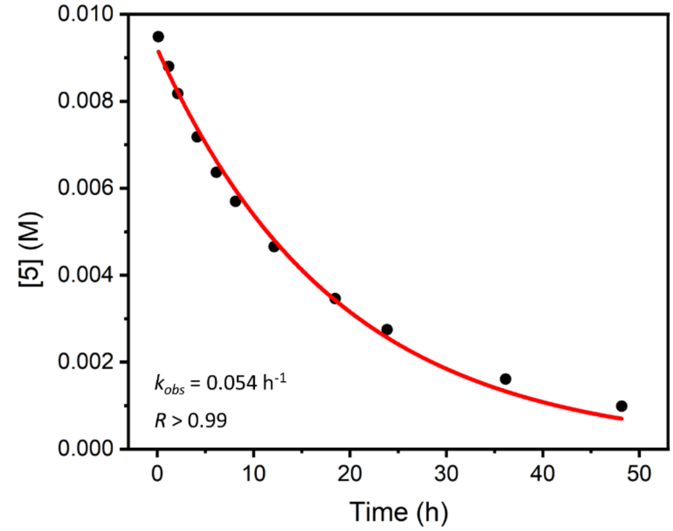


*Figure S59. Kinetic profile for **5** and NCS with **C1**. The solid line is the fit to the pseudo first order equation.*

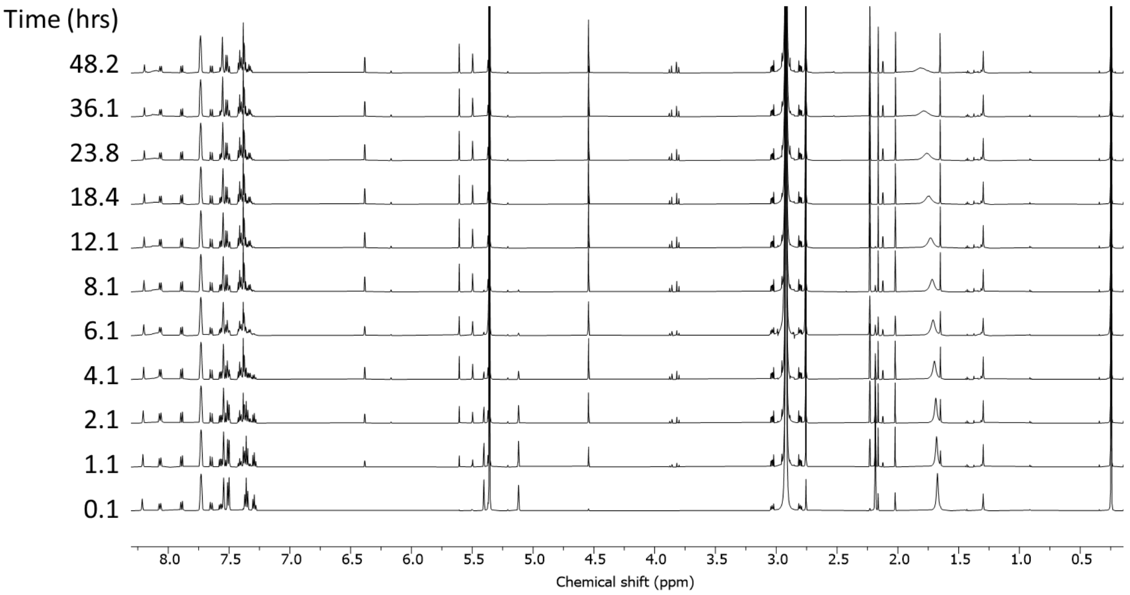


*Figure S60.* [1]*H NMR spectra (600 MHz,* $CD_2Cl_2$*) for the reaction of* ***5*** *(10 mM) and NCS (200 mM) with* ***C3*** *(1 mM).*

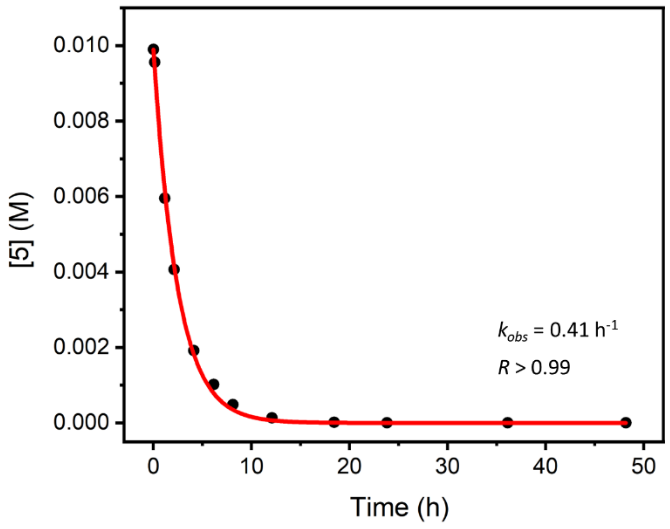


*Figure S61. Kinetic profile for* ***5*** *and NCS with* ***C3****. The solid line is the fit to the pseudo first order rate equation.*

## 5.4 Table of the kinetic constants for chlorination of 5.

*Table S4. The kinetic constants for the cage-catalyzed chlorination of* ***5*** *with NCS*

| Entry | Cage | $k_{obs}$ ($h^{-1}$) | $k_{cat}$ ($M^{-1}$ $h^{-1}$) | $k_{cat}$ ($M^{-1}$ $s^{-1}$) | $k_{uncat}$ ($M^{-1}$ $s^{-1}$) | $k_{cat}$ /$k_{uncat}$ |
|---|---|---|---|---|---|---|
| 1 | - | - | - | - | $1.11 \times 10^{-7}$ | - |
| 2 | **C1** | 0.054 | 68.3 | $1.90 \times 10^{-2}$ | - | $1.71 \times 10^{5}$ |
| 3 | **C3** | 0.41 | 762 | $2.12 \times 10^{-1}$ | - | $1.91 \times 10^{6}$ |

# 6. Computational methods

## 6.1 Method

**Force-field parameters**. Cages were parameterized using *metallicious,*[S21] assigning GAFF force-field[S22] parameters for organic linker and Li-Merz parameters of palladium in OPC water.[S23] $BArF^{-}$ parameters were taken from our previous work.[S24] Substrates were parameterized with GAFF force-field using AM1-BCC charges.

**Conventional Molecualr Dynamics (MD) simulations**. MD simulations were performed with GROMACS (v 2021.3).[S25,S26] The systems were initiated in a cubic box with edge length of 5.0 nm. Parameters for dichloromethane (DCM) were obtained from VirtualChemistry.org.[S27] Additionally, 17 OPC[S28] water molecules were added as impurities to DCM. The systems were neutralized with $BArF^{-}$ as a counter ion and solvated in DCM. The system was energy-minimized for 5000 steps or until the maximum force was below 10 kJ $mol^{-1}$ $nm^{-1}$. Three replicas were initiated from the minimized system by assigning random velocities at 300 K. Each replica was subjected to 200 ps (2 fs stepsize) NVT equilibration and 200 ps (2 fs stepsize) NPT equilibration at 300 K and 1.0 bar before a production run of 100 ns (2 fs stepsize), during which coordinates were saved every 10 ps. Production runs were performed using the v-rescale thermostat[S29] and c-rescale barostat.[S30] Long-range electrostatics were treated using smooth particle mesh Ewald.[S31,S32] A 1.0 nm cut-off was applied for van der Waals interactions and short-range electrostatics. Analyses were performed using MDAnalysis.[S33,S34]

**Umbrella sampling (US) simulations**: MD/US was employed to investigate the binding of alcohol inside the **C1** and **C3** cages. Three replicas of MD/US simulations were performed, each initiated with the alcohol substrate **1b** inside the cage. The initial structures for this calculation were generated from a steered MD simulation in which the center of mass (CoM) of the alcohol was pulled away from the cage's CoM along the *z*-axis over ~0.5 ns, using a pulling rate of 0.005 nm $ps^{-1}$ and applying constraints in all direction with a force constant of 1000 kJ $mol^{-1}$ $nm^{-2}$. From these trajectories, frames were extracted at 1 Å interval of alcohol-cage distance to generate the starting configurations for the US windows. For each window, 60 ns MD simulations were performed with alcohol distance constrained in three dimensions, using a force constant of 1000 kJ $mol^{-1}$ $nm^{-2}$. If the histogram overlap was insufficient, additional windows were added and simulations were conducted with a higher force constant of 5000 kJ $mol^{-1}$ $nm^{-2}$ at the distance of interest. In total, these simulations amounted to ~7 µs per system. After discarding the first 10 ns for equilibration, the resultant potentials of mean force (PMF) were constructed with the weighted histogram analysis method (WHAM)[S35] and corrected for entropic effect due to the constraint of three degrees of freedom.[S36]

**Quantum mechanical (QM) calculations.** All QM calculations were performed using ORCA (v 5.0.3).[S37,S38] Geometry optimizations were carried out with the composite PBEh-3c functional;[S39] this method was chosen due to its computational efficiency and good performance or reaction modeling.[S39] Vibrational frequencies were calculated at the same level of theory to confirm whether the structures correspond to a minimum or a transition state (TS) and to evaluate the zero-point vibrational energy (ZPVE) and thermal corrections at 298 K using the quasi-rigid-rotor-harmonic oscillator with threshold parameter $\omega_0$ set to the default value of 100 $cm^{-1}$.[S40] Single-point energies were obtained at the M06-2X functional[S41] with a def2-TZVP basis set. This level of theory was chosen based on its demonstrated good performance when applied to supramolecular complexes,[S42] as well as on our benchmark study for the uncatalyzed reactions using the CPCM(DCM)-DLPNO-CCSD(T)/def2-TZVPP as a reference (RMSE = 1.3 kcal $mol^{-1}$; Table S6 and Table S10). Solvent effects were

accounted for using the CPCM implicit solvent model[S43] with parameters specific to DCM, as implemented in ORCA.

For the cage-free reaction, TSs were identified using two methods: (a) the climbing image nudged elastic band (NEB) method, followed by TS optimization for chloretherification and (b) a 1D energy scan along the reactive Cl−N bond followed by TS optimization for α-methylstyrene chlorination. A guess TS was generated for the reaction within the cage by placing the TS obtained from the cage-free reaction into the cage. The structures were optimized with reactive bond constrained using the “TS analogue” approach,[S44] followed by subsequent unconstrained optimization to the TS at the CPCM(DCM)-PBEh-3c level of theory. All single-point energy calculations and thermal contributions for the systems studied in this work are presented in Table S14.

## 6.2 Chlorocycloetherification

### 6.2.1 Cage Binding

**C1 cage**. To evaluate the ability of the substrates, N-chlorosuccinamide (NCS) and alcohol **1b,** to bind inside **C1**, three independent 100 ns MD simulations were conducted in explicit DCM, which included 17 OPC water molecules as impurities. The results revealed two binding modes for NCS within the cage: NCS positioned between the linker’s benzene rings, which appears to stabilize NCS through van der Waals interactions (**A**, Figure S62b) and NCS positioned in the center of the cage, with its two carbonyl groups interacting with the cage’s C-H bonding pockets (**B**, Figure S62b). Subsequent DFT optimization of both structures converged to a single conformer resembling conformation **B** from MD simulations (Figure S62c).

During the MD simulations, the alcohol did not bind to the cavity. The PMF for the pulling of the alcohol from inside the cage obtained from MD/US simulations resulted in a positive binding free energy ($\Delta G_{\text{binding}}$ = ~4 kcal mol$^{-1}$; Figure S62d), confirming that the binding of alcohol inside the cage is unfavorable.

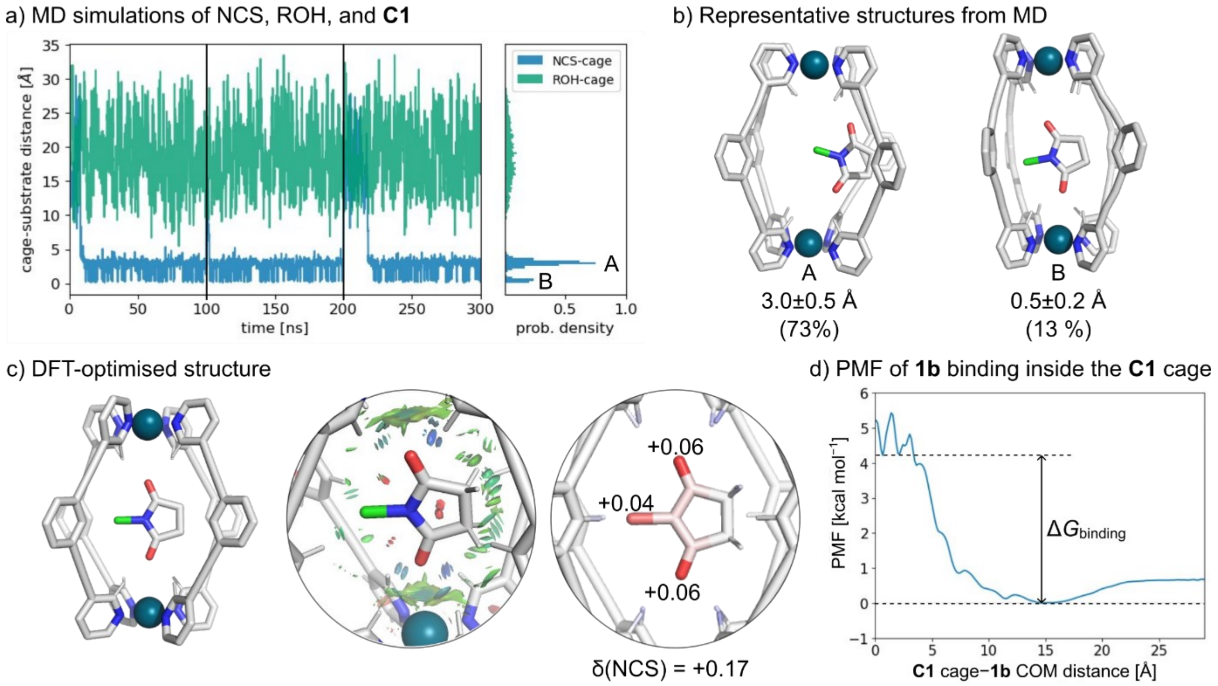


*Figure S62 Substrate binding in **C1** cage. (a) Evolution of the center of mass (CoM) distance between the cage and NCS (blue) and alcohol (green) during 300ns MD simulation; probability density is depicted on the right-hand side. (b) Bound structures of NCS obtained for the COM **C1**−NCS distance of 3.0 Å (A) and 0.5 Å (B). (c) Structure of NCS bound to **C1** (left) optimized at CPCM(DCM)-PBEh-3c level of theory, zoom in depicting non-covalent interaction (NCI) plot (center) and distribution of partial charges (right). (d) The potential of the mean force (PMF) for the binding of alcohol inside the cage obtained from US/MD simulations using 136 windows 50 ns each.*

**C3 cage.** Using a similar protocol to the one described for **C1**, 3×100 ns MD simulations with the **C3** cage with NCS revealed that for 86% time of simulation, NCS remained bound to the **C3** cage (distance < 5 Å; Figure S63a). In this case, NCS is predominantly located between the naphthalene groups (conformer **C**, Figure S63b), similar to the conformation **A** observed in **C1**. In 16 frames (0.5% of total frames), NCS occupied one of the C-H pockets, while a water molecule occupied the second pocket (conformer **D**, Figure S63b). In all cases, the alcohol 1b predominantly remains in solution, which has been confirmed by PMF obtained from MD/US simulations showing unfavorable binding ($\Delta G_{\text{binding}}$ = 1 kcal mol$^{-1}$; Figure S63d). In only one frame, NCS was positioned between the two naphthalene rings, and the alcohol interacting with hydrogens in the Pd-pocket (conformer **E**, Figure S63b).

The **C**, **D,** and **E** conformations were extracted and optimized at the CPCM(DCM)-PBEh-3c level of theory. Additionally, a hypothetical configuration with NCS bound within the C-H pocket, with the water molecule removed, was also optimized. The results show that NCS binds more strongly between the naphthalene rings than in the C-H pocket, with a binding free energy difference (ΔΔG) of 3.5 kcal mol$^{-1}$ at CPCM(DCM)-M06-2X/def2-TZVP//CPCM(DCM)-PBEh-3c level of theory (Table S5). However, this finding contrasts with $^{1}$H NMR experiments, which revealed a significant shift in the signals associated with the C-H pocket, suggesting that NCS likely binds there. Consequently, we selected the conformation with NCS bound within the C-H-pocket as the reference structure.

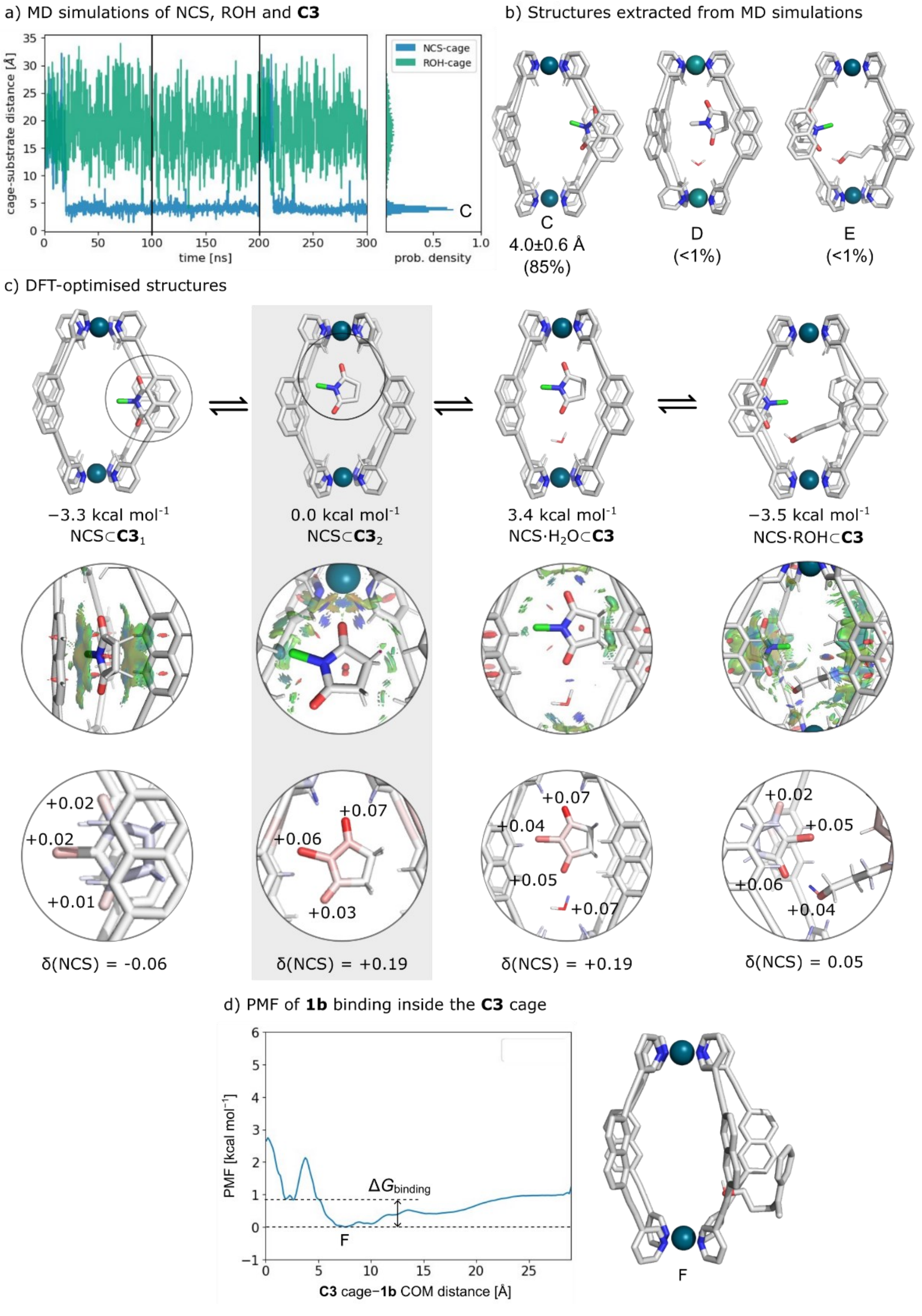


*Figure S63 Substrate binding in **C3** cage. (a) Evolution of the center-of-mass (COM) distance between the **C3** cage and NCS (blue)/alcohol (green) during 3×100ns MD simulation with the probability density depicted on the right-hand side. (b) Representative binding modes of NCS bound to **C3,** corresponding to **C3**-NCS having a COM distance of 4.0 Å (**C**); NCS·$H_2O$ complex within **C3** (**D**), and NCS·**1b** complex within **C3** (**E**). (c) Structures and relative energies of different binding modes of NCS within the cage obtained at CPCM(DCM)-M06-2X/def2-TZVP//CPCM(DCM)-PBEh-3c level of theory (top). The zoomed-in view depicts non-covalent interaction (NCI) plots (center) and partial charges (bottom). (d) The potential of the mean force (PMF) for the binding of alcohol inside the cage obtained from US/MD simulations using 116 windows of 50 ns.*

*Table S5 Relative binding energies (kcal mol-1) for different binding modes identified during MD simulations computed at the CPCM(DCM)-M06-2X/def2-TZVP//CPCM(DCM)-PBEh-3c level of theory. NCS·H2O⊂C3 corresponds to a hypothetical NCS bound in the H-pocket. The entry highlighted in gray corresponds to the reactant state used for subsequent calculations.*

| | $\Delta H$ [kcal mol$^{-1}$] | $-T\Delta S$ [kcal mol$^{-1}$] | $\Delta G$ [kcal mol$^{-1}$] |
|---|---|---|---|
| NCS⊂**C3**$_1$ | -5.0 | 1.7 | −3.3 |
| NCS⊂**C3**$_2$ | 0.0 | 0.0 | 0.0 |
| NCS·$H_2O$⊂**C3** | -4.7 | 8.2 | 3.4 |
| NCS·**1b**⊂**C3** | -20.1 | 16.6 | −3.5 |

### 6.2.2 Uncatalyzed reaction

We modeled the uncatalyzed chlorocycloetherification process based on the chlorolactonization mechanism proposed by Borhan and co-workers (Figure S64).[S45] The TS was identified using the NEB method and further characterized at the CPCM(DCM)-M06-2X/def2-TZVP//CPCM(DCM)-PBEh-3c level of theory, which was validated against CPCM(DCM)-DLPNO-CCSD(T)/def2-TZVP/CPCM(DCM)-PBEh-3c. The results show RMSE of 1.5 kcal mol$^{-1}$, validating the choice of M06-2X level of theory.

In this reaction, the substrates can approach each other in either *anti-* or *syn-* configurations, differing on the relative orientation of the forming bond relative to the double bond: *syn-* orientation occurs when both substitutes add to the same side of the bond, while *anti-* when on opposite sides. (Figure S65). Additionally, the *syn-TS* can involve a proton transfer (sy*nH*-TS) or proceed without it (*syn-*TS), leading to three potential TSs.

The *anti-* and *syn*H-TSs show similar activation energies at the CPCM(DCM)-M06-2X/def2-TZVP level of theory, with a slight preference for the *synH*-TS ($\Delta G^{\ddagger}$ = 32.9 and 31.7 kcal mol$^{-1}$, respectively, Figure S65 and Table S6). These values remain similar at the DLPNO-CCSD(T)/def2-TZVPP level of theory ($\Delta G^{\ddagger}$=34.2 and 34.4 kcal mol$^{-1}$, respectively), suggesting that both pathways are feasible (Table S6).

In *syn-* and *anti-* cases, the first addition step led to the formation of an intermediate oxonium cation (2b+) and a succinimide anion (Suc$^{-}$). Attempts to locate a TS connecting this intermediate and the corresponding product were unsuccessful. 1D scans indicated a barrierless reaction, leading us to conclude that the first step is rate limiting. The final product for the *synH*-TS pathway is the neutral ether product (2b) and the tautomer of succinimide (SucH), which can rearrange to form succinimide via a water-mediated proton transfer ($\Delta G^{\ddagger}$ = 8.0 kcal mol$^{-1}$; Figure S66; Table S7).

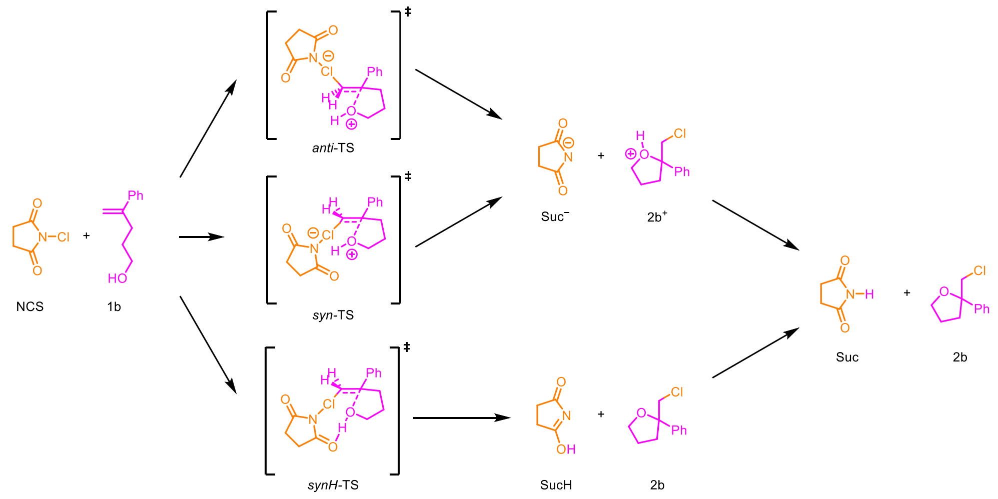


*Figure S64 The anti- and syn- transition state proposition based on the mechanism proposed by Borhan and co-workers.*[S45]

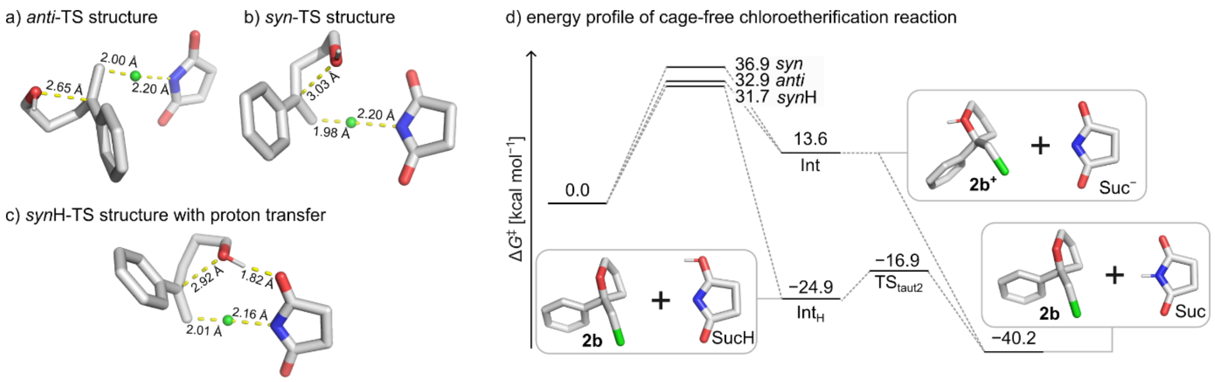


*Figure S65 (a-c) Possible transition state (TS) structures for the chloretherification reaction (a) anti-TS, (b) syn-TS, (c) syn-TS coupled to proton transfer (synH). (d) Energy profiles for each of these pathways computed at the CPCM(DCM)-M06-2X/def2-TZVP//CPCM(DCM)-PBEh-3c level of theory.*

*Table S6 Relative energies for the cage-free chloretherification reaction (kcal mol$^{-1}$) computed at two different levels of theory. In both cases, geometries and thermal contributions were computed at the CPCM(DCM)-PBEh-3c level of theory.*

| System | CPCM(DCM)-DLPNO-CCSD(T)/def2-TZVPP | | | | CPCM(DCM)-M06-2X/def2-TZVP | | | |
|---|---|---|---|---|---|---|---|---|
| | $\Delta E$ | $\Delta H$ | $-T\Delta S$ | $\Delta G$ | $\Delta E$ | $\Delta H$ | $-T\Delta S$ | $\Delta G$ |
| RS (**1b** + NCS) | 0 | 0.0 | 0.0 | 0.0 | 0 | 0.0 | 0.0 | 0.0 |
| *anti*-TS | 22.4 | 23.1 | 11.0 | 34.2 | 21.1 | 21.8 | 11.0 | 32.9 |
| *syn*-TS | 27.3 | 26.4 | 12.0 | 38.4 | 25.8 | 26.4 | 10.5 | 36.9 |
| *syn*H-TS | 22.2 | 22.6 | 11.8 | 34.4 | 19.4 | 19.9 | 11.8 | 31.7 |
| Int (**2b**$^+$ + Suc$^-$) | 10.4 | 11.8 | 1.5 | 13.4 | 10.6 | 12.0 | 1.5 | 13.6 |
| Int$_H$ (**2b** + SucH) | -28.5 | -27.1 | 1.3 | -25.7 | -27.6 | -26.2 | 1.3 | -24.9 |
| PS (**2b** + Suc) | -43.9 | -42.3 | 1.2 | -41.0 | -43.0 | -41.4 | 1.2 | -40.2 |

| | |
|---|---|
| MAE | 1.2 |
| RMSE | 1.5 |

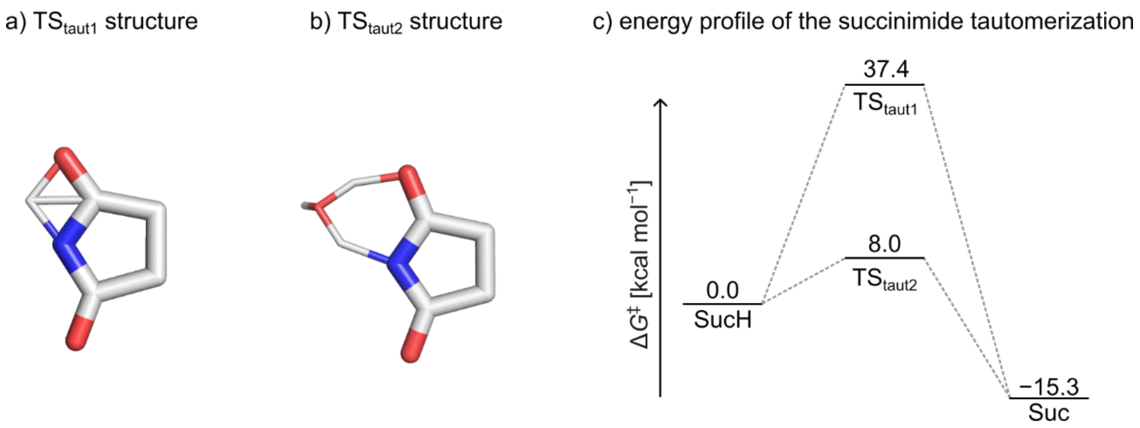


*Figure S66 Possible transition state (TS) structures for succinimide tautomerization via (a) direct proton transfer and (b) relayed proton transfer. (c) Energy profile computed at the CPCM(DCM)-M06-2X/def2-TZVP//CPCM(DCM)-PBEh-3c level of theory.*

*Table S7 Relative energies for the tautomerization of SucH to Suc, calculated at the CPCM(DCM)-M06-2X/def2-TZVP//CPCM(DCM)-PBEh-3c level of theory.*

| | $\Delta H$ [kcal mol$^{-1}$] | $-T\Delta S$ [kcal mol$^{-1}$] | $\Delta G$ [kcal mol$^{-1}$] |
|---|---|---|---|
| RS (SucH) | 0.0 | 0.0 | 0.0 |
| $TS_{taut1}$ | 37.3 | 0.1 | 37.4 |
| $TS_{taut2}$ | -1.6 | 9.6 | 8.0 |
| PS(Suc) | -15.2 | -0.1 | -15.3 |

### 6.2.3 Catalyzed reaction

The *anti*- and *syn*H-TSs were placed in the **C1** and **C3** cages and optimized with constraints on the forming and breaking bonds (N−Cl and Cl−C). The resulting “TS analogs” were then fully optimized to their respective transition states (Figure S67). For the **C3** cage, an additional TS was modeled with the oxygen of the hydroxyl group interacting with the C-H pocket to determine if this interaction could stabilize the TS (Figure S67e). In both cages, the *anti*-TS was preferred (Figure S67). While the computed activation barriers are consistently higher by about 4−5 kcal mol$^{-1}$ than the experimentally derived values (derived by converting the observed rates to energies using the Eyring equation), the observed trends in rate enhancement follow those from experiments. For **C1** and **C3** they correspond to $\Delta\Delta G^{\ddagger}_{comp} = -7.1$ and $-8.6$ kcal mol$^{-1}$ and $\Delta\Delta G^{\ddagger}_{exp} = -6.3$ and $-7.6$ kcal mol$^{-1}$, respectively). The calculations indicate that this stabilization is primarily enthalpic in nature ($\Delta\Delta H^{\ddagger} = -8.9$ kcal mol$^{-1}$, $T\Delta\Delta S^{\ddagger} = 1.8$ kcal mol$^{-1}$), due to a lowering of the LUMO energy (Table S9).

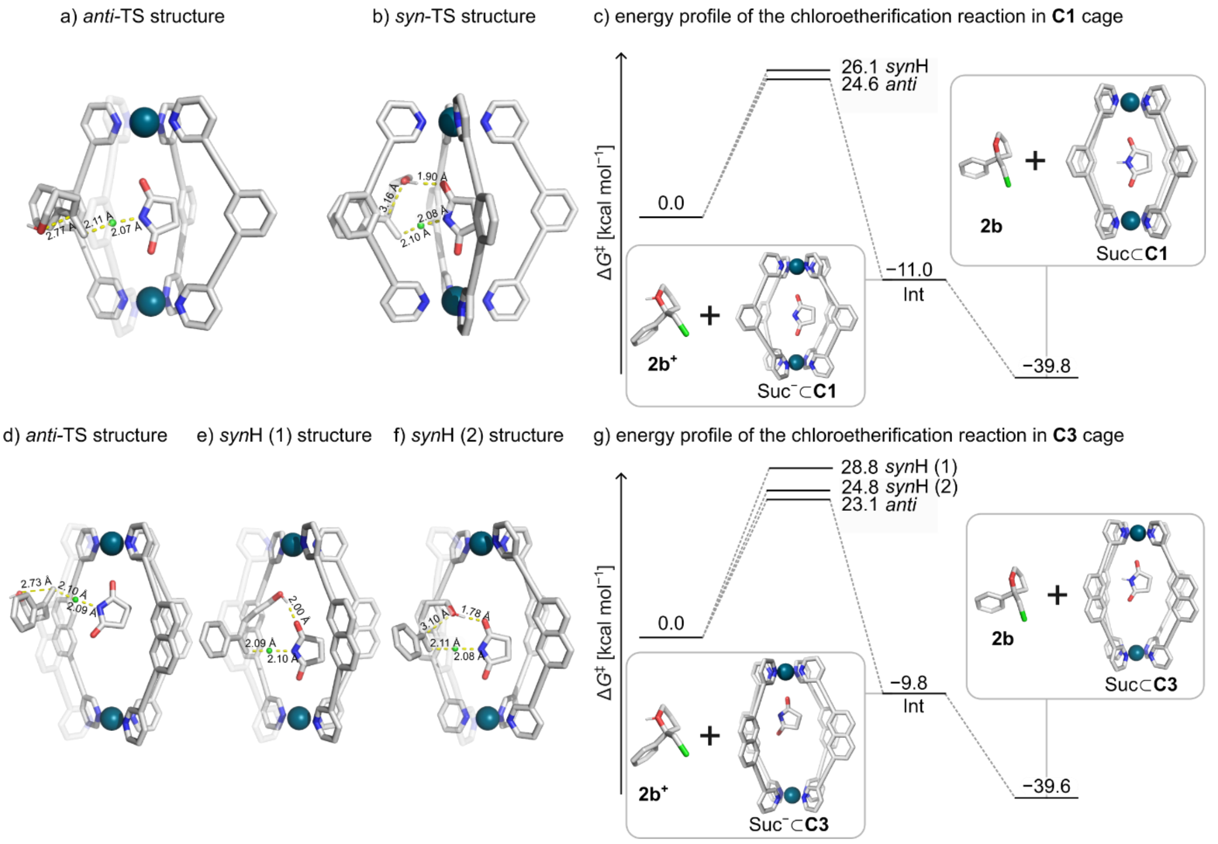


*Figure S67 (a-b) TS optimized structures for the chloretirification reaction in (a,b)* ***C1*** *and (d-f)* ***C3*** *cages in (a,d) anti-, and (b,e,f) syn- orientations. (c,g) Energy profile obtained at the CPCM(DCM)-M06-2X/def2-TZVP//CPCM(DCM)-PBEh-3c level of theory.*

*Table S8 Relative energies for the chloretirification reaction in* ***C1*** *and* ***C3*** *computed at the CPCM(DCM)-M06-2X/def2-TZVP//CPCM(DCM)-PBEh-3c level of theory. Energies in kcal mol$^{-1}$.*

| **C1** cage | Δ*H* | −*T*Δ*S* | Δ*G* | **C3** cage | Δ*H* | −*T*Δ*S* | Δ*G* |
|---|---|---|---|---|---|---|---|
| RS (**1b** + NCS⊂**C1**) | 0.0 | 0.0 | 0.0 | RS (**1b** + NCS⊂**C3**) | 0.0 | 0.0 | 0.0 |
| TS (*anti*⊂**C1**) | 11.0 | 13.6 | 24.6 | TS (*anti*⊂**C3**) | 8.1 | 15.0 | 23.1 |
| TS (*syn*H⊂**C1**) | 11.2 | 14.9 | 26.1 | TS (*syn*H(1)⊂**C3**) | 8.2 | 16.6 | 24.8 |
| | | | | TS (*syn*H(2)⊂**C3**) | 11.9 | 16.9 | 28.8 |
| Int (2b$^+$ + Suc$^-$⊂**C1**) | -12.6 | 1.5 | -11.0 | Int (**2b**$^+$ + Suc$^-$⊂**C3**) | -12.1 | 2.3 | -9.8 |
| PS (2b + Suc⊂**C1**) | -40.3 | 0.5 | -39.8 | PS (**2b** + Suc⊂**C3**) | -41.0 | 1.4 | -39.6 |

*Table S9 HOMO-LUMO gap between alcohol and NCS calculated at the CPCM(DCM)-M06-2X/def2-TZVP//CPCM(DCM)-PBEh-3c at the transition state geometries.*

| | Alcohol, **1b** (HOMO) | NCS<br>NCS⊂**C1**<br>NCS⊂**C3** (LUMO) | $E_{GAP}$ [eV] | Δ$E_{GAP}$ [eV] |
|---|---|---|---|---|
| Cage-free | −7.31 | −4.17 | 3.14 | |
| **C1** | −7.29 | −4.58 | 2.70 | −0.44 |
| **C3** | −7.31 | −4.62 | 2.70 | −0.44 |

## 6.3 α-methylstyrene chlorination

### 6.3.1 Uncatalyzed reaction

The chlorination of α-methylstyrene by NCS can proceed *via* either a stepwise or concerted mechanism (Figure S68**)**. In the stepwise pathway, NCS donates a chloride ion to the alkene, resulting in the formation of a carbocation intermediate, β-chlorocumene cation ($BCC^+$), and succinimide anion ($Suc^-$). In the next step, the carbocation transfers a proton to $Suc^-$, resulting in the final products, β-chlorocumene (BCC), and succinimide (Suc). Alternatively, the reaction may follow a concerted pathway, where chloride donation and proton transfer occur simultaneously, forming a succinimide tautomer.

**Stepwise mechanism**

To identify the first transition state for this pathway, we initially applied the NEB method at the CPCM(DCM)-D3BJ-PBE0/def2-SVP level of theory. This level of theory has been effective in locating TS for Diels-Alder and Michael addition reactions in $[Pd_2L_4]^{2+}$ cages.[S24,S44] However, our initial attempts to identify a TS were unsuccessful, resulting in a barrierless reaction.

We then performed a 1D scan by incrementally increasing the distance between the chloride and NCS nitrogen atoms (Figure S69a). The resulting energy profile confirmed a barrierless reaction, with a significant gradient change observed at $d_{Cl\text{-}N}$ = 2.2 Å. To evaluate the impact of the level of theory on these results, we also performed single-point energy calculations along this scan at the CPCM(DCM)-M06-2X/def2-TZVP and CPCM(DCM)-DLPNO-CCSD(T)/def2-TZVPP. These calculations revealed an electronic energy barrier ($\Delta E^{\ddagger}$) of 26.2 and 28.0 kcal $mol^{-1}$, respectively, at $d_{Cl\text{-}N}$ = 2.3 Å. Note that to simplify the comparison of the method, we focus only on the differences in the electronic energy barrier ($\Delta E^{\ddagger}$) rather than the Gibbs free energy barrier ($\Delta G^{\ddagger}$). Due to the high computational cost of these methods, we also tested three other approaches: CPCM(DCM)-D3BJ-PBE0/def2-TZVP, CPCM(DCM)-r2SCAN-3c, and CPCM(DCM)-PBEh-3c. Both CPCM(DCM)-PBEh-3c and CPCM(DCM)-D3BJ-PBE0/def-TZVP revealed a barrier at 2.3 Å, with energy barrier height of $\Delta E^{\ddagger}$=26.6 kcal $mol^{-1}$ and $\Delta E^{\ddagger}$=18.1 kcal $mol^{-1}$, respectively, while the latter one significantly underestimating barrier compare to the reference method (CPCM(DCM)-DLPNO-CCSD(T)/def2-TZVPP). These results suggest the level of theory not only affect energetics but also the shape of the potential energy surface (PES).

Based on these results, and considering computational efficiency, we use CPCM(DCM)-PBEh-3c for optimizations and thermochemical calculations. Using this level of theory, a 2D scan was performed, and the structure corresponding to the highest point on the energy profile was optimized for TS at the same level of theory. Single point energy of the TS was calculated at CPCM(DCM)-M06-2X/def2-TZVP level of theory, resulting in an energy barrier of $\Delta G^{\ddagger}$= 33.1 kcal $mol^{-1}$, which is 5 kcal $mol^{-1}$ higher than the experimental value. Attempt to locate the second TS, corresponding to the proton transfer process, using the CI-NEB method identified a TS that was 3.1 kcal $mol^{-1}$ higher in energy than the intermediate. However, frequency calculations revealed several low-frequency values (<100 $cm^{-1}$), which, upon optimization to TS converged to intermediate structure. A 1D scan suggested a

barrierless process. Consequently, we conclude that this step is either barrierless or associated with low activation energy.

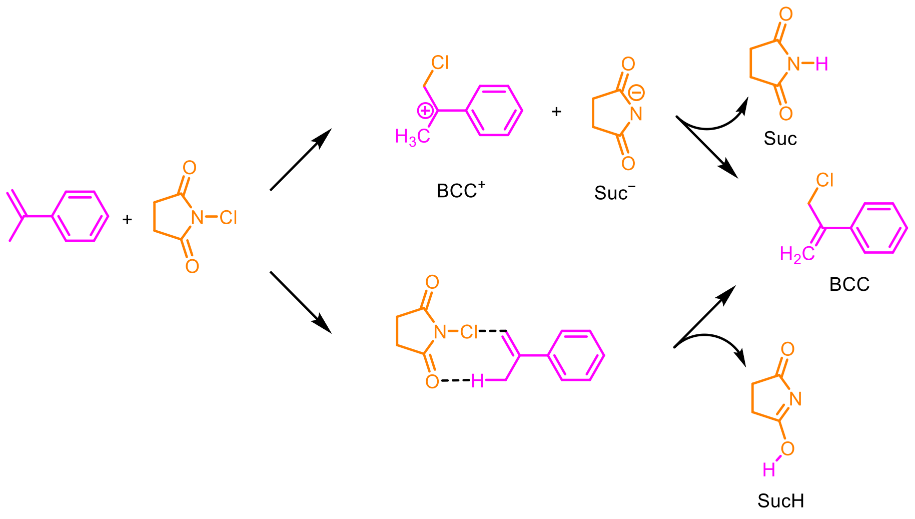


*Figure S68 Proposed reaction mechanism of α-methylstyrene chlorination via a stepwise (top) or concerted (bottom) mechanism.*

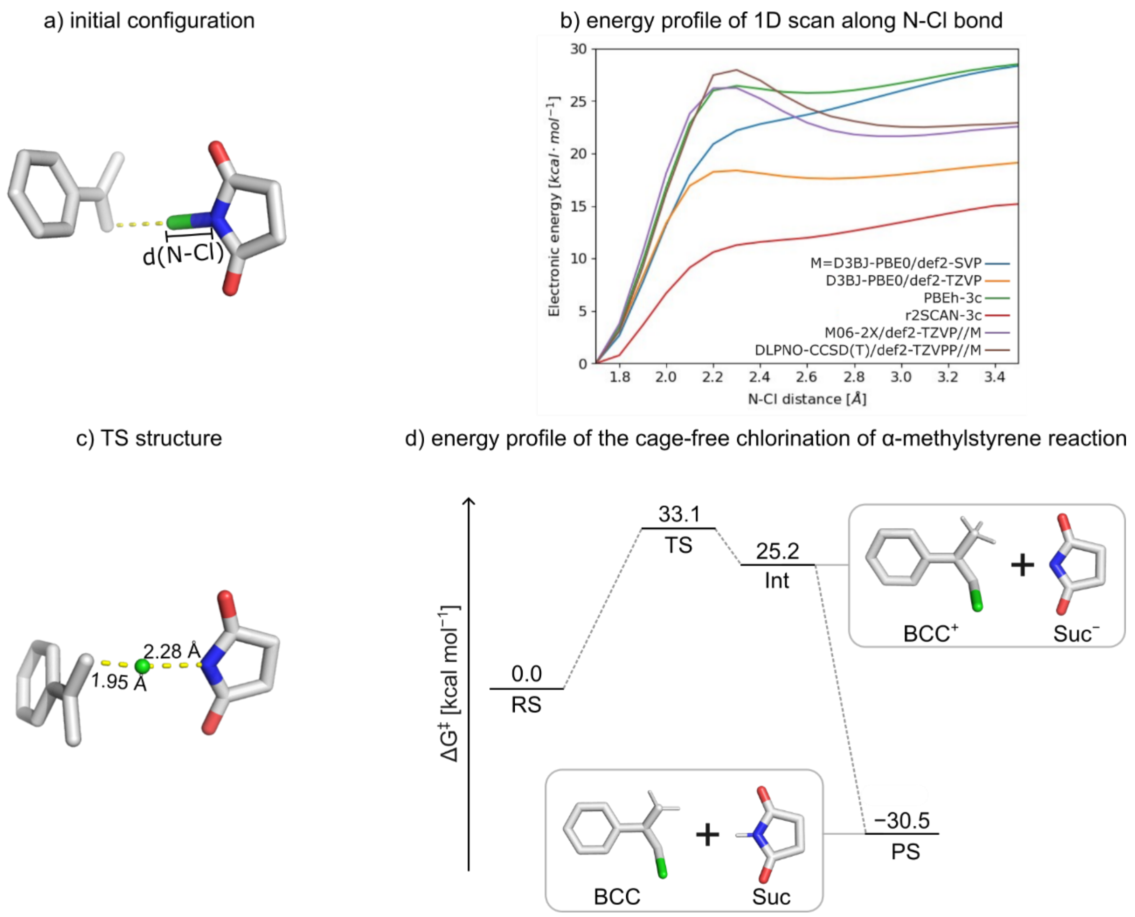


*Figure S69 (a) Initial configuration of α-methylstyrene and NCS with indicated nitrogen chloride bond used for the 1D scan. (b) Energy profile of 1D scan using different levels of theory. Note that initial methods D3BJ-PBE0/def2-SVP and r2SCAN-3c show barrierless reactions. (c) Transition state structure. (d) Energy profile of cage-free α-methylstyrene chlorination.*

## Concerted mechanism

To explore the possibility of a concerted mechanism in the α-methylstyrene chlorination reaction, we conducted a 2D energy scan varying both the N−Cl and O−H distances. This was done using the TS structure obtained from the stepwise mechanism as a starting point (Figure S70a,b, Table S10). Single-point energy calculations were conducted at the CPCM(DCM)-M06-2X/def2-TZVP level of theory (Figure S70c). The resulting energy profile revealed a broad saddle point at $d_{N-Cl}$ = 2.3Å, with little change in the O−H distance. These findings did not provide evidence for a concerted mechanism.

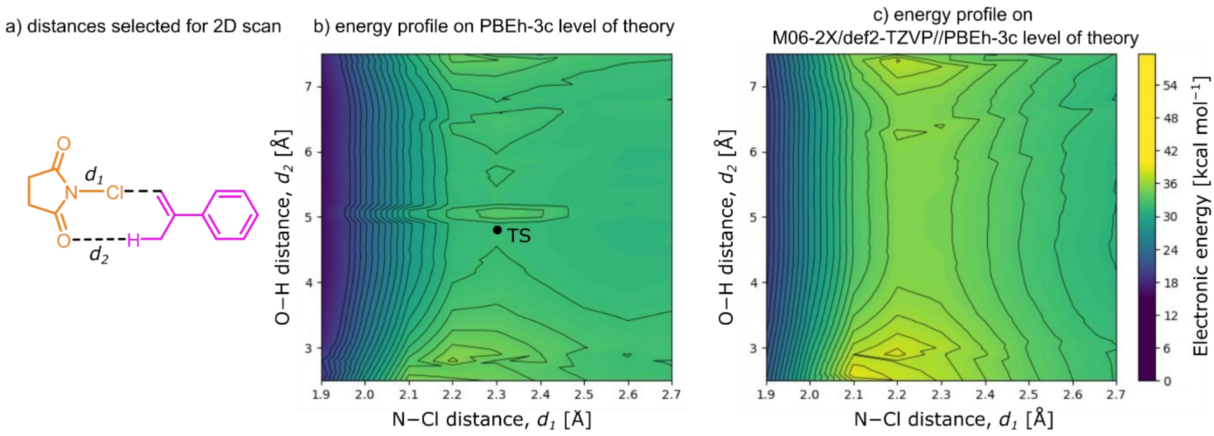


*Figure S70 2D scan along the selected distances $d_1$ and $d_2$ computed at two different levels of theory (a) CPCM(DCM)-PBEh-3c and (b) CPCM(DCM)-M06-2X/def2-TZVP//CPCM(DCM)-PBEh-3c.*

*Table S10 The QM analysis of cage-free α-methylstyrene chlorination. Geometries optimizations and thermochemical contributions were calculated on the CPCM(DCM)-PBEh-3c level of theory. Energies in kcal mol$^{-1}$.*

| | CPCM(DCM)-DLPNO-CCSD(T)/def2-TZVPP | CPCM(DCM)-M06-2X/def2-TZVP | | | |
|---|---|---|---|---|---|
| | $\Delta E$ | $\Delta E$ | $\Delta H$ | $-T\Delta S$ | $\Delta G$ |
| RS (**1b** + NCS) | 0.0 | 0.0 | 0.0 | 0.0 | 0.0 |
| TS | 24.3 | 22.9 | 23.2 | 9.8 | 33.1 |
| Int (BCC+ + Suc⁻) | 26.2 | 25.8 | 25.9 | -0.7 | 25.2 |
| PS (BCC + Suc) | -32.5 | -31.5 | -30.4 | -0.1 | -30.5 |
| | MAE | 0.7 | | | |
| | RMSE | 0.9 | | | |

### 6.3.2 Catalyzed reaction

The transition state obtained from the cage-free reaction was placed inside the **C1** and **C3** cages and initially optimized with constraints on breaking N-Cl and forming Cl-C bonds. These transition state guesses were then fully optimized without constraints. The energy barriers for both reactions were significantly lower than those of the cage-free reaction ($\Delta G^{\ddagger}$ = 25.3 kcal/mol for **C1**, Figure S71, Error! Reference source not found.and 25.1 kcal/mol for **C3**, Figure S72, Table S11).

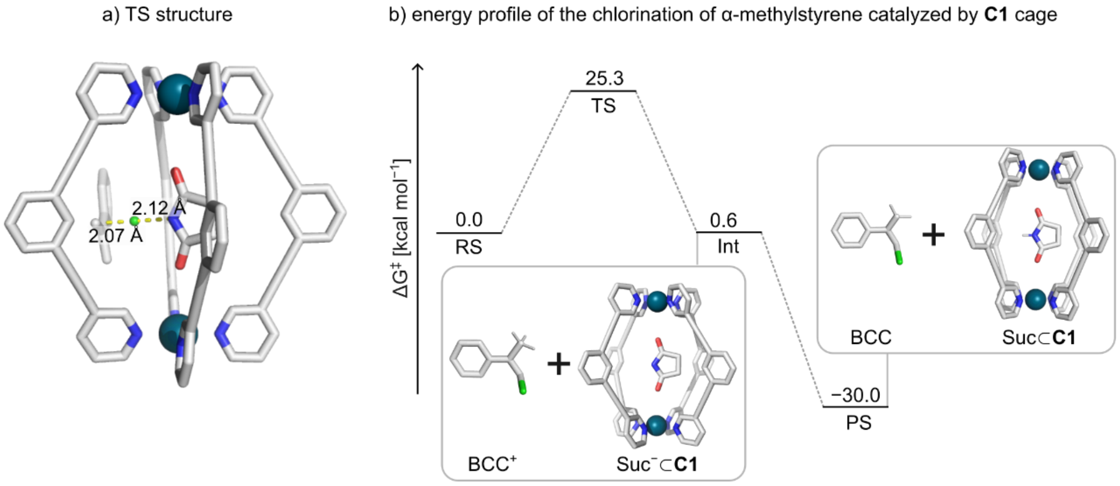


*Figure S71 α-methylstyrene chlorination reaction in* ***C1****. (a) TS structures. (b) Energy profile at CPCM(DCM)-M06-2X/def2-TZVP//CPCM(DCM)-PBEh-3c level of theory.*

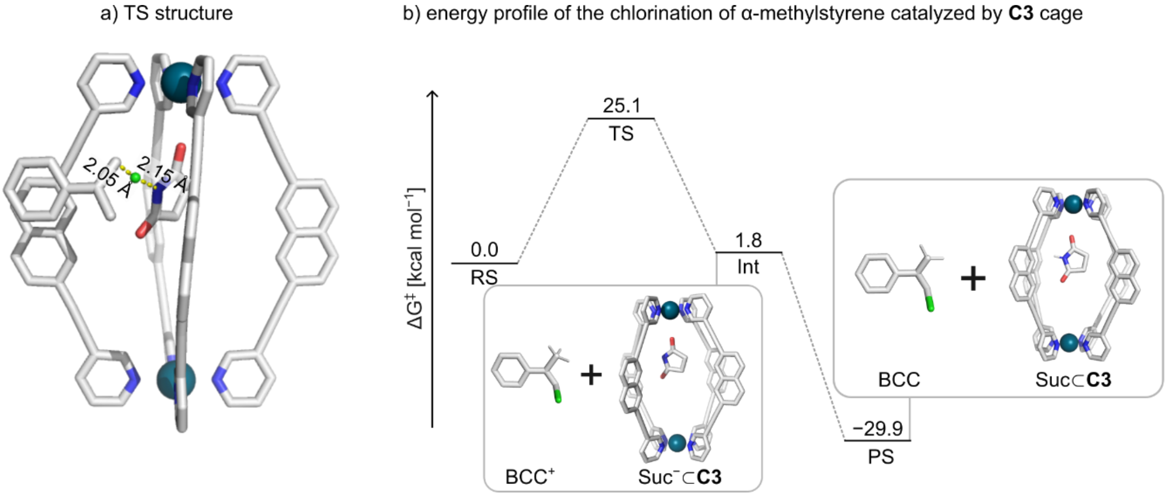


*Figure S72 α-methylstyrene chlorination reaction in* ***C3****. (a) TS structures. (b) Energy profile at CPCM(DCM)-M06-2X/def2-TZVP//CPCM(DCM)-PBEh-3c level of theory.*

*Table S11 The QM analysis of α-methylstyrene chlorination reaction in* ***C3****. Calculations on CPCM(DCM)-M06-2X/def2-TZVP//CPCM(DCM)-PBEh-3c level of theory. Energies in kcal mol$^{-1}$.*

| Cage **C1** | ΔH | −TΔS | ΔG | Cage **C3** | ΔH | −TΔS | ΔG |
|---|---|---|---|---|---|---|---|
| RS (**AMS** + NCS⊂**C1**) | 0.0 | 0.0 | 0.0 | RS (**AMS** + NCS⊂**C3**) | 0.0 | 0.0 | 0.0 |
| TS⊂C1 | 12.1 | 13.2 | 25.3 | TS⊂**C3** | 11.2 | 13.9 | 25.1 |
| Int(BCC+ + Suc−⊂**C1**) | 1.3 | -0.7 | 0.6 | Int(BCC+ + Suc−⊂**C3**) | 1.8 | 0.1 | 1.8 |
| PS(BCC+ Suc⊂**C1**) | -29.2 | -0.8 | -30.0 | PS(BCC+ Suc⊂**C3**) | -30.0 | 0.1 | -29.9 |

*Table S12 HOMO-LUMO gap between α-methylstyrene and NCS calculated at the CPCM(DCM)-M06-2X/def2-TZVP//CPCM(DCM)-PBEh-3c at the transition state geometries.*

| | α-methylstyrene (HOMO) | NCS<br>NCS⊂**C1**<br>NCS⊂**C3** (LUMO) | $E_{GAP}$ [eV] | $\Delta E_{GAP}$ [eV] |
|---|---|---|---|---|
| Uncat | −7.35 | −4.81 | 2.53 | |
| **C1** | −7.39 | −4.91 | 2.48 | −0.05 |
| **C3** | −7.47 | −5.00 | 2.46 | −0.07 |

### 6.3.3 Fragment energy decomposition analysis

To understand the origin of catalysis in **C1** and **C3**, we employed a fragment-based energy decomposition analysis inspired by QM/MM study that analyze the contribution to transition state stabilization in chorismite mutase.[S46] This approach involved partitioning the already optimized TS and reactant complex (RC) structures into various fragments. The RC was obtained by optimizing the end structure obtained by following the intrinsic reactant coordinate (IRC) from TS to RS. Each fragment was generated by removing the corresponding atoms using PyMOL and capping the remaining fragments with hydrogen atoms. We truncated the catalyst into the following fragments: single $[Pd(Py)_4]^{2+}$ site (**F1**), which contribute with the C-H binding pocket, two distinct $[Pd(Py)_4]^{2+}$ sites retaining the distance between them (**F2**) and a half-cage (**F3**) as shown in Figure S73a,b. For the **C1** cage, the two single $[Pd(Py)_4]^{2+}$ sites and two half-cage fragments were also considered separately (denoted as fragments **F1** and **F1b**, and **F3** and **F3b**), as they provided different stabilization effects. In **C3** the distance between the two ends means that only one $[Pd(Py)_4]^{2+}$ site interacts with NCS. Only **F1** and **F3**, which provided greater stabilization, are reported in the main text (Figure S73c,d**)**.

Second order Natural Bond Orbital (NBO) analysis for the **F1** fragments revealed stronger hydrogen bonding between NCS and **F1** in TS⊂**C3** cage (4.2 kcal mol$^{-1}$) than TS⊂**C1** (2.3 kcal mol$^{-1}$; Table S13), which is in line with observed hydrogen bond distances (2.41±0.07 Å and 2.56 ±0.03 Å for **C3** and **C1**, respectively, Figure S66a,b). In the **F2** fragment in TS⊂**C1** geometry, as expected from distance analysis, the second $[Pd(Py)_4]^{2+}$ site contribute little to the stabilization energy compared to **F1** (0.9 kcal mol$^{-1}$). For **C3**, the additional site offers no contribution to stabilization energy.

*Table S13 Relevant second-order perturbation theory values of NBO analysis. Calculations were carried out at the CPCM(DCM)-M06-2X/def2-TZVP level of theory.*

| Donor (L) NBO | Acceptor (NL) NBO | E(2) [kcal mol$^{-1}$] | E(NL)-E(L) [a.u.] | F(L,NL) [a.u.] |
|---|---|---|---|---|
| **F1** in the geometry of TS⊂**C1** | | | | |
| 52. LP ( 1) O 4 | 211. BD*( 1) C 38- H 42 | 0.18 | 1.11 | 0.013 |
| 53. LP ( 2) O 4 | 211. BD*( 1) C 38- H 42 | 0.52 | 0.79 | 0.018 |
| 52. LP ( 1) O 4 | 225. BD*( 1) C 49- H 53 | 0.19 | 1.11 | 0.013 |
| 53. LP ( 2) O 4 | 225. BD*( 1) C 49- H 53 | 0.57 | 0.79 | 0.019 |
| 52. LP ( 1) O 4 | 239. BD*( 1) C 60- H 64 | 0.27 | 1.11 | 0.016 |
| 53. LP ( 2) O 4 | 239. BD*( 1) C 60- H 64 | 0.19 | 0.79 | 0.011 |
| 52. LP ( 1) O 4 | 253. BD*( 1) C 71- H 75 | 0.24 | 1.11 | 0.014 |
| 53. LP ( 2) O 4 | 253. BD*( 1) C 71- H 75 | 0.14 | 0.79 | 0.009 |
| **F1** in the geometry of TS⊂**C3** | | | | |
| 57. LP ( 1) O 6 | 211. BD*( 1) C 39- H 43 | 0.12 | 1.12 | 0.010 |
| 58. LP ( 2) O 6 | 211. BD*( 1) C 39- H 43 | 0.26 | 0.79 | 0.013 |
| 57. LP ( 1) O 6 | 225. BD*( 1) C 50- H 54 | 0.60 | 1.12 | 0.023 |
| 58. LP ( 2) O 6 | 225. BD*( 1) C 50- H 54 | 1.00 | 0.80 | 0.025 |
| 57. LP ( 1) O 6 | 239. BD*( 1) C 61- H 65 | 0.17 | 1.12 | 0.012 |
| 58. LP ( 2) O 6 | 239. BD*( 1) C 61- H 65 | 0.41 | 0.79 | 0.016 |
| 57. LP ( 1) O 6 | 253. BD*( 1) C 72- H 76 | 0.66 | 1.12 | 0.024 |
| 58. LP ( 2) O 6 | 253. BD*( 1) C 72- H 76 | 0.99 | 0.80 | 0.025 |
| **F2** in the geometry of TS⊂**C1** | | | | |
| 80. LP ( 1) O 4 | 319. BD*( 1) C 45- H 53 | 0.18 | 1.11 | 0.012 |
| 81. LP ( 2) O 4 | 319. BD*( 1) C 45- H 53 | 0.51 | 0.79 | 0.018 |
| 80. LP ( 1) O 4 | 347. BD*( 1) C 67- H 75 | 0.19 | 1.12 | 0.013 |
| 81. LP ( 2) O 4 | 347. BD*( 1) C 67- H 75 | 0.55 | 0.79 | 0.019 |
| 80. LP ( 1) O 4 | 375. BD*( 1) C 89- H 97 | 0.27 | 1.11 | 0.015 |
| 81. LP ( 2) O 4 | 375. BD*( 1) C 89- H 97 | 0.19 | 0.79 | 0.011 |
| 80. LP ( 1) O 4 | 403. BD*( 1) C111- H119 | 0.23 | 1.11 | 0.014 |
| 81. LP ( 2) O 4 | 403. BD*( 1) C111- H119 | 0.14 | 0.79 | 0.009 |
| 85. LP ( 1) O 6 | 365. BD*( 1) C 84- H 93 | 0.21 | 1.11 | 0.014 |
| 86. LP ( 2) O 6 | 337. BD*( 1) C 62- H 71 | 0.29 | 0.79 | 0.014 |

| | | | | |
|---|---|---|---|---|
| 85. LP ( 1) O 6 | 393. BD*( 1) C106- H115 | 0.19 | 1.11 | 0.013 |
| 86. LP ( 2) O 6 | 309. BD*( 1) C 40- H 49 | 0.21 | 0.79 | 0.011 |
| **F2** in the geometry of TS⊂**C3** | | | | |
| 85. LP ( 1) O 6 | 305. BD*( 1) C 40- H 51 | 0.12 | 1.12 | 0.01 |
| 86. LP ( 2) O 6 | 305. BD*( 1) C 40- H 51 | 0.26 | 0.8 | 0.013 |
| 86. LP ( 2) O 6 | 333. BD*( 1) C 62- H 73 | 0.99 | 0.8 | 0.025 |
| 85. LP ( 1) O 6 | 333. BD*( 1) C 62- H 73 | 0.6 | 1.12 | 0.023 |
| 85. LP ( 1) O 6 | 361. BD*( 1) C 84- H 95 | 0.17 | 1.12 | 0.012 |
| 86. LP ( 2) O 6 | 361. BD*( 1) C 84- H 95 | 0.41 | 0.79 | 0.016 |
| 85. LP ( 1) O 6 | 389. BD*( 1) C106- H117 | 0.65 | 1.12 | 0.024 |
| 86. LP ( 2) O 6 | 389. BD*( 1) C106- H117 | 0.98 | 0.80 | 0.025 |

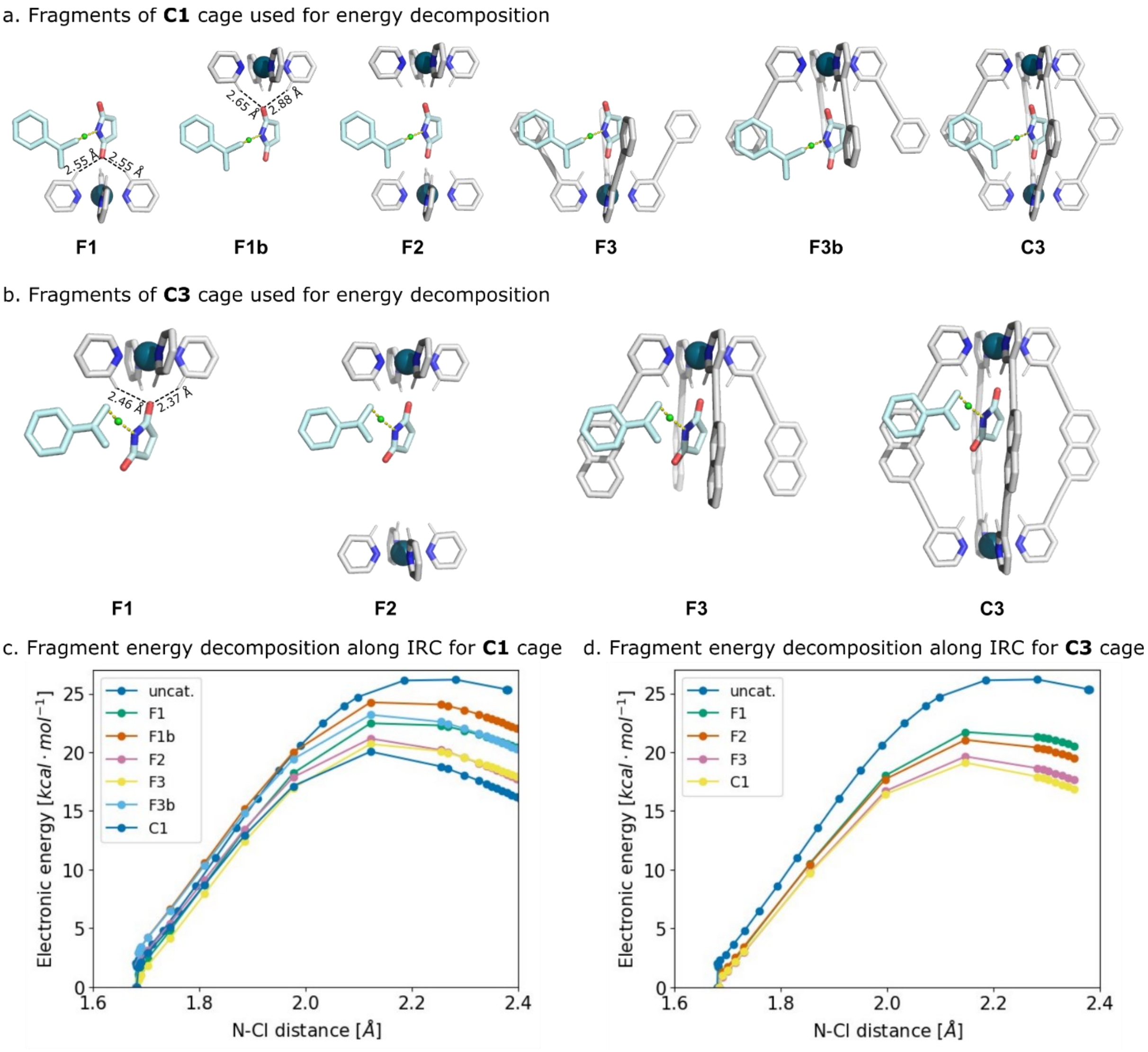


*Figure S73 Fragment energy decomposition analysis. Fragments used for analysis in C1 (a) and **C3** cages (b). (c-d) Relative electronic energy for each fragments along the IRC pathway (projected on N-Cl distance) for (c) **C1**, and (d) **C3** cages.*

*Table S14 Single point calculation and thermal contributions for the systems studied in this work. All energies in hartrees.*

| | CPCM(DCM)-PBE0-D3BJ/def2-SVP | | | | CPCM(DCM)-M06-2X/def2-TZVP | CPCM(DCM)-DLPNO-CCSD(T)/def2-TZVPP |
|---|---|---|---|---|---|---|
| | E | H-E | G-E | Im. freq. [cm$^{-1}$] | E | E |
| water | -76.2678 | 0.0257 | 0.0072 | | -76.4359 | |
| NCS | -818.9768 | 0.0927 | 0.0564 | | -820.2381 | -819.2017 |
| Suc− | -359.4281 | 0.0881 | 0.0550 | | -360.2019 | -359.6360 |
| SucH | -359.8938 | 0.1016 | 0.0681 | | -360.6612 | -360.1016 |
| Suc | -359.9224 | 0.1019 | 0.0682 | | -360.6857 | -360.1261 |
| TS_taut1 | -359.8277 | 0.0962 | 0.0629 | -2063.01 | -360.5964 | |
| TS_taut2 | -436.1643 | 0.1245 | 0.0878 | -723.84 | -437.0969 | |
| Reaction 1 | | | | | | |
| Alcohol 1 | -501.7041 | 0.2425 | 0.1968 | | -502.7750 | -501.9181 |
| anti-TS | -1320.6498 | 0.3363 | 0.2720 | -226.42 | -1322.9793 | -1321.0841 |
| syn-TS | -1320.6414 | 0.3361 | 0.2709 | -253.82 | -1322.9719 | -1321.0762 |
| synH-TS | -1320.6504 | 0.3359 | 0.2727 | -284.66 | -1322.9821 | -1321.0844 |
| ether cation | -961.2308 | 0.2493 | 0.2030 | | -962.7942 | -961.4672 |
| ether | -960.8354 | 0.2359 | 0.1895 | | -962.3959 | -961.0636 |
| NCS and derivatives in cages | | | | | | |
| NCS⊂**C1** | -4581.4834 | 1.2573 | 1.0725 | | -4590.2721 | |
| Suc−⊂**C1** | -4121.9766 | 1.2527 | 1.0710 | | -4130.2751 | |
| Suc⊂**C1** | -4122.4271 | 1.2662 | 1.0829 | -7.92 | -4130.7176 | |
| NCS⊂**C3**$_1$ | -5194.7152 | 1.4604 | 1.2563 | | -5204.8300 | |
| NCS⊂**C3**$_2$ | -5194.7134 | 1.4605 | 1.2536 | -13.97 | -5204.8222 | |
| NCS.H2O⊂**C3** (4) | -5270.9933 | 1.4891 | 1.2767 | -2.53 | -5281.2685 | |
| NCS.ROH⊂**C3** (4) | -5696.4519 | 1.7055 | 1.4794 | | -5707.6316 | |
| Suc$^-$⊂**C3** | -4735.2034 | 1.4560 | 1.2533 | | -4744.8244 | |
| Suc⊂**C3** | -4735.6569 | 1.4697 | 1.2657 | -2.13, -18.37 | -4745.2691 | |
| Reaction 1 in **C1** | | | | | | |
| TS(anti⊂**C1**) | -5083.1806 | 1.5016 | 1.2928 | -9.65, -315.96 | -5093.0313 | |
| TS(synH⊂**C1**) | -5083.1750 | 1.5008 | 1.2941 | -317.89 | -5093.0301 | |
| Reaction 1 in **C3** | | | | | | |
| TS(anti⊂**C3**) | | 1.7038 | 1.4752 | -4.11, -298.86 | -5707.5850 | |
| TS(synH1⊂**C3**) | -5696.4128 | 1.7048 | 1.4786 | -338.54 | -5707.5858 | |
| TS(synH2⊂**C3**) | -5696.4069 | 1.7045 | 1.4788 | -349.05 | -5707.5797 | |
| Reaction 2 | | | | | | |
| AMS | -348.1915 | 0.1751 | 0.1370 | | -348.9330 | -348.3087 |
| TS | -1167.1323 | 0.2683 | 0.2096 | -159.66 | -1169.1347 | -1167.4716 |
| BCC$^+$ | -807.6901 | 0.1797 | 0.1374 | | -808.9280 | -807.8327 |
| BCC | -807.2918 | 0.1676 | 0.1267 | | -808.5356 | -807.4361 |
| Reaction 2 in cages | | | | | | |
| TS⊂**C1** | -4929.6631 | 1.4329 | 1.2311 | -310.92 | -4939.1864 | |
| TS⊂**C3** | -5542.8904 | 1.6369 | 1.4142 | -3.63, -5.48, -242.39 | -5553.7382 | |

# 7. Reference


S1. August, D. P.; Nichol, G. S.; Lusby, P. J. Maximizing Coordination Capsule–Guest Polar Interactions in Apolar Solvents Reveals Significant Binding. *Angew. Chem. Int. Ed.* **2016**, *55* (48), 15022–15026.

S2. Wang, J.; Young, T. A.; Duarte, F.; Lusby, P. J. Synergistic Noncovalent Catalysis Facilitates Base-Free Michael Addition. *J. Am. Chem. Soc.* **2020**, *142* (41), 17743-17750.

S3. O'Connor, H. M.; Tipping, W. J.; Vallejo, J.; Nichol, G. S.; Faulds, K.; Graham, D.; Brechin, E. K.; Lusby, P. J. Utilizing Raman Spectroscopy as a Tool for Solid- and Solution-Phase Analysis of Metalloorganic Cage Host-Guest Complexes. *Inorg. Chem.* **2023**, *62* (5), 1827-1832.

S4. Nicolai, S.; Erard, S.; Gonzalez, D. F.; Waser, J. Pd-catalyzed intramolecular oxyalkynylation of alkenes with hypervalent iodine. *Org. Lett.* **2010**, *12* (2), 384-387.

S5. Whitehead, D. C.; Yousefi, R.; Jaganathan, A.; Borhan, B. An organocatalytic asymmetric chlorolactonization. *J. Am. Chem. Soc.* **2010**, *132* (10), 3298-3300.

S6. Scaringi, S.; Mazet, C. Kinetically Controlled Stereoselective Access to Branched 1,3-Dienes by Ru-Catalyzed Remote Conjugative Isomerization. *ACS Catalysis* **2021**, *11* (13), 7970-7977.

S7. Denmark, S. E.; Burk, M. T. Enantioselective bromocycloetherification by Lewis base/chiral Bronsted acid cooperative catalysis. *Org. Lett.* **2012**, *14* (1), 256-259.

S8. Jing, C.; Jones, B. T.; Adams, R. J.; Bower, J. F. Cyclopropane-Fused N-Heterocycles via Aza-Heck-Triggered C(sp$^3$)–H Functionalization Cascades. *J. Am. Chem. Soc.* **2022**, *144* (37), 16749–16754.

S9. Sirinimal, H. S.; Hebert, S. P.; Samala, G.; Chen, H.; Rosenhauer, G. J.; Schlegel, H. B.; Stockdill, J. L. Synthetic and Computational Study of Tin-Free Reductive Tandem Cyclizations of Neutral Aminyl Radicals. *Org. Lett.* **2018**, *20* (20), 6340–6344.

S10. O'Duill, M. L.; Matsuura, R.; Wang, Y.; Turnbull, J. L.; Gurak Jr, J. A.; Gao, D. W.; Lu, G.; Liu, P.; Engle, O. M. Tridentate Directing Groups Stabilize 6-Membered Palladacycles in Catalytic Alkene Hydrofunctionalization. *J. Am. Chem. Soc.* **2017**, *139* (44), 15576–15579.

S11. Ashtekar, K. D.; Vetticatt, M.; Yousefi, R.; Jackson, J. E.; Borhan, B. Nucleophile-Assisted Alkene Activation: Olefins Alone Are Often Incompetent. *J. Am. Chem. Soc.* **2016**, *138* (26), 8114-8119.

S12. Brücher, O.; Hartung, J. Oxidative chlorination of 4-pentenols and other functionalized hydrocarbons. *Tetrahedron* **2014**, *70* (43), 7950-7961.

S13. Denmark, S. E.; Ryabchuk, P.; Burk, M. T.; Gilbert, B. B. Toward Catalytic, Enantioselective Chlorolactonization of 1,2-Disubstituted Styrenyl Carboxylic Acids. *J. Am. Chem. Soc.* **2016**, *81* (21), 10411-10423.

S14. Satoh, T.; Ogino, Y.; Ando, K. Direct alkenylation of arylamines at the ortho-position with magnesium alkylidene carbenoids and some theoretical studies of the reactions. *Tetrahedron* **2005**, *61* (43), 10262-10276.

S15. Younes, S. H. H.; Tieves, F.; Lan, D.; Wang, Y.; Süss, P.; Brundiek, H.; Wever, R.; Hollmann, F. Chemoenzymatic Halocyclization of γ,δ-Unsaturated Carboxylic Acids and Alcohols. *ChemSusChem* **2020**, *13*, 97 – 101.

S16. Taillez, B.; Bertrand, M. P.; Surzur, J. Intramolecular addition of alkoxyl radicals. Part 4. Reductive cyclisation of olefinic hydroperoxides. *J. Chem. Soc., Perkin Trans. 2*, **1983**, *5*, 547-553.

S17. Marti-Centelles, V.; Spicer, R. L.; Lusby, P. J. Non-covalent allosteric regulation of capsule catalysis. *Chem. Sci.* **2020**, *11* (12), 3236-3240.

S18. Marti-Centelles, V.; Andrew L. L.; Paul J. L. High Activity and Efficient Turnover by a Simple, Self-Assembled "Artificial Diels−Alderase". *J. Am. Chem. Soc.* **2018**, *140* (8), 2862−2868.
S19. Swamy, P.; Kumar, M. A.; Reddy, M. M.; Naresh, M.; Srujana, K.; Narender, N. The vicinal functionalization of olefins: a facile route to the direct synthesis of β-chlorohydrins and β-chloroethers. *RSC Adv.* **2014**, *4* (50), 26288-26294.
S20. Xu, B.; Tambar, U. K. Remote Allylation of Unactivated C(sp3)–H Bonds Triggered by Photogenerated Amidyl Radicals. *ACS Catal.* **2019**, *9* (5), 4627-4631.
S21. Piskorz, T. K.; Lee, B.; Zhan, S.; Duarte, F. Metallicious: Automated Force-Field Parameterization of Covalently Bound Metals for Supramolecular Structures. *J. Chem. Theory Comput.* **2024**, *20* (20), 9060–9071.
S22. Wang, J.; Wang, W.; Kollman, P. A.; Case, D. A. Automatic Atom Type and Bond Type Perception in Molecular Mechanical Calculations. *J. Mol. Graph. Model.* **2006**, *25* (2), 247–260.
S23. Li, Z.; Song, L. F.; Li, P.; Merz, K. M. Systematic Parametrization of Divalent Metal Ions for the OPC3, OPC, TIP3P-FB, and TIP4P-FB Water Models. *J. Chem. Theory Comput.* **2020**, *16* (7), 4429–4442.
S24. Boaler, P. J.; Piskorz, T. K.; Bickerton, L. E.; Wang, J.; Duarte, F.; Lloyd-Jones, G. C.; Lusby, P. J. Origins of High-Activity Cage-Catalyzed Michael Addition. *J. Am. Chem. Soc.* **2024**, *146* (28), 19317–19326.
S25. Abraham, M. J.; Murtola, T.; Schulz, R.; Pall, S.; Smith, J. C.; Hess, B.; Lindah, E. Gromacs: High Performance Molecular Simulations through Multi-Level Parallelism from Laptops to Supercomputers. *SoftwareX* **2015**, *1–2*, 19–25.
S26. Van Der Spoel, D.; Lindahl, E.; Hess, B.; Groenhof, G.; Mark, A. E.; Berendsen, H. J. C. GROMACS: Fast, Flexible, and Free. *J. Comput. Chem.* **2005**, *26* (16), 1701–1718.
S27. van der Spoel, D.; van Maaren, P. J.; Caleman, C. GROMACS Molecule & Liquid Database. *Bioinformatics* **2012**, *28* (5), 752–753.
S28. Izadi, S.; Anandakrishnan, R.; Onufriev, A. V. Building Water Models: A Different Approach. *J. Phys. Chem. Lett.* **2014**, *5*, 3853–3871.
S29. Bussi, G.; Donadio, D.; Parrinello, M. Canonical Sampling through Velocity Rescaling. *J. Chem. Phys.* **2007**, *126* (2007), 014101.
S30. Bernetti, M.; Bussi, G. Pressure Control Using Stochastic Cell Rescaling. *J. Chem. Phys.* **2020**, *153* (11), 114107.
S31. Darden, T.; York, D.; Pedersen, L. Particle Mesh Ewald: An N -Log(N) Method for Ewald Sums in Large Systems. *J. Chern. Phys.* **1993**, *98* (12), 10089–10092.
S32. Essmann, U.; Perera, L.; Berkowitz, M. L.; Darden, T.; Lee, H.; Pedersen, L. G. A Smooth Particle Mesh Ewald Method. *J. Chem. Phys.* **1995**, *103* (19), 8577–8593.
S33. Michaud-Agrawal, N.; Denning, E. J.; Woolf, T. B.; Beckstein, O. MDAnalysis: A Toolkit for the Analysis of Molecular Dynamics Simulations. *J. Comput. Chem.* **2011**, *32* (10), 2319–2327.
S34. Gowers, R. J.; Linke, M.; Barnoud, J.; Reddy, T. J. E.; Melo, M. N.; Seyler, S. L.; Domański, J.; Dotson, D. L.; Buchoux, S.; Kenney, I. M.; et al. MDAnalysis: A Python Package for the Rapid Analysis of Molecular Dynamics Simulations. In *Proceedings of the 15th Python in Science Conference*; Benthall, S., Rostrup, S., Eds.; 2016; pp 98–105.
S35. Hub, J. S.; De Groot, B. L.; Van Der Spoel, D.; Groot, B. L. De; Spoel, D. Van Der. G_wham - Free Weighted Histogram Analysis Implementation Including Robust Error and Autocorrelation Estimates. *J. Chem. Theory Comput.* **2010**, *6* (12), 3713–3720.

S36. Neumann, R. M. Entropic Approach to Brownian Movement. *Am. J. Phys.* **1980**, *48* (5), 354–357.
S37. Neese, F.; Wennmohs, F.; Becker, U.; Riplinger, C. The ORCA Quantum Chemistry Program Package. *J. Chem. Phys.* **2020**, *152* (22).
S38. Neese, F. Software Update: The ORCA Program System—Version 5.0. *Wiley Interdiscip. Rev. Comput. Mol. Sci.* **2022**, *12* (5), 1–15.
S39. Grimme, S.; Brandenburg, J. G.; Bannwarth, C.; Hansen, A. Consistent Structures and Interactions by Density Functional Theory with Small Atomic Orbital Basis Sets. *J. Chem. Phys.* **2015**, *143* (5), 54107.
S40. Grimme, S. Supramolecular Binding Thermodynamics by Dispersion-Corrected Density Functional Theory. *Chem. Eur. J.* **2012**, *18* (32), 9955–9964.
S41. Zhao, Y.; Truhlar, D. G. The M06 Suite of Density Functionals for Main Group Thermochemistry, Thermochemical Kinetics, Noncovalent Interactions, Excited States, and Transition Elements: Two New Functionals and Systematic Testing of Four M06-Class Functionals and 12 Other Function. *Theor. Chem. Acc.* **2008**, *120* (1–3), 215–241.
S42. Sure, R.; Grimme, S. Comprehensive Benchmark of Association (Free) Energies of Realistic Host-Guest Complexes. *J. Chem. Theory Comput.* **2015**, *11* (8), 3785–3801.
S43. Barone, V.; Cossi, M. Conductor Solvent Model. *J. Phys. Chem. A* **1998**, *102* (97), 1995–2001.
S44. Young, T. A.; Martí-Centelles, V.; Wang, J.; Lusby, P. J.; Duarte, F. Rationalizing the Activity of an "Artificial Diels-Alderase": Establishing Efficient and Accurate Protocols for Calculating Supramolecular Catalysis. *J. Am. Chem. Soc.* **2020**, *142* (3), 1300–1310.
S45. Yousefi, R.; Sarkar, A.; Ashtekar, K. D.; Whitehead, D. C.; Kakeshpour, T.; Holmes, D.; Reed, P.; Jackson, J. E.; Borhan, B. Mechanistic Insights into the Origin of Stereoselectivity in an Asymmetric Chlorolactonization Catalyzed by $(DHQD)_2PHAL$. *J. Am. Chem. Soc.* **2020**, *142* (15), 7179–7189.
S46. Ranaghan, K. E.; Ridder, L.; Szefczyk, B.; Sokalski, W. A.; Hermann, J. C.; Mulholland, A. J. Insights into Enzyme Catalysis from QM/MM Modelling: Transition State Stabilization in Chorismate Mutase. *Mol. Phys.* **2003**, *101* (17), 2695–2714.
